# Lineage‐Specific CYP80 Expansion and Benzylisoquinoline Alkaloid Diversity in Early‐Diverging Eudicots

**DOI:** 10.1002/advs.202309990

**Published:** 2024-03-13

**Authors:** Zhoujie An, Ranran Gao, Shanshan Chen, Ya Tian, Qi Li, Lixia Tian, Wanran Zhang, Lingzhe Kong, Baojiang Zheng, Lijun Hao, Tianyi Xin, Hui Yao, Yu Wang, Wei Song, Xin Hua, Chengwei Liu, Jingyuan Song, Huahao Fan, Wei Sun, Shilin Chen, Zhichao Xu

**Affiliations:** ^1^ Key Laboratory of Saline‐alkali Vegetation Ecology Restoration (Northeast Forestry University) Ministry of Education Harbin 150040 China; ^2^ College of Life Science Northeast Forestry University Harbin 150040 China; ^3^ Key Laboratory of Beijing for Identification and Safety Evaluation of Chinese Medicine Institute of Chinese Materia Medica China Academy of Chinese Medical Sciences Beijing 100700 China; ^4^ School of Pharmaceutical Sciences Guizhou University Guiyang 550025 China; ^5^ Key Lab of Chinese Medicine Resources Conservation, State Administration of Traditional Chinese Medicine of the People's Republic of China, Institute of Medicinal Plant Development Chinese Academy of Medical Sciences & Peking Union Medical College Beijing 100193 China; ^6^ College of Life Science and Technology Beijing University of Chemical Technology Beijing 100029 China; ^7^ Institute of Herbgenomics Chengdu University of Traditional Chinese Medicine Chengdu 611137 China

**Keywords:** benzylisoquinoline alkaloids, biosynthetic pathway, chemo‐diversity, Menispermum, stereospecificity

## Abstract

Menispermaceae species, as early‐diverging eudicots, can synthesize valuable benzylisoquinoline alkaloids (BIAs) like bisbenzylisoquinoline alkaloids (bisBIAs) and sinomenines with a wide range of structural diversity. However, the evolutionary mechanisms responsible for their chemo‐diversity are not well understood. Here, a chromosome‐level genome assembly of *Menispermum dauricum* is presented and demonstrated the occurrence of two whole genome duplication (WGD) events that are shared by Ranunculales and specific to *Menispermum*, providing a model for understanding chromosomal evolution in early‐diverging eudicots. The biosynthetic pathway for diverse BIAs in *M. dauricum* is reconstructed by analyzing the transcriptome and metabolome. Additionally, five catalytic enzymes – one norcoclaurine synthase (NCS) and four cytochrome P450 monooxygenases (CYP450s) – from *M. dauricum* are responsible for the formation of the skeleton, hydroxylated modification, and C‐O/C‐C phenol coupling of BIAs. Notably, a novel leaf‐specific MdCYP80G10 enzyme that catalyzes C2′‐C4a phenol coupling of (*S*)‐reticuline into sinoacutine, the enantiomer of morphinan compounds, with predictable stereospecificity is discovered. Moreover, it is found that *Menispermum*‐specific CYP80 gene expansion, as well as tissue‐specific expression, has driven BIA diversity in Menispermaceae as compared to other Ranunculales species. This study sheds light on WGD occurrences in early‐diverging eudicots and the evolution of diverse BIA biosynthesis.

## Graphical abstract

Herein, a chromosome‐level genome assembly of *Menispermum dauricum* is presented. A novel MdCYP80G10 enzyme is identified to catalyze C2′‐C4a phenol coupling of (*S*)‐reticuline into sinoacutine, the enantiomer of morphinan compounds, with predictable stereospecificity. *Menispermum*‐specific CYP80 expansion has driven benzylisoquinoline alkaloids (BIA) diversity in Menispermaceae as compared to other early‐diverging eudicots.

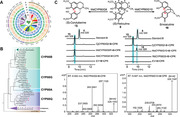

## 1 Introduction

Benzylisoquinoline alkaloids (BIAs), which represent a structurally diverse class of nitrogen‐containing, plant specialized metabolites widely distributed in early‐diverging eudicots,^[^ ^1^ ^]^ possess notable physicochemical and biological properties, as well as pharmaceutical activities such as the analgesic activity of morphine and codeine,^[^ ^2^ ^]^ the antiviral activity of tetrandrine and cepharanthine against SARS‐CoV‐2,^[^ ^3^, ^4^ ^]^ the anti‐arthritic effect of sinomenine,^[^ ^5^ ^]^ and drug efficacy of Dau‐d4 (a derivative of daurisoline) in improving glucose metabolism.^[^ ^6^ ^]^ The assorted carbon‐carbon (C‐C) and carbon‐oxygen (C‐O) phenol coupling reactions fundamentally contribute to the diverse types of BIAs, such as morphinans (e.g., morphine, codeine, thebaine), aporphines (e.g., magnoflorine), sinomenines (e.g., sinoacutine, sinomenine), and bisbenzylisoquinoline alkaloids (bisBIAs;, e.g., tetrandrine, cepharanthine).^[^ ^7^, ^8^ ^]^ Although the early stages of BIA biosynthesis are highly conserved among BIA‐producing species, the distribution of certain BIAs is lineage‐specific. For instance, morphinans, papaverine, and noscapine are primarily found in *Papaver* species,^[^ ^9^, ^10^ ^]^ bisBIAs accumulate mainly in Menispermaceae and Nelumbonaceae species,^[^ ^11^ ^]^ and sinomenines are predominantly found in Menispermaceae species.^[^ ^12^, ^13^ ^]^ Interestingly, morphinans and sinomenines share a similar skeleton but are found in distant species, with sinomenine being the enantiomer of the morphinan skeleton.

Various BIAs are subject to hydroxylation, epoxidation, and other oxidation reactions via the catalysis of diverse cytochrome P450 monooxygenases (CYP450s) to form diverse BIA skeletons, leading to the substantial chemical diversity in the speciation process.^[^ ^9^, ^14^, ^15^ ^]^ CYP450 subfamily members are responsible for forming intramolecular C‐C and intermolecular C‐O phenol couplings of BIAs with broad substrate specificity, such as PsCYP719B1 from *Papaver somniferum* for morphinan biosynthesis,^[^ ^16^ ^]^ CjCYP80G2 from *Coptis japonica* for aporphine biosynthesis,^[^ ^17^ ^]^ BsCYP80A1 from *Berberis stolonifera*, and NnCYP80A (NnCYP80Q2) from *Nelumbo nucifera* for the formation of dimeric bisBIAs.^[^ ^18^, ^19^, ^20^ ^]^ The genomes of several species from early‐diverging eudicots have been elucidated, revealing important insights into the biosynthesis and evolution of BIAs, such as morphine and noscapine in opium poppy, and cavidines in *Corydalis*, which originated from the emergence of species‐specific gene clusters and tandem gene duplications.^[^ ^21^, ^22^, ^23^, ^24^, ^25^, ^26^, ^27^ ^]^ However, the biosynthesis of bisBIAs and sinomenines and the evolutionary mechanism of these lineage‐specific BIA accumulations remain largely unclear. Furthermore, genetic information for Menispermaceae species has not been reported, making it of great interest to compare the biosynthesis and specific accumulation of bisBIAs and sinomenines to unravel the origin and evolution of certain BIAs in this lineage.

Herein, we present the genome sequencing of *Menispermum dauricum*, a member of the Menispermaceae family. Using transcriptome and metabolome data, we dissected the BIA biosynthetic pathway and determined the catalytic activities of norcoclaurine synthase (NCS) and CYP450 enzymes related to the biosynthesis of Ranunculales‐conserved and *Menispermum*‐specific BIA compounds. Furthermore, we have identified lineage‐specific NCS and CYP450 gene duplication resulting from whole‐genome duplication (WGD) or tandem gene duplication (TGD) of biosynthetic genes. This gene expansion has contributed to the identification of novel C‐C and C‐O phenol coupling enzymes, and facilitated the structural diversity of BIAs, especially for bisBIAs and sinomenines, in early‐diverging eudicots.

## 2 Results

### 2.1 Genome Assembly and Annotation of *M*. *dauricum*

**Figure 1:**
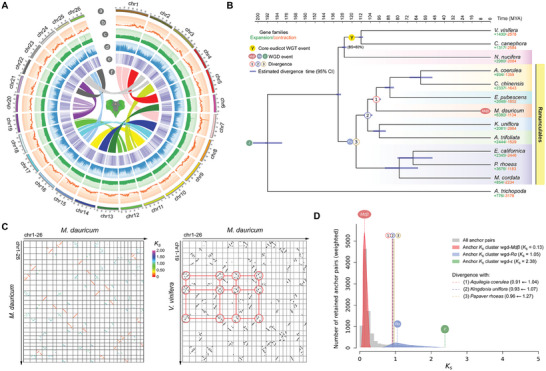
Genomic features, syntenic analysis, and phylogenetic positions of *M. dauricum*. A) Characteristics of the 26 chromosomes of *M. dauricum*. Tracks displayed are the karyotype (a), the distribution of gene counts (100 kb window, b), GC content (100 kb window, c), LTR content (100 kb window, d), gene density (100 kb window, e), and the syntenic blocks (f). B) Phylogenetic relationship and WGD events of *Menispermum* in the Ranunculales. The occurrence of gene family expansion and contraction is indicated by the green and red numbers with plus and minus signs, respectively. All nodes, except the ones indicated, received 100% bootstrap support (BS). C) Synteny blocks within *M. dauricum* and between *M. dauricum* and *V. vinifera*. The red circles show a 4:3 syntenic relationship between *M. dauricum* and grape. D) Distribution of synonymous substitution rates (*K_S_*) for the anchored paralogs of *M. dauricum* and the anchored orthologs between *M. dauricum* and *A. coerulea*, *M. dauricum* and *K. uniflora*, and *M. dauricum* and *P. rhoeas*. The colored dashed lines with letters (a, b, c) represent the WGD events, and the colored dashed lines with numbers (1, 2, 3) represent the divergences of *M. dauricum* with *A. coerulea*, *K. uniflora*, and *P. rhoeas*, respectively.

*M*. *dauricum* has an estimated genome size of 684.22 Mb with a relatively low level of heterozygosity (≈0.9%) based on the 25 *k*‐mer frequency distribution (Figure S1, Supporting Information). The genome of *M. dauricum* was sequenced using the third‐generation Sequel sequencing platform, generating 29.8 Gb (≈46 × coverage) of HiFi reads. The filtered HiFi reads (4077968) with an N50 length of 13.54 kb were directly assembled using Hifiasm, resulting in a primary genome assembly of 887.05 Mb, containing 11156 contigs with a contig N50 length of 4.85 Mb (Table S1, Supporting Information). After heterozygous contigs was removed, the genome assembly was reduced to 675.68 Mb, including 253 contigs, with an N50 length of 5.93 Mb and a longest contig length of 14.48 Mb (Table S1, Supporting Information). This draft genome assembly covered 98.75% of the estimated nuclear genome size with a GC content of 35.14% (Figure S1 and Table S1, Supporting Information). Chromosome conformation capture methodology (i.e., Hi‐C sequencing) was used for scaffolding, resulting in 250 contigs covering 648.03 Mb (95.9%) of the assembled genome, which were anchored onto 26 pseudochromosomes (2n = 52) (**Figure** **1**A; Figure S2 and Tables S1 and S2, Supporting Information). The genome assembly was evaluated using BUSCO, with 95.8% of the 1614 embryophyta single‐copy orthologs identified as complete, indicating the high quality and completeness of the genome assembly (Table S3, Supporting Information).

Approximately 63.78% (430972831 bp) of the genome was annotated as transposable elements (TEs), with 33.80% of TEs being long terminal repeat (LTR) retrotransposons (Table S4, Supporting Information). A total of 336888 LTR elements in the *M*. *dauricum* genome were identified, of which 173064 elements (24.11%) were from the *Copia* superfamily and 33885 elements (3.54%) were from the G*ypsy* superfamily (Table S4, Supporting Information). The quality of the *M. dauricum* genome assembly was further evaluated using the LTR assembly index (LAI), revealing a LAI value of 11.49, indicating that this genome assembly can serve as a reference standard, comparable to published genomes of other Ranunculales species (Tables S5 and S6, Supporting Information). In addition, 126050 simple sequence repeats were annotated, providing valuable molecular markers for future genetic diversity studies of *M*. *dauricum* (Table S4, Supporting Information). A total of 37236 protein‐coding genes were predicted using a combination of ab initio gene predictions, homologous proteins from other Ranunculales species, and the *de novo* assembled transcripts from the RNA‐Seq reads of *M*. *dauricum*. Complete orthologs for 93.5% of the embryophyta BUSCO dataset were identified, indicating that the predicted protein‐coding genes are largely complete (Table S3, Supporting Information).

### 2.2 Identification of the Lineage‐Specific WGD Event of *M. dauricum*

To investigate the evolution of genomes in the order Ranunculales, 159 single‐copy genes were identified from 13 angiosperms to construct phylogenetic tree topologies. The resulting phylogenetic relationships showed that Papaveraceae species (*Papaver rhoeas*, *Macleaya cordata*, and *Eschscholzia californica*) are sister to Circaeasteraceae (*Kingdonia uniflora*) + Menispermaceae (*M. dauricum*) + Ranunculaceae (*Aquilegia coerulea* and *Coptis chinensis*) + Berberidaceae (*Epimedium pubescens*) with 100% support, as expected (Figure 1B). In addition, *M. dauricum* was found to be the sister to Ranunculaceae and Berberidaceae species, and the clade comprising Menispermaceae + Berberidaceae + Ranunculaceae was found to be sister to Circaeasteraceae + Lardizabalaceae (bootstrap support, BS = 100%) (Figure 1B). Molecular dating, based on fossil age calibrations, revealed that the divergence between the Menispermaceae (*M. dauricum*) and Berberidaceae (*E. pubescens*) families occurred ≈99 million years ago (MYA), with a 95% confidence interval (CI) of 94.55 to 102.44 MYA (Figure 1B; Figure S3, Supporting Information). Furthermore, the Papaveraceae family was estimated to have diverged from the Circaeasteraceae, Lardizabalaceae, Menispermaceae, Berberidaceae and Ranunculaceae families at ≈116 MYA, with a 95% CI of 113.14 to 118.13 MYA (Figure 1B; Figure S3, Supporting Information). Finally, 6380 expanded and 1134 contracted families in *M. dauricum* were detected, and the rapid expansion gene families were enriched into the response to fugus, light, temperature, toxic, and radiation stimulus (Figure 1B; Figures S4 and S5, Supporting Information).

Intragenomic collinearity analysis based on synonymous substitutions per synonymous site (*K*_S_) was employed to investigate paralogous genes in collinear regions, revealing evidence for at least two WGD events during the evolutionary history of *M. dauricum* (Figure 1; Figure S6, Supporting Information). Intergenomic collinearity analyses between *M. dauricum* and other species, including *C. chinensis*, *A. coerulea*, and *P. rhoeas*, showed that four paralogous segments in the *M. dauricum* genome corresponded to two orthologous regions of these genomes (Figure S7, Supporting Information). Syntenic analysis between *M. dauricum* and *Vitis vinifera* showed a notable 4:3 relationships among orthologous segments, confirming the occurrence of two WGD events in *M. dauricum* (Figure 1C; Figure S7, Supporting Information). Distributions of *K*_S_ for all paralogous genes and for paralogous genes in collinear regions of *M. dauricum* exhibited two clear peaks at *K*_S_ ≈ 0.13 and 1.05, providing further support for the occurrence of two WGD events. The larger *K*_S_ values for *M. dauricum* (1.05), *C. chinensis* (1.06), *A. coerulea* (1.09), and *P. rhoeas* (1.11) suggest an ancient WGD event (*Rα*) for Ranunculales, whereas the smaller *K*_S_ value (0.13) for *M. dauricum* confirm the occurrence of a lineage‐specific WGD event (*Mdβ*) after the speciation of *M. dauricum*.

Based on the divergent times and *K*_S_ values for orthologous genes between *M. dauricum* and *A. coerulea* (98.55 MYA), *K. uniflora* (106 MYA), and *P. rhoeas* (115.91 MYA), we estimated the mutation rate as 4.29×10^−9^ synonymous substitutions per site per year for *M. dauricum*. Using this estimated mutation rate and *K*_S_ for paralogous genes of *M. dauricum*, the *M. dauricum* WGD events occurred at ≈122.31 MYA for the ancient WGD (*Rα*) and ≈15.14 MYA for the lineage‐specific WGD (*Mdβ*).

### 2.3 An Ideal Model for Studying Chromosomal Evolution in Early‐Diverging Eudicots

**Figure 2:**
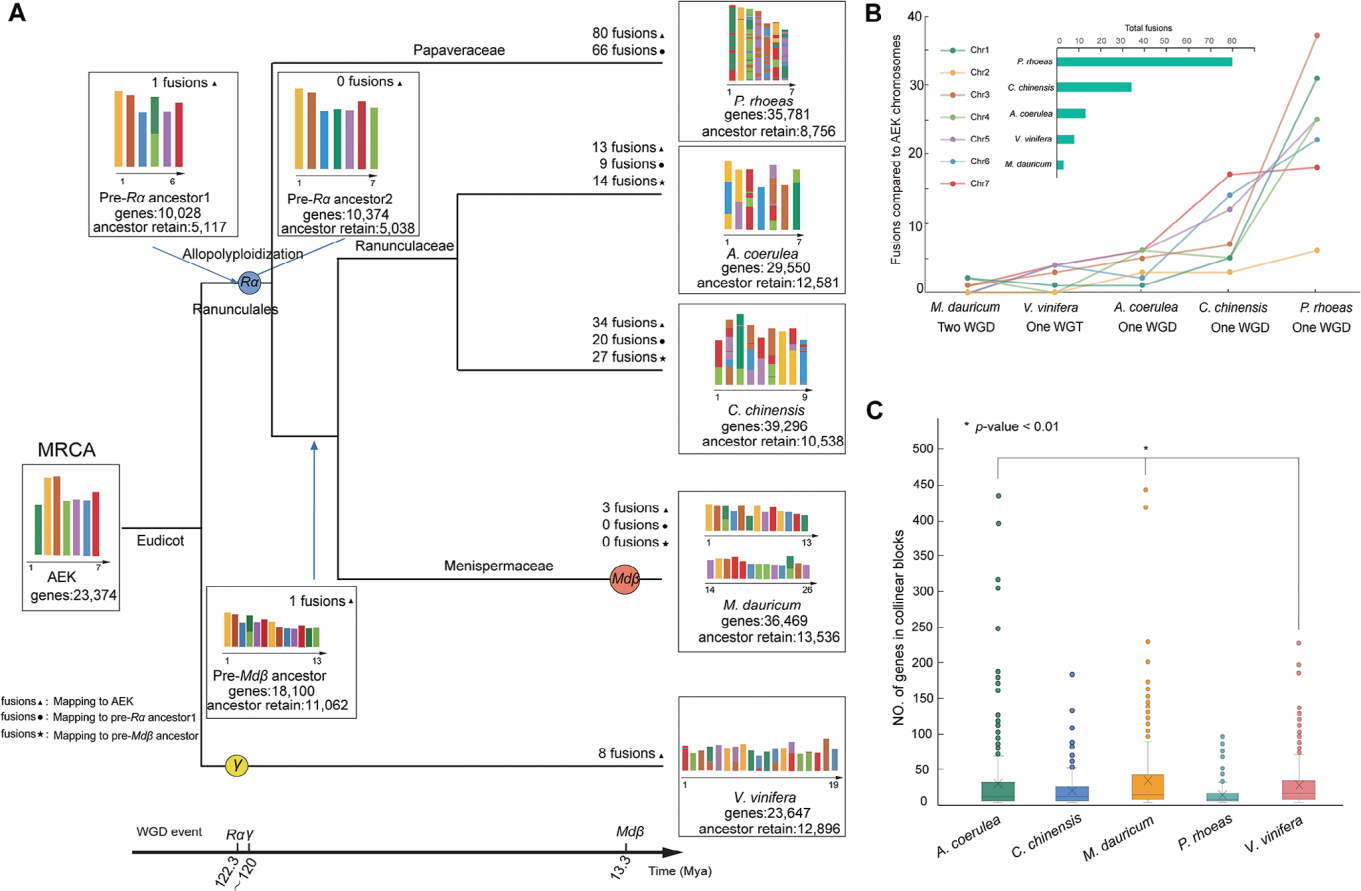
Ancestral genomes and chromosomal rearrangements among Ranunculales species. A) Inferred genome karyotypes of the pre‐*Mdβ* ancestor, pre‐*Rα* ancestor, and pre‐*Rα* ancestor1 based on the genome collinearity analysis. AEK represents the reported ancestor eudicot karyotype.^[^ ^85^ ^]^ All ancestral karyotypes and extant chromosomes were painted according to the seven AEK chromosomes. Chromosomal fusions were analyzed and calculated according to genome collinearity, and the triangle, circle, star, and rhombus represent the tested genome mapped to the AEK, pre‐*Rα* ancestor1 and pre‐*Mdβ* ancestor, respectively. B) Total number of fusions in extant species as compared to the AEK, and the number of fusions on each AEK chromosomes for *M. dauricum*, *C. chinensis*, *A. coerulea*, *P. rhoeas*, and *V. vinifera*, respectively. C) The number of ancestral genes localized in each of the syntenic blocks as compared to the AEK genome. One way analysis of variance (ANOVA) *p*‐value indicates the statistical significance (*p <0.01).

Herein, we have identified the two WGD events in *M. dauricum*: the Ranunculales‐shared *Rα* and lineage‐specific *Mdβ* duplications. Whereas ancestral protochromosomes of three *Papaver* species have been previously inferred,^[^ ^23^ ^]^ the ancestral Ranunculales genome remains poorly understood. Therefore, the genome collinearity among *M. dauricum* and other Ranunculales genomes was used to construct the ancestral karyotype and explore the evolution of representative Ranunculales species based on the ancestral eudicot karyotype (AEK) genome^[^ ^28^ ^]^ (**Figure** **2**A; Figures S8–S25, Supporting Information). We first inferred that the 26 *M. dauricum* chromosomes had been generated from 13 pre‐*Mdβ* ancestral chromosomes based on the genome collinearity analysis (Figure S8, Supporting Information). The pre‐*Mdβ* ancestral genome was determined as the ancestor shared by *Menispermaceae* and *Ranunculaceae* after the *Rα* polyploidy. Furthermore, the 13 pre‐*Mdβ* ancestral chromosomes were inferred into 6 and 7 chromosomes, referred to as pre‐*Rα* ancestor1 and pre‐*Rα* ancestor2, respectively (Figure 2A; Figure S9, Supporting Information). In addition, the genomes of *C. chinensis*, *A. coerulea*, and *M. dauricum* were respectively mapped into AEK genome to divide into subgenomes (Figure S10, Supporting Information). The subgenome A and B of *C. chinensis*, *A. coerulea*, and *M. dauricum*, which generated via the duplication of *Rα* event, are phylogenetically clustered into two distant branches (Figure S11, Supporting Information), supported that the duplication of pre‐*Rα* ancestor1 and pre‐*Rα* ancestor2 in the Ranunculales resulted from the allopolyploidization event.

Our genome collinearity analysis only identified three chromosomal fusion events in the *M. dauricum* genome compared to AEK (Figure S12, Supporting Information). Further comparison with other extant Ranunculales genomes (e.g., *C. chinensis*, *A. coerulea*, and *P. rhoeas*) and *V. vinifera* showed that chromosomal fusions in *P. rhoeas* and *C. chinensis* compared to AEK chromosomes occurred more frequently than in other tested species (Figure 2B; Figures S13–S16, Supporting Information). Additionally, we found that Chr 1 and Chr 3 of the ancestral AEK genome were substantially rearranged and enriched by fusion events to result in modern *P. rhoeas* chromosomes (Figure 2B; Figure S15, Supporting Information). Interestingly, *M. dauricum* exhibited the lowest number of chromosomal fusions as compared to AEK among all tested species, indicating its evolutionary conservation (Figure 2B and Table S7, Supporting Information). The number of ancestral genes localized in each of the syntenic blocks in *M. dauricum* was significantly larger than that in the *C. chinensis* and *P. rhoeas* genomes (*P* < 0.01) (Figure 2C and Data S1, Supporting Information). Furthermore, the number of ancestral genes retained in the *M. dauricum* genome was also larger than that in other tested species (Figure 2A). These results suggested that the ancestral AEK genome has been well preserved in the extant *M. dauricum* genome, even after two rounds of polyploidization events. Therefore, *M. dauricum* could serve as a valuable model for discovering ancestral traits and features of the Ranunculales plants, even for eudicots.

### 2.4 Tissue Specificity and Diversity of BIAs Accumulation in *M. dauricum*

Total alkaloids were extracted from various tissues of *M. dauricum*, including roots, root hairs, upper stems, lower stems, leaves, and young leaves, and were analyzed by high‐performance liquid chromatography (HPLC). There was substantial variation in BIA accumulation among roots (roots and root hairs), stems (upper stems and lower stems), and leaves (leaves and young leaves). Notably, three represented bisBIAs—guattegaumerine **26**, daurisoline **27**, and dauricine **28**—accumulated primarily in the roots and root hairs of *M. dauricum*, with retention times of 10.13, 11.46, and 11.59 min, respectively; however, sinomenine **19** accumulated in large amounts in aerial tissues, such as leaves and young leaves (Figure S26, Supporting Information).

**Figure 3:**
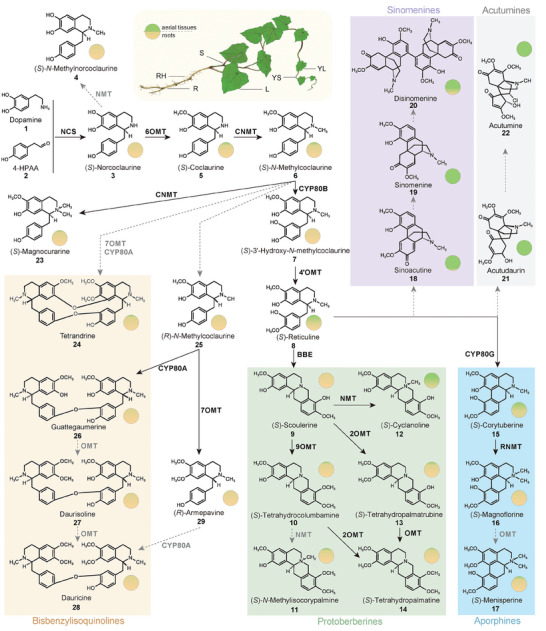
Accumulation of diverse BIAs and the proposed BIA biosynthetic pathway in *M. dauricum*. The shaded regions indicate the different BIA structural types, including bisBIAs, protoberberines, aporphines, sinomenines, and acutumines. The mean values of five replicates (n = 5 biologically independent samples) were used to calculate the relative proportions of BIAs accumulated in roots and aboveground tissues, indicated as yellow and green circles, respectively. Raw data were sourced from Data S2. RH (root hair), R (root), S (stem), L (leaf), YL (young leaf), and YS (young stem). Dashed arrows indicate unknown steps.

Based on the reported BIA biosynthetic pathway and chemical structures, we have proposed the BIA biosynthetic pathway in *M. dauricum* including upstream BIAs, bisBIAs, protoberberines, aporphines, sinomenines, and acutumines. To confirm this proposed pathway, targeted metabolomics analysis of different tissues using liquid chromatography‐tandem mass spectrometry (LC‐MS/MS) was performed, which identified a total of 27 BIAs (**Figure** **3**; Figures S27 and S28 and Table S8, Supporting Information). Notably, the specific accumulation of different BIA types in aerial tissues and roots of *M. dauricum* was observed. For instance, the relative content of the four bisBIAs (tetrandrine **24**, **26**, **27**, and **28**) in roots and root hairs was hundred times higher than in other tissues (Figure 3 and Data S2, Supporting Information). Additionally, protoberberines (scoulerine **9**, tetrahydrocolumbamine **10**, N‐methylisocorypalmine **11**, tetrahydropalmatrubine **13**, tetrahydropalmatine **14**) and aporphines (magnoflorine **16** and menisperine **17**) also exhibited a pattern of high root accumulation (Figure 3 and Data S2, Supporting Information). However, sinomenines (sinoacutine **18**, sinomenine **19**, disinomenine **20**) and acutumines (acutudaurin **21** and acutumine **22**), which are found specifically in the Menispermaceae, showed substantial accumulation in aerial tissues (Figure 3 and Data S2, Supporting Information). The tissue specificity and diversity of BIA accumulation in *M. dauricum* make the weighted gene co‐expression analysis a valuable strategy for integrating metabolome and transcriptome data (Figure S29, Supporting Information). Functional prediction of gene modules showed that the genes related to root‐accumulated bisBIAs are enriched in the defense response to fugus, bacterium, virus, and endogenous stimulus; however, the genes related to sinomenines from aerial tissues are enriched in the response to light and radiation stimulus (Figure S30, Supporting Information). Furthermore, we conducted a systematic investigation of core BIA biosynthetic genes from *M. dauricum*, which consisted of those that encode NCS and CYP450, with the aim of shedding light on the function of novel BIA biosynthetic genes and the molecular mechanism behind the chemodiversity of BIAs.

### 2.5 Functional Identification and Evolution of NCS Genes in *M. dauricum*

**Figure 4:**
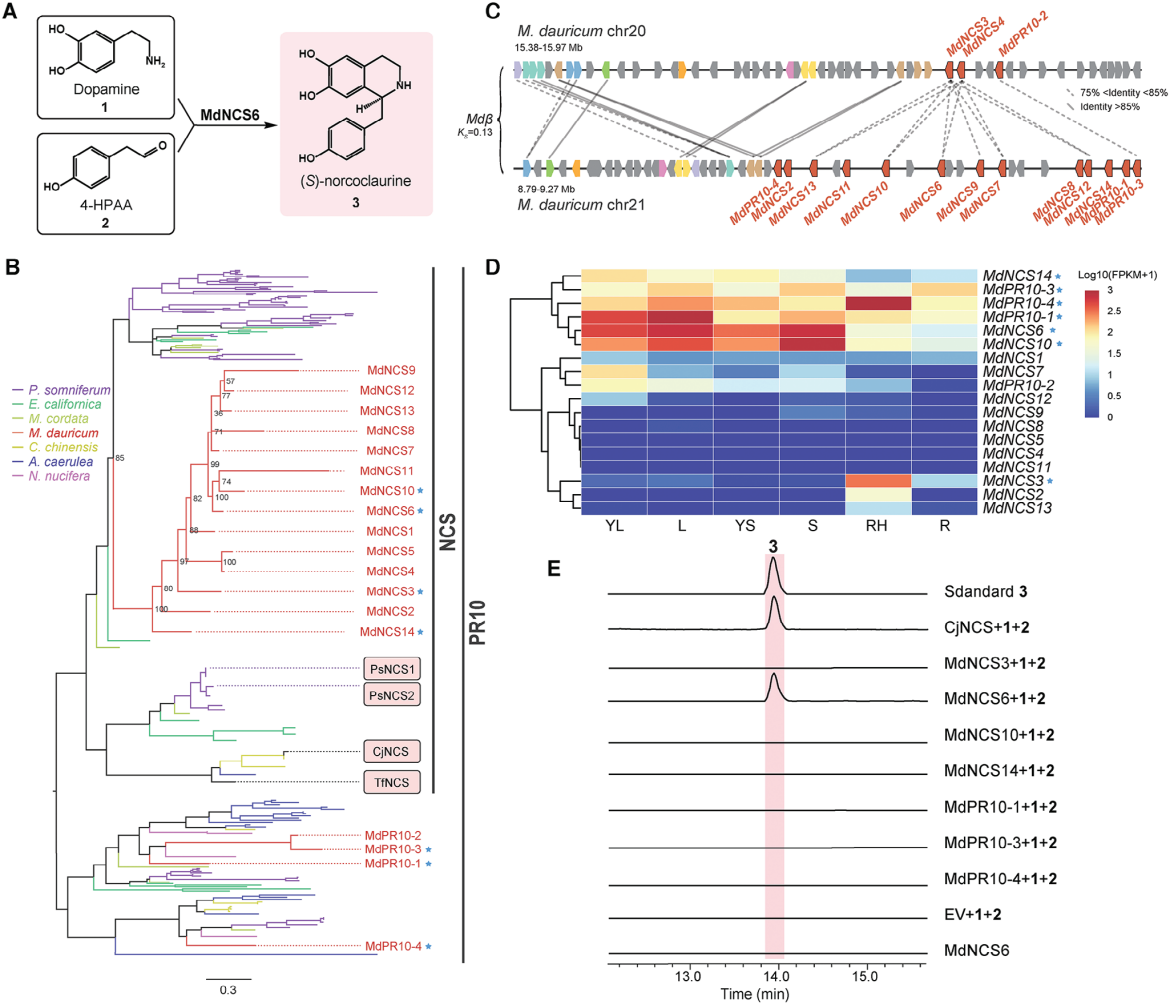
Phylogenetic analysis and functional identification of MdNCSs related to (*S*)‐norcoclaurine production. A) NCS catalyzes the condensation of dopamine 1 and 4‐HPAA 2 into (*S*)‐norcoclaurine 3 via the Pictet‐Spengler reaction. B) Phylogenetic tree of NCSs annotated from *M. dauricum*, *P. somniferum*, *E. californica*, *M. cordata*, *C. chinensis*, *A. coerulea*, and *N. nucifera*. The framed genes highlight the identified NCSs from other species. The blue stars indicate the selected genes for verifying their catalytic activity in this study. Branch support for MdNCSs is provided. C) Collinearity analysis of NCS‐encoding genes in *M. dauricum*. MdNCS genes are indicated in red. Dashed lines indicate that the identity between syntenic genes ranges from 75% to 85%, whereas solid lines indicate that the identity between paralogs is >85%. D) Expression pattern of MdNCSs in various tissues of *M. dauricum*, including young leaf (YL), leaf, young stem (YS), stem, root hair (RH), and root. E) In vitro catalytic assays of MdNCSs using an *E. coli* expression system. CjNCS from C. japonica and plasmid pMAL‐C5x (empty vector, EV) were chosen as the positive control and negative control, respectively.

NCS, encoded by PR10/Bet v1 family members, catalyzes the condensation of dopamine **1** and 4‐hydroxyphenylacetaldehyde (4‐HPAA) **2** to form the central precursor (*S*)‐norcoclaurine **3** of the BIA pathway, which is also a rate limiting step in BIAs biosynthesis (**Figure** **4**A). In this study, we annotated 18 PR10/Bet v1 genes from the genome of *M. dauricum*. The PR10/Bet v1 family members had undergone a substantial expansion in Ranunculales species, with only two, one, and four genes in *Amborella trichopoda*, *V. vinifera*, and *Coffea canephora*, respectively (Table S9, Supporting Information). Phylogenetic analysis indicated that 14 of the MdPR10/Bet v1 genes from *M. dauricum* clustered into one monophyletic group together with genes from Papaveraceae and Ranunculaceae species, and the PR10/Bet v1 genes in this clade might be specifically related to the NCS activity in Ranunculales (Figure 4B; Figure S31, Supporting Information). The phylogenomic tree showed a closer relationship between Menispermaceae and Ranunculaceae; however, the NCS‐derived phylogenetic tree indicated that *NCSs* from the Papaveraceae and Ranunculaceae species have a close relationship (Figure 4B), which might have resulted from the rapid expansion and evolution of *NCSs* in *M. dauricum*. Three and 13 MdPR10/Bet v1 genes were distributed on Chr20 and Chr21, respectively, with collinearity (Figure 4C). The *K*_S_ peak for 325 paralogous pairs of this syntenic block from Chr20 and Chr21 was ≈0.13, consistent with the *M. dauricum*‐specific *Mdβ* WGD event (Figure S32, Supporting Information), suggested that the recent WGD event could be related with the formation and radiation of BIA biosynthesis. The 13 PR10/Bet v1 genes on Chr21, which covered a DNA length of 292 kb, resulted from TGD. These results indicated that both the WGD and TGD events contributed extensively to the expansion of the MdNCSs.

The present study analyzed the expression patterns of 14 MdNCSs (referred to as MdNCS1 to MdNCS14) and four other PR10/Bet v1 genes (referred to as MdPR10‐1 to MdPR10‐4) in different tissues, including the aerial tissues and roots of *M. dauricum* (Figure 4D). Among these genes, those with high expression values (FPKM > 100) in any one tissue were selected and cloned, which included MdNCS3, MdNCS6, MdNCS10, MdNCS14, MdPR10‐1, MdPR10‐3, and MdPR10‐4. Subsequently, the in vitro catalytic activity of these candidate NCS proteins was assessed using dopamine **1** and 4‐HPAA **2** as substrates, with the NCS gene from C. japonica as the positive control. Notably, only one NCS protein, MdNCS6, was observed to produce a new peak at the retention time of 15.3 min, even though all candidate NCS proteins were expressed in *Escherichia coli* (Figure 4E; Figure S33, Supporting Information). Furthermore, both the HPLC and mass spectrum at *m/z* 272.1281 of the new product were identical to the reference standards of (*S*)‐norcoclaurine **3** (Figure 4E; Figure S34, Supporting Information). However, no products were detected in the assays of other MdNCSs. Importantly, whereas (*S*)‐norcoclaurine **3** accumulated predominantly in roots and root hairs, MdNCS6 exhibited low abundance in roots but high expression in stems (both upper and lower) and leaves (both mature and young leaves). These observations suggested that the transport of (*S*)‐norcoclaurine **3** from aerial tissues to the roots may occur simultaneously with its biosynthesis.

### 2.6 Gene Expansion and Functional Divergence of MdCYP80s

**Figure 5:**
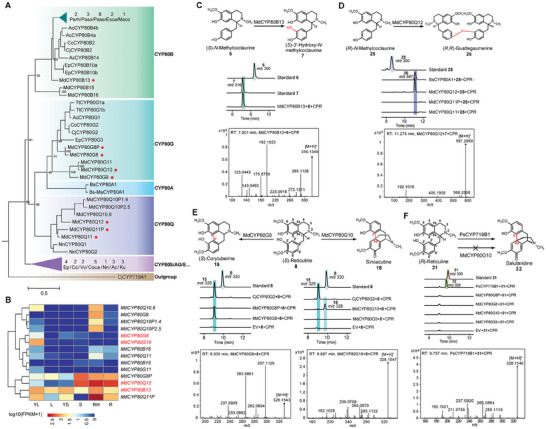
Phylogenetic analysis, gene expression pattern, and catalytic activities of MdCYP80 members. A) The phylogenetic relationship of CYP80s from *M. dauricum* and other tested species, including *A. coerulea* (Ac), *C. chinensis* (Cc), *C. japonica* (Cj), *E. pubescens* (Ep), *M. dauricum* (Md), *T. thalictroides* (Tt), *K. uniflora* (Ku), *B. stolonifera* (Bs), *P. somniferum* (Paso), *P. setigerum* (Pase), *P. rhoeas* (Parh), *M. cordata* (Mc), *N. nucifera* (Nn), *C. canephora* (Coca), *V. vinifera* (Vv). The red circles represent the functionally verified MdCYP80s in this study. Branch support for MdCYP80s is provided. B) Expression pattern of 14 MdCYP80 genes in various tissues of *M. dauricum*, including young leaf (YL), leaf (L), young stem (YS), stem (S), root hair (RH), and root (R). C) In vitro catalytic assays of MdCYP80B13 using (*S*)‐*N*‐methylcoclaurine **6** as the substrate. The LC‐MS extracted ion chromatograms at *m/z* 300 and *m/z* 316 indicate (*S*)‐*N*‐methylcoclaurine **6** and (S)−3′‐hydroxy‐*N*‐methylcoclaurine **7**, respectively. MS/MS fragmentation of (*S*)−3′‐hydroxy‐*N*‐methylcoclaurine **7** is shown. D) In vitro catalytic assays of MdCYP80Q12/5/6 using (*R*)‐*N*‐methylcoclaurine **25** as substrate, with BsCYP80A from *B. stolonifera* as positive control. The LC‐MS extracted ion chromatograms at m/z 300 and m/z 597 represent (*R*)‐*N*‐methylcoclaurine **25** and (*R*,*R*)‐guattegaumerine **26**, respectively. MS/MS fragmentation of (*R*,*R*)‐guattegaumerine **26** was detected. E) In vitro catalytic assays of MdCYP80G8P/2/4/5 using (*S*)‐reticuline **8** as substrate. The LC‐MS extracted ion chromatograms at *m/z* 330 and *m/z* 328 represent (*S*)‐reticuline **8** and (*S*)‐corytuberine **15** / sinoacutine **18**, respectively. (*S*)‐corytuberine **15** and sinoacutine **18** were detected separately based on MS/MS fragmentation. F) Catalytic analysis of MdCYP80G8P/8/10/9 using (*R*)‐reticuline **31** as substrate. The LC‐MS extracted ion chromatograms at *m/z* 330 and *m/z* 328 represent the (*R*)‐reticuline and the potential C‐C linked products, respectively. The empty vector pESC‐His was used as the negative control in **E** and **F**. MS/MS fragmentation of salutaridine **32**, which is the catalytic product of PsCYP719B1 activity with the substrate (*R*)‐reticuline **31**.

The CYP80 families contribute to the diversity of BIA biosynthesis through various reactions, such as hydroxylation, C‐C phenol coupling, C‐O phenol coupling, and dimerization of BIA unions. We annotated 14 *MdCYP80s*, which indicated a large expansion of CYP80 family members in *M. dauricum* relative to the other species examined, as they had two to eight annotated *CYP80s* (**Figure** **5**A; Figure S35, Supporting Information). Given the lineage‐specific biosynthesis of bisBIAs and sinomenines in Menispermaceae, we hypothesized that the expansion of the MdCYP80 family may be related to the formation of BIA diversity. To test this hypothesis, we performed functional assays on eight cloned *MdCYP80s*, including three *MdCYP80Qs*, four *MdCYP80Gs*, and one *MdCYP80B*, using a yeast expression system (Figure 5A; Figure S36, and Table S10, Supporting Information). We used (*S*)‐*N*‐methylcoclaurine **6**, (*R*)‐*N*‐methylcoclaurine **25**, and (*S*)‐reticuline **8** as substrates, respectively, with CjCYP80G2 from *C. japonica* and BsCYP80A1 from *B. stolonifera* serving as positive controls for the functional assays.

The LC‐MS/MS results indicated that the catalytic assays with the MdCYP80B13 produced a new peak with the same exact mass as (*S*)−3′‐hydroxy‐*N*‐methylcoclaurine **7** ([M+H]^+^ = 316.1549) using (*S*)‐*N*‐methylcoclaurine **6** as the substrate, which suggests the crucial role of MdCYP80B13 in catalyzing the C3′‐hydroxylation of (*S*)‐*N*‐methylcoclaurine to (*S*)−3′‐hydroxy‐*N*‐methylcoclaurine, the crucial precursor of (*S*)‐reticuline (Figure 5C). In addition, the transient expression of MdCYP80B13 in *Nicotiana benthamiana* also confirmed its hydroxylation activity toward (*S*)‐*N*‐methylcoclaurine (Figure S37, Supporting Information). The expression pattern of MdCYP80B13 showed high transcript abundance in both aerial tissues and roots, consistent with the accumulation pattern of (*S*)‐reticuline **8** (Figure 5B).

The catalytic assays with the three MdCYP80Qs (MdCYP80Q12, MdCYP80Q11P, and MdCYP80Q11) indicated that only MdCYP80Q12 could dimerize two molecules of (*R*)‐*N*‐methylcoclaurine **25** into guattegaumerine **26** ([M+H]^+^ = 597.2959) via C3′‐O‐C4′ phenol coupling, which is identical to the activity of BsCYP80A1 in yeast (Figure 5D). This phenol coupling activity of MdCYP80Q12 was also indicated using *N. benthamiana* transient expression system (Figure S37, Supporting Information). However, MdCYP80Q12 and BsCYP80A1 had a low level of identity (45.42%), and the phylogenetic tree showed that the BsCYP80A1 gene was more closely related to the CYP80G gene branch (Figure 5A), suggesting the independent evolution of BsCYP80A1 from *B. stolonifera* and MdCYP80Q12 from *M. dauricum*. In addition, MdCYP80Q12 was highly expressed in roots, root hairs, and stems, consistent with the accumulation of bisBIAs in *M. dauricum* (Figures 3 and 5B), indicating its contribution to specialized bisBIA biosynthesis.

### 2.7 MdCYP80G10 Converts (*S*)‐Reticuline into Sinoacutine with Stereospecificity

The catalytic assays of four MdCYP80Gs (MdCYP80G8P, MdCYP80G8, MdCYP80G10 and MdCYP80G9) using (*S*)‐reticuline **8** as substrates showed that MdCYP80G8 could catalyze C2′‐C8 phenol coupling, converting (*S*)‐reticuline **8** into (*S*)‐corytuberine **15** ([M + H]^+^ = 328.1543), which was consistent with the activity of CjCYP80G2 (Figure 5E). Additionally, MdCYP80G8P also present a slight activity for the production of (*S*)‐corytuberine **15**; however, the catalytic efficiency of MdCYP80G8 was much higher than that of MdCYP80G8P (Figure 5E). Interestingly, MdCYP80G10 produced a new LC‐MS/MS peak with almost the same exact mass as (*S*)‐corytuberine **15** ([M + H]^+^ = 328.1547), although it was observed at a different retention time (8.930 min for the product of MdCYP80G8 and CjCYP80G2, 9.687 min for the product of MdCYP80G10) (Figure 5E). Targeted fragmentation of the new product showed that the retention time and common fragment ions, notably [M + H]^+^ = 328, 285, 265, 239, and 192, were consistent with the standard for sinoacutine **18** (C2’‐C4a phenol coupling of (*S*)‐reticuline **8**), the enantiomer of *Papaver*‐specific salutaridine (C2′‐C4a phenol coupling of (*R*)‐reticuline **31**) (Figure 5E). These two types of phenol coupling activities catalyzed by MdCYP80G8 and MdCYP80G10 have also been confirmed using *N. benthamiana* transient expression system (Figure S37, Supporting Information).

Morphinan compounds are a class of natural products that are exclusive to *Papaver* species, and salutaridine is the crucial intermediate of morphinan biosynthesis. However, the enantiomers of salutaridine, such as sinoacutine and sinomenine, are specifically distributed in Menispermaceae. A previous study showed that PsCYP719B1 from opium poppy catalyzes the phenol‐coupling reaction of (*R*)‐reticuline to salutaridine.^[^ ^16^ ^]^ Here, we evaluated the catalytic activity of MdCYP80G8P, MdCYP80G8, MdCYP80G10, and MdCYP80G9 toward (*R*)‐reticuline, but no product was observed as compared with the strains harboring the pESC‐His empty vector (Figure 5F), suggested that MdCYP80G members specifically accept (*S*)‐reticuline as substrate. In addition, MdCYP80G10 was specifically expressed in fresh leaves (Figure 5B), which is consistent with the accumulation pattern of sinoacutine and sinomenine.

Although no CYP80G homologs exist in *N. nucifera* and *Papaver* genomes, the aporphine‐type BIAs in both lineages have been isolated.^[^ ^29^, ^30^ ^]^ Importantly, aporphine‐type BIAs in *N. nucifera* do not contain any hydroxyl or methyl groups at the C‐4′ and C‐3′ positions,^[^ ^29^ ^]^ suggesting that the aporphines in *N. nucifera* might originate from other BIA skeletons, not (*S*)‐reticuline. Therefore, we tested the substrate selectivity of MdCYP80G8 and MdCYP80G10 using (*S*)‐norcoclaurine **3** and (*S*)‐*N*‐methylcoclaurine **6**, respectively (Figure S38, Supporting Information). The results showed no observed catalytic products, indicating that CYP80G members catalyze Ranunculales‐specialized aporphines and sinomenines with strong substrate specificity.

## 3 Discussion

The BIA diversity in early‐diverging eudicots is directly correlated with plant evolution, as evidenced by the presence of morphine and noscapine in *Papaver*, berberine in *Coptis*, and tetrandrine and cepharanthine in *Menispermum* and *Stephania*. Although the biosynthesis and evolution of well‐known BIA compounds, such as noscapine, morphine, and berberine, have been elucidated, how BIA diversity developed remains unclear. The genomic sequencing of various Ranunculales species, including *Papaver*,^[^ ^23^ ^]^ *Macleaya*,^[^ ^27^ ^]^ and *Corydalis* ^[^ ^31^ ^]^ from Papaveraceae; *Coptis*,^[^ ^25^ ^]^ *Thalictrum*,^[^ ^32^ ^]^ and *Aquilegia* ^[^ ^33^ ^]^ from Ranunculaceae; *Epimedium* ^[^ ^34^ ^]^ from Berberidaceae; *Kingdonia* ^[^ ^35^ ^]^ from Circaeasteraceae; and *Akebia* ^[^ ^36^ ^]^ from Lardizabalaceae, provides a critical foundation for comparative genomic studies aimed at understanding the biosynthesis and evolution of BIA diversity across different families. In this study, the high‐quality genome of *Menispermum* has helped fill a significant gap in the genomic information available for Menispermaceae, and thus presents a unique opportunity for exploring the molecular mechanisms underlying BIA diversity, particularly the specific evolution of bisBIAs and sinomenines in Menispermaceae.

Polyploidy, or WGD events, are widely recognized as a notable evolutionary driver for speciation, environmental adaptation, and diversification. In the *Menispermum* genome, we identified two rounds of WGD events, and the molecular dating analysis revealed that the older WGD event occurred ≈122 MYA, which is close to the gamma triplication event observed in core eudicots, estimated to have happened ≈117 MYA.^[^ ^37^ ^]^ The previous genome analysis of *A. coerulea* and *A. trifolita* identified similar genomic fusion events with *V. vinifera* according to genome synteny and *K*_S_ analysis, and proposed a shared WGD in both core eudicots and early‐diverging eudicots.^[^ ^36^, ^38^ ^]^ Conversely, the *K*_S_ peak corresponding to this “shared WGD” is absent in *Nelumbo*, which raises doubt about their hypothesis of the common tetraploid origin.^[^ ^39^, ^40^ ^]^ The reconstruction of AEK karyotype using genomes of early‐diverging eudicots revealed that the similar fusion between the Chr5 of *A. coerulea* and Chr 7 of *V. vinifera*, is absent in other early‐diverging eudicots and core eudicots, proposed that the WGD events between early‐diverging eudicots and core eudicots might be independently occurred.^[^ ^28^ ^]^ The comparative genomes among *Corydalis* and other early‐diverging eudicots identified and traced their WGD events, including the Ranunculales‐shared WGD event (*Rα*), *Nelumbo*‐specific WGD event, and the core eudicots‐shared WGT event, via adjusting the various substitution rate.^[^ ^26^ ^]^ Here, we also constructed the adjusted *K*_S_ distributions of paralogs and orthologs from *Menispermum*, *Aquilegia*, *Kingdonia*, and *Papaver* genomes, our results could well support the occurrence of an ancestor Ranunculales shared WGD event. The AEK reconstruction and phylogenetic analysis among ancestor subgenomes determined that the ancient *Rα* WGD event is likely allopolyploidization. However, the placement of ancient *Rα* WGD and gamma triplication event still needs to be further resolved based on the development of more appropriate models and eudicots genome sequencing.^[^ ^41^ ^]^ In addition, we also noted a lineage‐specific WGD event (*Mdβ*) in this genome. Yang et al. showed that recently occurring species‐specific WGD events in *Papaver* positively promoted chromosomal rearrangements.^[^ ^23^ ^]^ In contrast, the *Menispermum* genome displays a critically conserved evolution with fewer chromosomal rearrangements and longer syntenic blocks than other genomes within Ranunculales, even after the *Menispermum*‐specific WGD event. Therefore, the *Menispermum* genome could serve as an important model for understanding the evolution of Ranunculales species and BIA biosynthesis.

The presence of chiral centers in many BIAs results in stereoisomerism, including the (*S*)‐ and (*R*)‐enantiomers of *N*‐methylcoclaurine and reticuline.^[^ ^42^, ^43^ ^]^ For example, berberine, magnoflorine, and sinomenine are biosynthesized via (*S*)‐reticuline, whereas morphine originates from (*R*)‐reticuline. In *P. somniferum*, a reticuline epimerase (REPI) fusion between CYP82Y2 and aldo‐keto reductase catalyzes the stereochemical inversion of (*S*)‐reticuline to (*R*)‐reticuline, which CYP719B1 then converts into salutaridine, the precursor of morphine.^[^ ^16^ ^]^ Our metabolome results showed that the aerial tissues of *M. dauricum* accumulate the enantiomers of morphinan compounds, sinomenines; however, no homologs of REPI enzymes were found in this plant or other non‐*Papaver* species via genomic mining. Concerning the potential phenol‐coupling reaction of CYP719B orthologs in *M. dauricum*, we identify only two homologs, *MdCYP719C5a* and *MdCYP719C5b*, from its genome (Figure S39, Supporting Information). Here, the coding sequence of *PsCYP719B1* was synthesized and expressed in both heterologous yeast and tobacco systems. The catalytic reaction supported the previous study that it selectively accepted (*R*)‐reticuline as its substrate, whereas (*S*)‐reticuline was not accepted (Figure 5F; Figure S40, Supporting Information). The yeast expression system was used to carry out catalytic assays for both *MdCYP719C* genes, but no catalytic product was detected using either (*R*)‐reticuline or (*S*)‐reticuline as a substrate (Figure S40, Supporting Information). These findings suggest the critical substrate specificity and stereospecificity of PsCYP719B1 and MdCYP80G10 for (*R*)‐reticuline and (*S*)‐reticuline, respectively. The oxidative mechanism of the phenol‐coupling reaction of (*R*)‐reticuline **31** and (*S*)‐reticuline **8** under the catalysis of PsCYP719B1 and MdCYP80G10, respectively, was proposed based on a previous study.^[^ ^16^ ^]^ The formation of salutaridine **32** or sinoacutine **18** involves a single cycle of iron oxidation (Figure S41, Supporting Information). We propose that PsCYP719B1 and MdCYP80G10 form a radical at oxygen atoms through abstraction of a hydrogen from the C3′‐OH of (*R*)‐reticuline and (*S*)‐reticuline, respectively.^[^ ^16^ ^]^ Subsequently, C7‐OH undergoes dehydrogenation via radical‐pairing interactions. Finally, the oxygen atoms on the benzyl group undergo electron rearrangement and form new C2′‐C4a bonds intramolecularly.^[^ ^16^, ^44^ ^]^

Gene duplication events, particularly WGD and TGD, contribute to the evolution of biosynthetic genes involved in the production and diversity of plant specialized metabolites.^[^ ^45^, ^46^, ^47^, ^48^, ^49^, ^50^, ^51^, ^52^, ^53^ ^]^ For instance, the TGD and functional divergence of *N*‐methyltransferase in caffeine biosynthesis, neofunctionalization of carotenoid cleavage dioxygenases related to crocin biosynthesis,^[^ ^47^ ^]^ and WGD in *Aesculus* genus have been shown to contribute to the formation of aescins.^[^ ^49^ ^]^ Biosynthetic genes for BIAs are scattered throughout most plant genomes, except for the noscapine biosynthetic gene cluster in *P. somniferum*.^[^ ^22^, ^54^ ^]^ TGD of BIA biosynthetic genes, such as NCS, OMT, and BBEL genes, have been implicated in the diversity of BIAs. For example, the TGD and neofunctionalization of BBEL genes in the *Corydalis*‐specific biosynthesis of cavidines.^[^ ^26^ ^]^ Given the specific accumulation of salutaridine and morphine in *Papaver* species, our phylogenetic tree of CYP719 members from early‐diverging eudicot genomes showed that the CYP719B members present the *Papaver*‐specific evolution and expansion (Figure S39, Supporting Information). In addition, the expansion of CYP80G members through tandem gene duplication is unique to *M. dauricum* (Figure S42, Supporting Information). Our findings suggest that the formation of sinomenine derivatives and morphinan compounds in distant plant lineages might be the result of independent evolution of specialized biosynthetic pathways, with CYP80G10‐dependent sinoacutine biosynthesis from (*S*)‐reticuline in *Menispermum* and CYP719B1‐dependent salutaridine biosynthesis from (*R*)‐reticuline in *Papaver*, opening the door to understanding the divergence of the biosynthetic pathways of these two backbone compounds.

In conclusion, the genome sequencing of *M. dauricum* has provided a valuable genetic resource for investigating polyploidy in early‐diverging eudicots and exploring the convergent and divergent evolution of highly valuable BIAs in Ranunculales. We also functionally characterized one MdNCS involved in forming the BIA structural skeleton, as well as observed lineage‐specific expansion of MdCYP80 genes and functional divergence in the biosynthesis of specialized bisBIAs and sinomenines in Menispermaceae species. Additionally, our comparative genome analysis has revealed the independent evolution of morphinan biosynthesis in *Papaver* and sinomenine biosynthesis in *Menispermum*, which originated from the functional convergence of distant CYP450 families for (*S*)‐ and (*R*)‐enantiomers of reticuline. Overall, our study provides new insights into the biosynthesis and evolution of diverse BIAs in Ranunculales.

## 4 Experimental Section

### Plant Materials and Chemical Standards

*M. dauricum* plants were obtained from the Institute of Medicinal Plant Development, the Chinese Academy of Medical Sciences, Beijing, China. The species was identified and confirmed as *M. dauricum* using DNA barcoding technology.^[^ ^55^ ^]^ Various tissues, including young leaves (YL), young stems (YS), leaves (L), stems (S), root hairs (RH) and roots (R), were collected for transcriptome sequencing and metabolome analysis.

Chemical standards related to BIA biosynthesis were purchased from commercial chemical companies, which were listed in Table S11 (Supporting Information).

### Genome Sequencing and Assembly

High‐quality total genomic DNA was extracted from fresh leaves of an individual *M. dauricum* plant, which was subsequently used to construct different libraries for genome sequencing. Short insert fragments for Illumina sequencing and long fragments for SMRT sequencing were used for genome sequencing. The short‐read libraries were constructed and sequenced using the Illumina HiSeq X Ten platform. For long‐read sequencing, the libraries were constructed and sequenced using two cells of the PacBio Sequel platform (https://www.pacb.com/). Raw data were filtered to remove adapters and low‐quality reads using SMRT Portal analysis. A Hi‐C library of young *M. dauricum* leaves was also prepared following the standard procedure^[^ ^56^ ^]^ and was subjected to paired‐end sequencing. The cross‐linked and lysed cells for Hi‐C library were digested using Hind III restriction enzyme.

The genome size of *M. dauricum* was estimated using the 25 *k*‐mer distribution with the short‐reads data. Draft genome assembly was performed using Hifiasm (v0.16.0‐r369).^[^ ^57^ ^]^ The completeness of the *M. dauricum* genome assembly was estimated using the Embryophyta odb 10 dataset with Benchmarking Universal Single‐Copy Orthologs (BUSCO, v5.2.2).^[^ ^58^ ^]^ To improve the genome assembly, the paired‐end Hi‐C reads were mapped to the draft assembly, and the contigs were anchored into pseudo‐chromosomes using the ALLHiC pipeline.^[^ ^59^ ^]^

### Genome Annotation and Gene Expression Analysis

To predict and classify repeat elements in the *M. dauricum* genome, we used the RepeatModeler (v2.0.2) package, which includes two *de novo* repeat finding programs, RECON (v1.08) and RepeatScout (v1.0.6). Additionally, Genometools (v1.6.2) and LTR_retriever (v2.9.0) were used to identify and classify long terminal repeat (LTR) retrotransposons, and RepeatMasker (v4.0.6) was used to mask total TE sequences.^[^ ^60^ ^]^ The transcriptome data from various tissues were assembled *de novo* using Trinity (v2.8.5) with default parameters.^[^ ^61^ ^]^ The MAKER (v3.1.3) annotation pipeline was used to predict putative protein‐coding genes with parameters as described.^[^ ^62^ ^]^ Gene prediction species model were trained using Augustus (v3.4.0).^[^ ^63^ ^]^ The unigenes and predicted protein sequences from RNA‐Seq of *M. dauricum*, and the annotated protein sequences from *A. coerulea* and *C. chinensis* were combined as expressed sequence tags and protein homology evidence for BLAST and Exonerate alignments. The completeness of the genome annotation was evaluated using BUSCO (v5.2.2). The RNA‐Seq data from different tissues of *M. dauricum* were aligned to the genome using HISAT2 (v2.2.1)^[^ ^64^ ^]^ and the read counts and FPKM values were calculated and normalized using R packages. The WGCNA (v1.69) network was inferred by integrating gene expression and BIA accumulation in different tissues of *M. dauricum*, and GO and KEGG enrichment analysis were performed on the resulting WGCNA modules.^[^ ^65^ ^]^

### Phylogenetic Tree Construction and Phylogenomic Dating

OrthoFinder (v2.2.7)^[^ ^66^ ^]^ was used to identify orthogroups with the default parameters. The orthologs were obtained from two core eudicots (*V. vinifera* ^[^ ^67^ ^]^ and *C. canephora*), 10 early‐diverging eudicots (*N. nucifera*,^[^ ^68^ ^]^ *M. cordata*, *E. californica*,^[^ ^69^ ^]^ *P. Rhoeas*, *A. trifoliata*, *K. uniflora*, *M. dauricum*, *E. pubescens*, *C. chinensis* and *A. coerulea*), and one early‐diverging angiosperm (*A. trichopoda* ^[^ ^70^ ^]^). The single‐copy genes in 13 vascular plants were concatenated, and the sequences were aligned and trimmed using MAFFT (v6.240)^[^ ^71^ ^]^ and trimAI (v1.2).^[^ ^72^ ^]^ The species tree was constructed using RAxML (v8.2.9)^[^ ^73^ ^]^ for protein sequences with the PROTGAMMAJTT model. The evolutionary timescale was analyzed using MCMCtree of the PAML package^[^ ^74^ ^]^ with 50 000 iterations and a sample frequency of 10 after 200 000 iterations as the burn‐in. The divergence times of the species were estimated based on the following fossil‐based age constraints: *A. coerulea* and *C. chinensis* diverged 26–80 MYA, *C. chinensis* and *E. pubescens* diverged 76–103 MYA, *E. pubescens* and *M. dauricum* diverged 76–103 MYA; Ranunculaceae and Berberidaceae diverged 84–97 MYA, Ranunculales and *N. nucifera* diverged 126–132 MYA; *A. trichopoda* and other angiosperms divergence time of 180–205 MYA. CAFÉ (v5.0) was used to predict gene family evolution, including gene expansion and contraction.^[^ ^75^ ^]^

### Identification of WGD Events

The intra‐ and inter‐genomic comparisons among *M. dauricum*, *A. coerulea*, *C. chinensis*, *P. rhoeas*, and *V. vinifera* were performed using the Whole‐Genome Duplication Identifier (WGDI) pipeline.^[^ ^76^ ^]^ *K*_S_‐based age distributions for paralogous genes and anchored‐paralogs of the early‐diverging eudicot genomes (*M. dauricum*, *A. coerulea*, *C. chinensis*, *K. uniflora*, and *P. rhoeas*) and the *V. vinifera* genomes were constructed using “wgd” pipeline^[^ ^77^ ^]^ and i‐ADHoRe (v3.0),^[^ ^78^ ^]^ with the default parameters. Similarly, *K*_S_‐based age distributions for orthologous genes between between *M. dauricum* and *A. coerulea*, *K. uniflora*, and *P. rhoeas* were also estimated. To address potential inaccuracies in WGD event detection stemming from differing substitution rates among candidate species, ksrates (v1.1.3)^[^ ^79^ ^]^ was used to position adjusted WGD events via rate‐adjusted mixed paralog–ortholog *K*_S_ distributions for *M. dauricum*, *A. coerulea*, *K. uniflora*, and *P. rhoeas*.

### Chromosomal‐Rearrangement and Ancestral Karyotype Reconstruction

The identification of collinear genomic blocks among the genomes of *V. vinifera*, *M. dauricum*, and three other Ranunculales (*A. coerulea*, *C. chinensis*, and *P. rhoeas*) using the WGDI pipeline (v0.6.4)^[^ ^76^ ^]^ facilitated the reconstruction of the ancestral core‐eudicot karyotype. Two WGD events of the *M. dauricum* genome, *Rα* and *Mdβ*, were used to infer the chromosomes of the pre‐*Mdβ* ancestor and the karyotypes of the pre‐*Rα* ancestors. Homologous genes in *M. dauricum* were identified using BLASTP with an E‐value cutoff of 1e^−5^. The genome collinearities were then plotted with the “‐d” parameter of WGDI, and the grouping of chromosomes was optimized according to the collinear blocks. To further confirm the ancestral chromosomes karyotypes of pre‐*Rα*, the “‐pc” parameter of WGDI was used to classify the three species into subgenomes, and mapped these subgenomes to the AEK chromosomes using the “‐a” parameter to obtain collinear genes. Each chromosome of subgenomes was used to construct the coalescent tree using ASTRAL (v5.7.8).^[^ ^80^ ^]^ Finally, the ancestral eudicot karyotype (AEK) was used to mapping the extant genomes to calculate the fusion events.

### Accumulation of BIAs in Different Tissues of *M. dauricum*

Six different tissues of *M. dauricum*, specifically roots, root hairs, stems, young stems, leaves, and young leaves, were collected and subjected to drying at a constant temperature of 40 °C. The resulting samples were weighed (30 mg) and sonicated at room temperature for 1 h in 1.5 mL of 75% aqueous methanol containing 5 µg mL^−1^ umbelliferone as an internal standard.

The HPLC analysis of the BIAs was performed using a Shimadzu LC‐2050C 3D system. Each sample was injected at a volume of 5 µL, and the detection wavelength was set at 282 nm. Separation of the BIAs was achieved using a Hypersil GOLD^TM^ C18 column (5 µm, 4.6 × 250 mm) maintained at a temperature of 35 °C. The mobile phase consisted of two components: mobile phase A (0.1% formic acid) and mobile phase B (methanol). The gradient program was set at a flow rate of 0.5 mL min^−1^ and proceeded as follows: 0–1 min, 5% B; 1–3 min, 5%–30% B; 3–15 min, 30%–60% B; 15–20 min, 60%–100% B; 20–25 min, 100–5% B; and 25–30 min, 5% B.

For the relative quantification of the BIAs, an ultra‐performance liquid chromatography‐tandem mass spectrometry (UPLC‐MS/MS) system (SCIEX TripleTOF 6600+) was used. After centrifugation of the samples at 12 000 rpm for 10 min, the supernatant was filtered through a 0.22 µm hole diameter and stored for UPLC‐MS/MS analysis. Each sample was injected at a volume of 1 µL and was separated using a Kinetex C18 100A analytical column (4.6 mm × 150 mm, 2.6 µm) maintained at a temperature of 30 °C. The mobile phase consisted of mobile phase A (0.1% formic acid in water) and mobile phase B (acetonitrile). The gradient program was set at a flow rate of 0.4 mL min^−1^ and proceeded as follows: 0–1 min, 10% B; 1–11 min, 10–95% B; 11–12.5 min, 95% B; 12.5–12.51 min, 95%–10% B; and 12.51–13 min, 10% B. The mass spectrometer was operated in full scan mode with a scan time of 35 ms per transition. The parameters for mass spectrometer were set as the follows: electrospray ionization (ESI) mode in positive ion mode; spray voltage, 3.5 kV; spray temperature, 550 °C; curtain gas, 35 psi; GAS1, 40 psi; GAS2, 60 psi.

### Selection and Functional Identification of NCS Encoding Genes

The NCS genes, which are part of the PR10/Bet v1 family, were identified in several plant species including *M. dauricum*, *P. somniferum*, *E. californica*, *M. cordata*, *C. chinensis*, *A. caerulea*, and *N. nucifera* using BLASTp with an E‐value of 1e^−5^. The PsNCS1 (AAX56303.1) and PsNCS2 (AAX56304.1) from *P. somniferum*,^[^ ^1^ ^]^ CjNCS (BAF45338.2) from *C. japonica*,^[^ ^81^ ^]^ and TfNCS (ACO90248.1) from *Thalictrum flavum* were used as reference sequences. The selected genes were then aligned using MAFFT alignment software (v6.240) with default parameter,^[^ ^82^ ^]^ and the alignment was used to construct a maximum likelihood (ML) phylogenetic tree using IQ‐TREE (v2.0.3).^[^ ^83^ ^]^ Based on the phylogenetic position of previously reported *NCS* genes, the *NCS* clade from the candidate plant species was annotated.

The putative *NCS* genes were cloned into the pMAL‐C5x expression vector, which was then transformed into the *E. coli* BL21(DE3) strain for expression. The NCS proteins were purified through MBP affinity chromatography with Dextrin Beads resin (Smart‐Lifesciences, China). The CjNCS was used as the positive control, whereas the empty vector was used as the negative control. Enzymatic assays were performed at 37 °C and 200 rpm overnight in a reaction system (140 µL) containing Tris‐HCl buffer with 10 µL of the purified enzyme and 0.7 mg mL^−1^ of substrates (dopamine and 4‐HPAA). The reaction was quenched by adding 50% (v/v) MeOH and analyzed using HPLC and LC‐MS/MS.

HPLC analysis was conducted on a Shimadzu LC‐2050C 3D system equipped with a Hypersil GOLD^TM^ C18 column (5 µm, 4.6 mm × 250 mm). The mobile phase consisted of 0.1% formic acid in water (A) and acetonitrile (B). A linear gradient elution program was used as follows: 0–10 min, 5% B; 10–15 min, 5%−20% B; 15–15.1 min, 20%−95% B; 15.1–25 min, 95%−5% B; 25.1–30 min 5% B (flow rate, 0.5 mL min^−1^; column temperature, 35 °C). UV absorbance was measured at 282 nm.

LC‐MS/MS analysis was conducted using a gradient program as follows: 0–1 min, 10% B; 1–10 min, 10–95% B; 12.3‐13 min, 95% B; 13–15 min, 95%–10% B. Other conditions were consistent with the LC‐MS/MS method for analyzing the accumulation of BIAs in different tissues of *M. dauricum*.

### Selection and Functional Identification of CYP80 and CYP719 Genes

The CYP450 genes were identified in various plant species, including *M. dauricum*, *P. somniferum*, *P* *apaver* *setigerum*, *P. rhoeas*, *E. californica*, *M. cordata*, *C. chinensis*, *A. caerulea*, *E. pubescens*, *K. uniflora*, *N. nucifera*, *V. vinifera* and *C. canephora*, using reported CYP80s and CYP719s as seeds via BLASTp with an E‐value of 1e^−10^. We downloaded the BsCYP80A (AAC48987.1) from *B. stolonifera*, CjCYP80B2 (AAC61839.1), CjCYP80G2 (BAF80448.1) and CjCYP719A1 (BAB68769.1) from *C. japonica*, NnCYP80Q1 (NNU_21 372) and NnCYP80Q2 (NNU_21 373) from *N. nucifera*, PsCYP719A21 (I3QBP4.1) and PsCYP719B1 (B1NF18.1) from *P. somniferum*, TfCYP719A (AAU20771.1) from *T. flavum*, AmCYP719A13 (B1NF19.1) and AmCYP719A14 (B1NF20.1) from *Argemone mexicana*, EcCYP719A2 (ACO90219.1), EcCYP719A3 (BAD98249.1) and EcCYP719A5 (B5UAQ8.1) from *E. californica* for BLASTP analysis. We followed the CYP nomenclature system^[^ ^84^ ^]^ and retained those BLAST results with >40% amino acid sequence identity to relevant CYP80 or CYP719 genes. Then, the annotated CYP80 and CYP719 genes from candidate species were used to respectively construct the ML phylogenetic tree using MAFFT (v6.240) and IQ‐TREE (v2.0.3) analysis.

The candidate P450 genes were cloned into the pESC‐His vector and transformed into the *Saccharomyces cerevisiae* WAT11 strain, which is engineered for heterologous expression of CYP450 reductase from *Arabidopsis thaliana* (AtCPR). The positive transgenic yeast colonies were selected and cultivated in synthetic complete (SC) medium lacking histidine and containing 2% (w/v) glucose, whereas the empty vector served as the negative control. The yeast culture was grown at 30 °C with shaking at 200 rpm for 24 h, and then was expanded to 500 mL of YPDA medium containing 1% glucose and 2% galactose until the glucose was consumed completely. After 24 h of induction, a 15 mL culture of yeast was centrifuged at 1000 × *g* for 5 min. The yeast cells were then lysed with 0.3 m NaOH at 4 °C for 30 min and then centrifuged again at 1000 × *g* for 5 min. The sediment was collected and resuspended in 80 µL PBS and 20 µL of 5 × loading buffer. The resuspended mixture was then boiled for 10 min for standard western blot detection. To determine whether the target protein was expressed, Anti‐C‐Myc (Abmart, China) and HRP goat anti‐mouse IgG antibodies (ABclonal, China) were used as the primary and secondary antibodies, respectively. The remanent yeast cells were harvested by centrifugation at 5000 *× g* for 6 min, washed successively with 40 mL of TES buffer (consisting of D‐sorbitol, Tris‐HCl, and EDTA) and 30 mL of TES‐M buffer (30 mm β‐mercaptoethanol in TES buffer), and resuspended in 25 mL of extract buffer (bovine serum albumin, PMSF, and β‐mercaptoethanol in TES buffer). The yeast cells were then lysed with an ultra‐high pressure cell crusher, and the resulting supernatant was collected by sequential centrifugation at 5000 *× g* and 10 000 *× g* for 10 min each at 4 °C, followed by centrifugation at 120 000 *× g* for 90 min at 4 °C. The pellet containing microsomes was resuspended in 1 mL of TEG‐M buffer (20% [v/v] glycerol in TES), and the recombinant protein was aliquoted and stored at −80 °C.

The enzymatic assays were performed at 30 °C for 2 h in a reaction system containing 200 µL of microsomes, 0.5 mm of NADPH, and 5 µg mL^−1^ of substrate. The reaction was quenched by adding 200 µL of methanol, and the resulting supernatant was dried at 60 °C, resuspended in 200 µL of methanol, and the filtered aliquot was later analyzed by LC‐MS/MS.

LC‐MS/MS analysis was performed on an UPLC‐QTOF‐MS system (Angilent 1290 Infinity II LC‐6430 QTOF) equipped with an ACQUITY UPLC HSS‐T3 C18 column (1.8 µm, 2.1 mm × 100 mm). The mobile phase consisted of 0.1% formic acid in water (A) and acetonitrile (B), and the linear gradient elution program was as follows: 0–2 min, 5% B; 2–10 min, 5%–18% B; 10–15 min, 18%–95% B; 15–17 min, 95% B (flow rate, 0.3 mL min^−1^; column temperature, 40 °C). The mass spectrometer equipped with  electrospray ionization (ESI) source operating in positive ion mode, with a mass‐to‐charge ratio (*m/z*) range of 100–800. The gas temperature was maintained at 350 °C, the gas flow rate was 8 L min^−1^, and the fragmentor was set to 120 V.

### Transient Expression of Candidate CYP80 and CYP719 Proteins in Nicotiana benthamiana

The CYP80 and CYP719 genes were cloned into the pEAQ‐eGFP vector and transformed into *Agrobacterium tumefacient* (GV3101). The positive transgenic yeast colonies were selected and resuspended in 1 mL of LB medium, then centrifuged at 5000 × *g* for 5 min. The pellets were then resuspended in 10 mm MES buffer, 10 mm MgCl_2_, and 150 µm acetosyringone, and incubated at 28 °C in the dark for 1 h. *Agrobacterium* suspension with candidate gene (OD_600_ = 0.3‐0.6 for each strain) was infiltrated into the abaxial side of 5‐6‐week‐old *N. benthamiana* leaves (on a 14 h light‐cycle) using a 1 mL syringe until the entire leaf was infiltrated. The injected *N. benthamiana* plants were then kept in the dark for 1 day, followed by 2 days of light exposure (on a 14 h light‐cycle). Next, 50 µm of substrates in 0.1% DMSO in water were respectively infiltrated into the abaxial side of previously *Agrobacterium*‐infiltrated leaves. Leaves were harvested 1 day later, flash frozen, and stored at −80 °C for later processing. Each treatment consisted of 3 or 4 leaves, and the biological replicates consisted of 3 different plants. Two negative controls were employed, with one group receiving an injection of an empty vector and the corresponding substrates into *N. benthamiana*, and the other group receiving an injection of the corresponding *Agrobacterium* suspensions with candidate genes without substrates.

Accurately weighed 5 mg of lyophilized tissue powders were poured into a 15 mL centrifuge tube. 10 mL of methanol was added to the tube and then dissolved by ultrasonication for 20 min. The samples were then spun‐dried for 12 h and reconstituted with 400 µL of methanol. The samples were again dissolved by ultrasonication for 20 min and then centrifuged at 8000 *× g* for 30 min. After filtration, 0.5 mg mL^−1^ tissue solutions were prepared for LC‐MS/MS analysis. The conditions were consistent with the LC‐MS/MS method for functional identification of CYP80 and CYP719.

### Statistical Analyses

All experiments were independently carried out at least three times. One way analysis of variance (ANOVA) was used, followed by Newman‐Keuls post‐hoc test, to analyze the variability in the quantity of ancestral genes mapped within each syntenic block across the Ranunculales lineage. Statistical analysis was carried out using R software.

## Conflict of Interest

The authors declare no conflict of interest.

## Author Contributions

Z.A., R.G., S.C., and Y.T. contributed equally to this work. Z.X., W.S. and S.C. designed and coordinated the study. B.Z. and L.T. sampled the plant materials. Z.A. and S.C. performed the bioinformatic analysis. R.G., Z.A., Q.L., and Y.T. performed the experiments and analyzed the data. W.Z. and L.K. financed a part of the research data. Z.X. and H.F. directed the experimental analysis. L.H., T.X., H.Y., Y.W., W.S., X.H., C.L., and J.S. helped perform the research. Z.X., Z.A., S.C., S.C., R.G., W.S., and H.F. wrote and revised the manuscript.

## Supporting information

Supporting Information

Supporting Information

## Data Availability

The data that support the findings of this study are openly available in Figshare at https://doi.org/10.6084/m9.figshare.23509239, reference number 23509239.
