# Supplementary material for: Lineage‐Specific CYP80 Expansion and Benzylisoquinoline Alkaloid Diversity in Early‐Diverging Eudicots
Source: Adv Sci (Weinh). 2024 Mar 13;11(19):2309990. doi: 10.1002/advs.202309990 (PMC11109638; doi:10.1002/advs.202309990)
Supplement: Supplementary file 1 — Supporting Information [file ADVS-11-2309990-s002.pdf]

ADVANCED SCIENCE
Open Access

Supporting Information

for *Adv. Sci.*, DOI 10.1002/advs.202309990

Lineage-Specific CYP80 Expansion and Benzylisoquinoline Alkaloid Diversity in Early-Diverging Eudicots

Zhoujie An, Ranran Gao, Shanshan Chen, Ya Tian, Qi Li, Lixia Tian, Wanran Zhang, Lingzhe Kong, Baojiang Zheng, Lijun Hao, Tianyi Xin, Hui Yao, Yu Wang, Wei Song, Xin Hua, Chengwei Liu, Jingyuan Song, Huahao Fan*, Wei Sun*, Shilin Chen* and Zhichao Xu**

Supporting Information

Lineage-specific CYP80 expansion and benzylisoquinoline alkaloid diversity in early-diverging eudicots

Zhoujie An, Ranran Gao, Shanshan Chen, Ya Tian, Qi Li, Lixia Tian, Wanran Zhang, Lingzhe Kong, Baojiang Zheng, Lijun Hao, Tianyi Xin, Hui Yao, Yu Wang, Wei Song, Xin Hua, Chengwei Liu, Jingyuan Song, Huahao Fan*, Wei Sun*, Shilin Chen*, Zhichao Xu**

Supporting Information

Lineage-specific CYP80 Expansion and Benzylisoquinoline Alkaloid Diversity in Early-diverging Eudicots

Zhoujie An[1,2#], Ranran Gao[3#*], Shanshan Chen[3#], Ya Tian[1,2#], Qi Li[1,2], Lixia Tian[4], Wanran Zhang[1,2], Lingzhe Kong[1,2], Baojiang Zheng[1,2], Lijun Hao[5], Tianyi Xin[5], Hui Yao[5], Yu Wang[5], Wei Song[1,2], Xin Hua[1,2], Chengwei Liu[1,2], Jingyuan Song[5], Huahao Fan[6*], Wei Sun[3*], Shilin Chen[3,7*], Zhichao Xu[1,2*]

[1] Key Laboratory of Saline-alkali Vegetation Ecology Restoration (Northeast Forestry University), Ministry of Education, Harbin 150040, China

[2] College of Life Science, Northeast Forestry University, Harbin, 150040, China

[3] Key Laboratory of Beijing for Identification and Safety Evaluation of Chinese Medicine, Institute of Chinese Materia Medica, China Academy of Chinese Medical Sciences, Beijing 100700, China

[4] School of Pharmaceutical Sciences, Guizhou University, Guiyang, 550025, China

[5] Key Lab of Chinese Medicine Resources Conservation, State Administration of Traditional Chinese Medicine of the People's Republic of China, Institute of Medicinal Plant Development, Chinese Academy of Medical Sciences & Peking Union Medical College, Beijing 100193, China

[6] College of Life Science and Technology, Beijing University of Chemical Technology; Beijing, 100029, China.

[7] Institute of Herbgenomics, Chengdu University of Traditional Chinese Medicine, Chengdu 611137, China

[#]These authors contributed equally to this article.

[*]Email: Zhichao Xu (zcxu@nefu.edu.cn); Ranran Gao (rrgao1991@icmm.ac.cn); Huahao Fan (fanhuahao@mail.buct.edu.cn); Wei Sun (wsun@icmm.ac.cn); Shilin Chen (slchen@icmm.ac.cn)

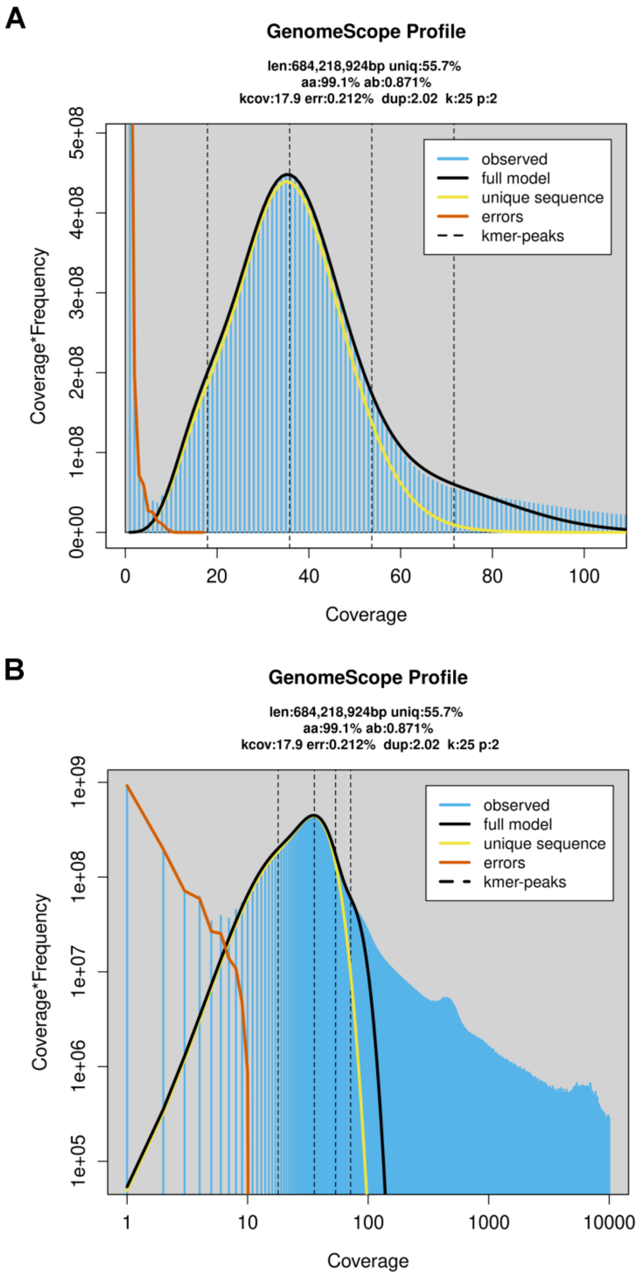

Fig. S1. *M. dauricum* genome size estimation. (**A-B**) Genome size estimation of *M. dauricum* genome using *k*-mer distribution (k=25).

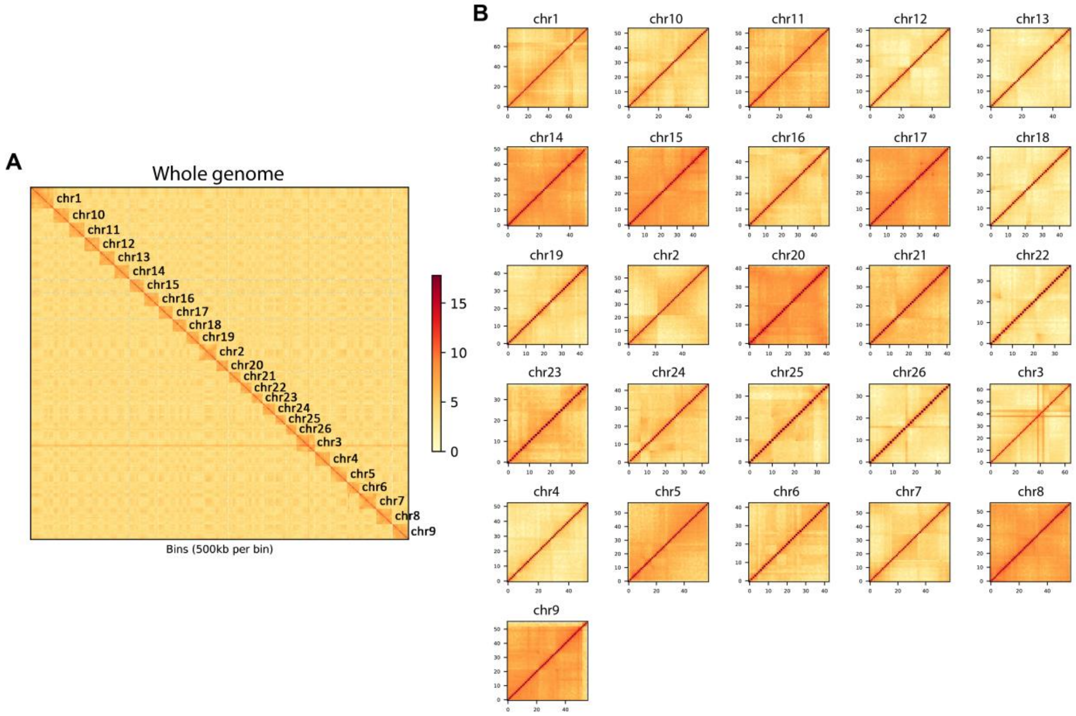

Fig. S2. Hi-C contact map representing the chromosome-scale genome of *M. dauricum*. The color intensity shows the count of Hi-C links at 500 kb size-windows on the chromosomes. The dots from light yellow to dark red show low to high probability of interactions.

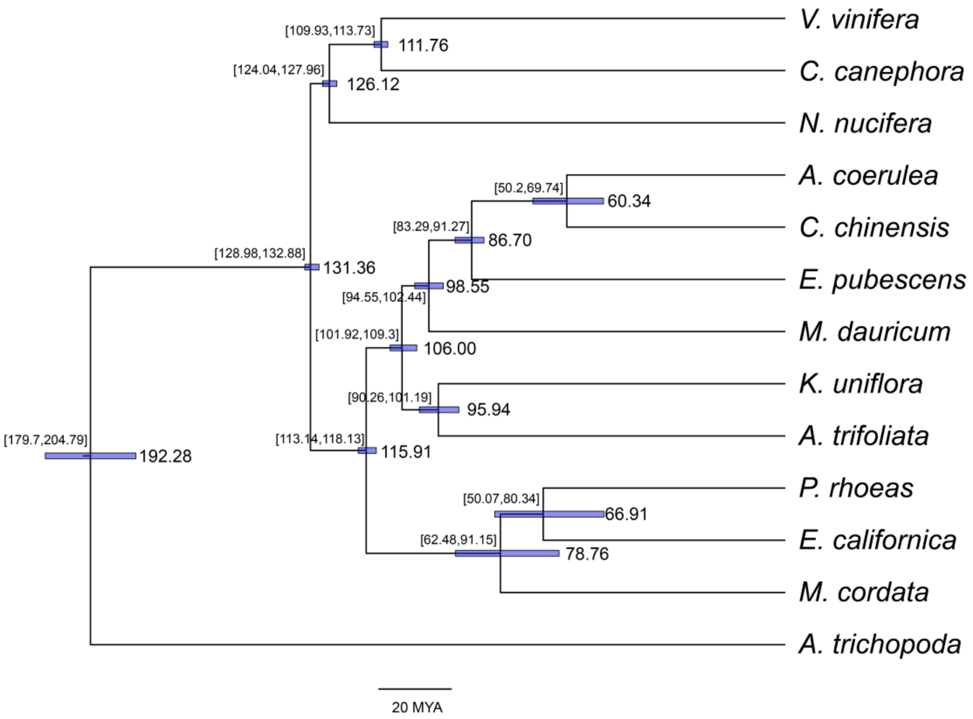

Fig. S3. Estimation of divergence times for candidate species using MCMCtree. Blue bars represent the 95% Confidence Interval (CI) for divergence times.

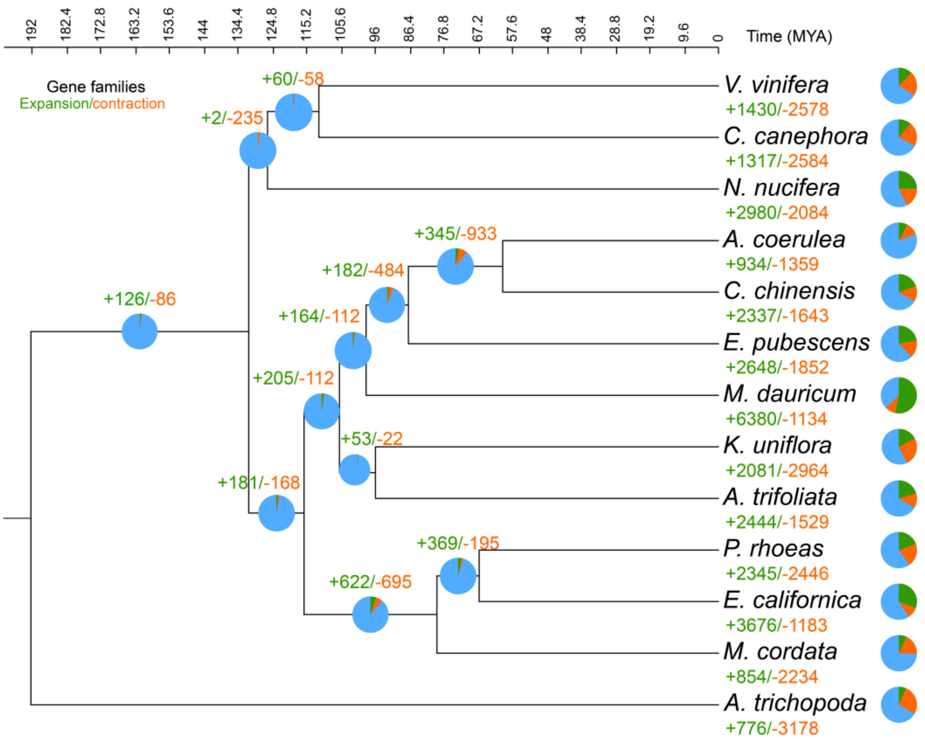

Fig. S4. Expansion and contraction of gene families in 13 angiosperm species using CAFE5.

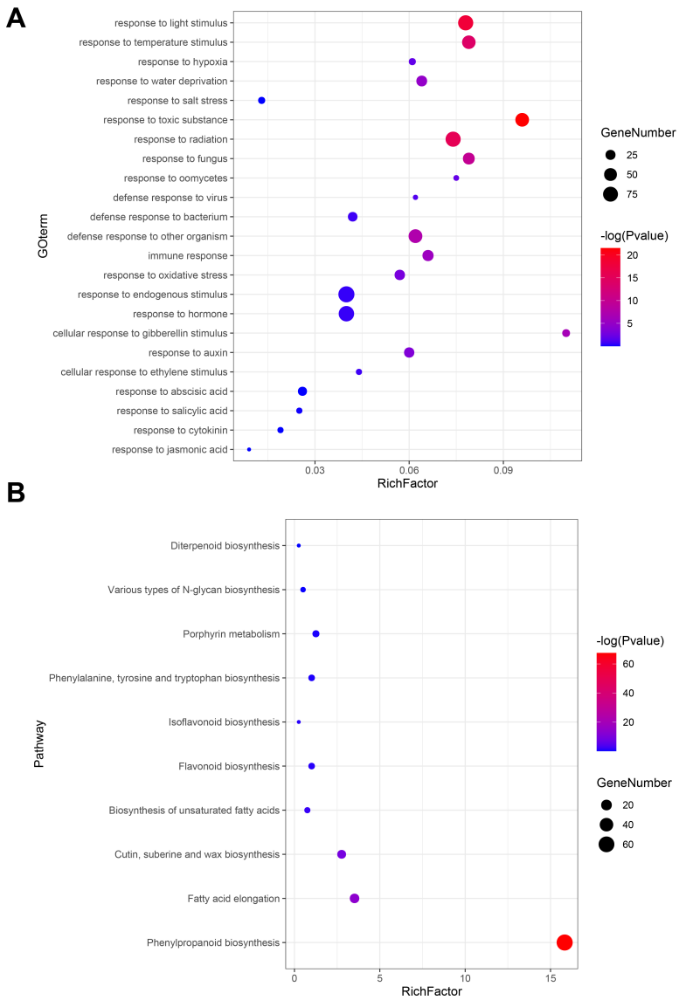

Fig. S5. Gene ontology (GO) (A) and KEGG pathway (B) enrichment analysis of expanded gene families in *M. dauricum*.

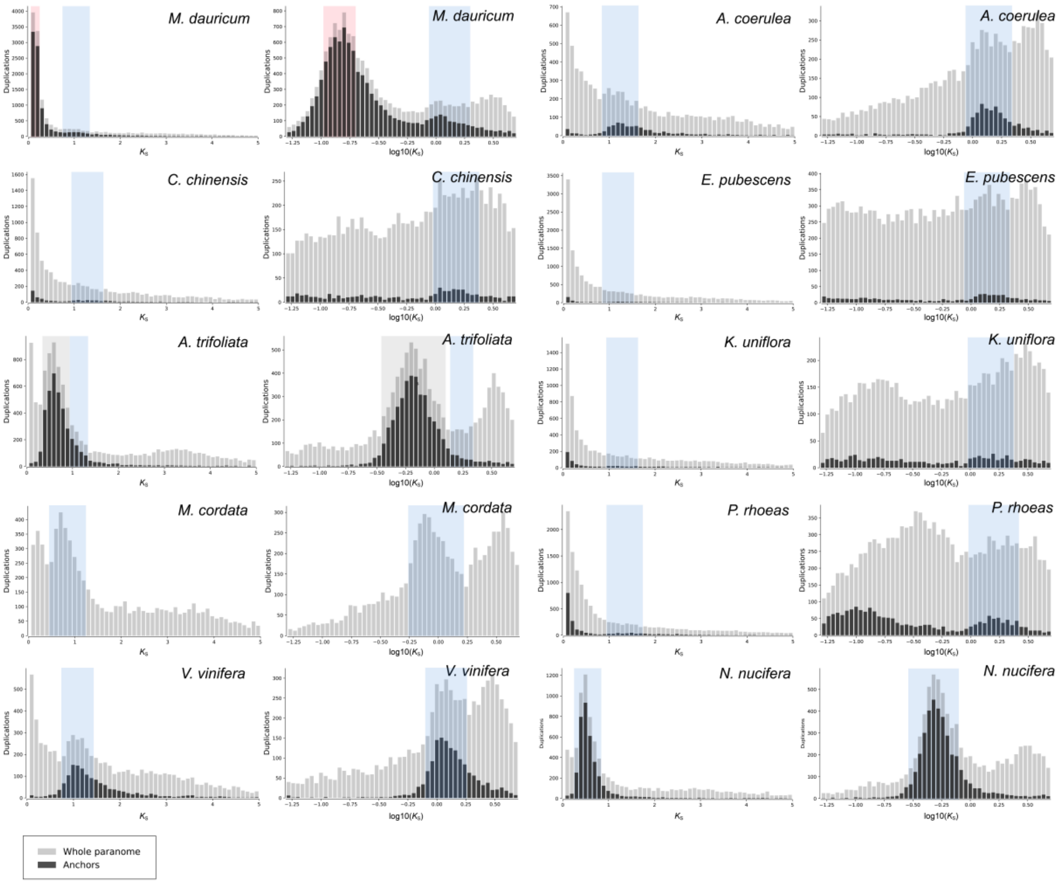

Fig. S6. *K*$_S$ distributions for paralogous pairs (grey bars) and anchored paralogs (black bars) identified within *M. dauricum*, other published Ranunculales species and *V. vinifera* genomes, respectively. The red, grey and blue rectangles highlight the *K*$_S$ peaks, represented the whole genome duplication events.

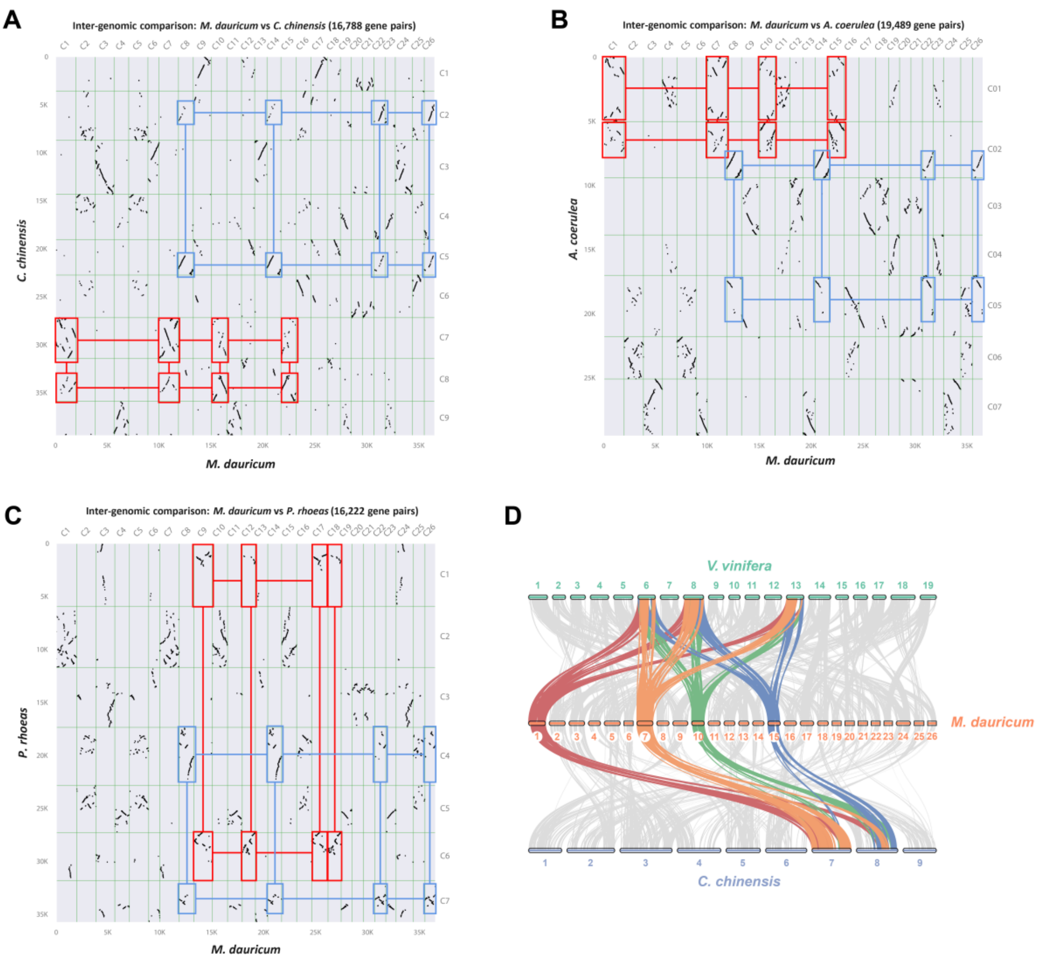

Fig. S7. Synteny analysis between the *M. dauricum* and other Ranunculales genomes. Dot plots showed the syntenic orthologs between *M. dauricum* and *C. chinensis* (**A**), between *M. dauricum* and *A. coerulea* (**B**) and between *M. dauricum* and *P. rhoea* (**C**). The red and blue boxes highlight several duplication regions. (**D**) Macrosynteny between *M. dauricum* and *V. vinifera*, and between *M. dauricum* and *C. chinensis* chromosomes. The red, orange, green, and blue lines represent the four-to-three correspondence of homologous regions between chromosome 1,7,10 and 15 of *M. dauricum* and *V. vinifera*, and the four-to-two correspondence of homologous regions between chromosome 1,7,10 and 15 of *M. dauricum* and *C. chinensis.*

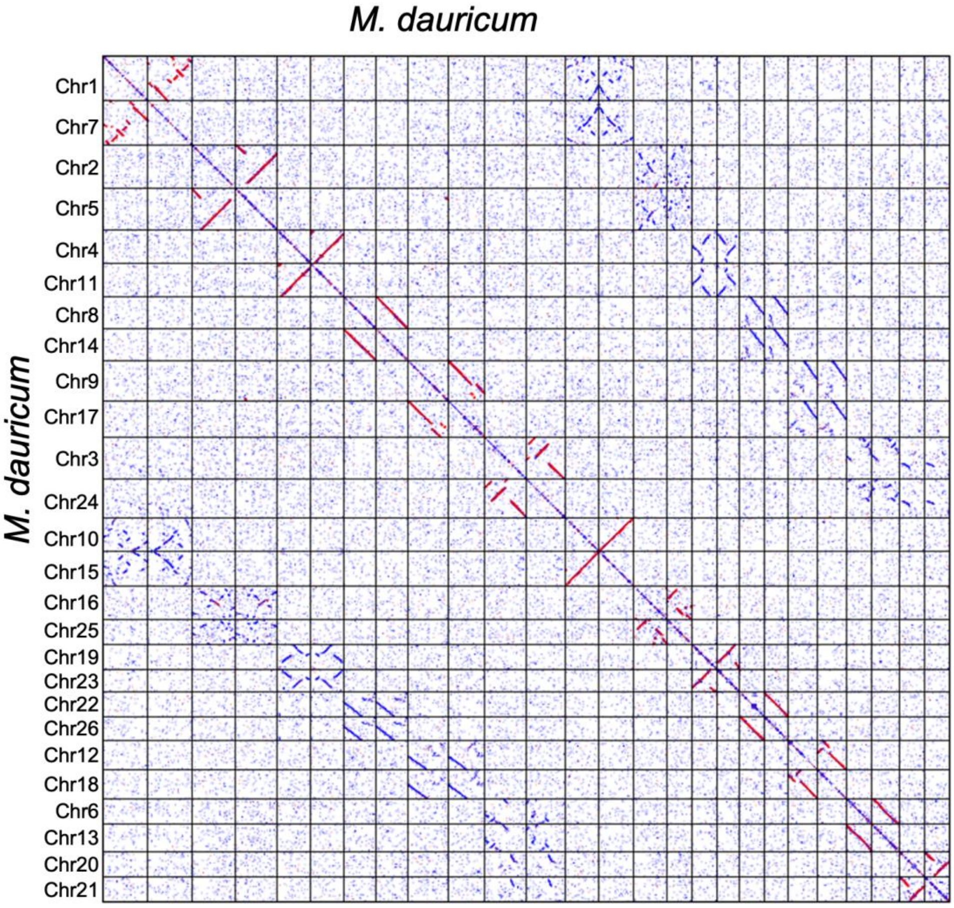

Fig. S8. Dot plot of genome collinearity in *M. dauricum* genome. The red dots (lines) represent the duplicated blocks after *Mdβ* event, and the blue dots indicated the duplicated segments of *Rα* event. The 26 *M. dauricum* chromosomes could be obviously grouped into 13 ancestral chromosomes of pre-*Mdβ* event.

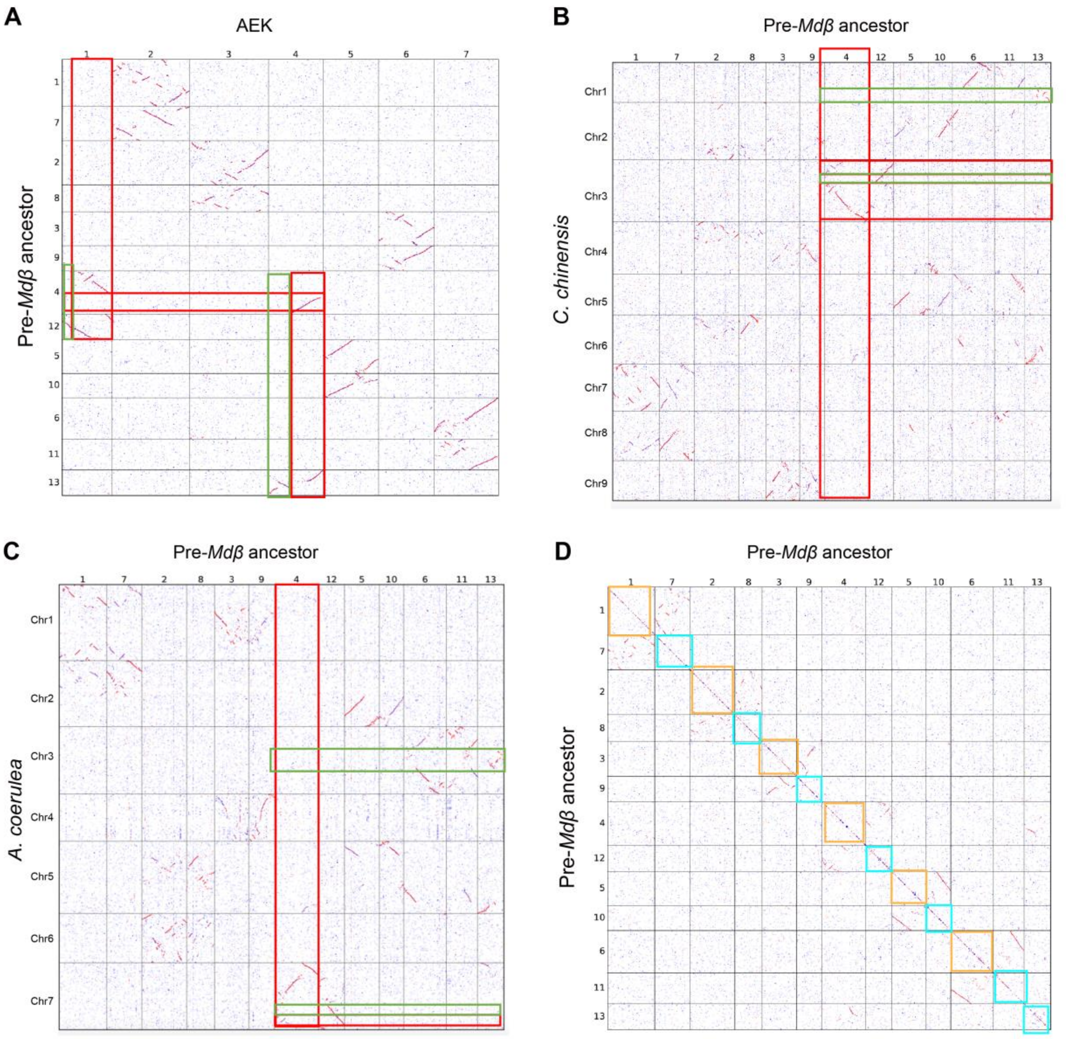

Fig. S9. The inferring of pre-*Rα* ancestors based on the genome collinearity among AEK, pre-*Mdβ* ancestor of *M. dauricum*, *C. chinensis*, and *A. coerulea*. (**A**), (**B**), and (**C**) represent the collinearity profiles of pre-*Mdβ* ancestor and AEK, *C. chinensis* and pre-*Mdβ* ancestor, and *A. coerulea* and pre-*Mdβ* ancestor, respectively. The Chr1, Chr2, Chr3, Chr5, Chr6, Chr7, Chr8, Chr9, Chr10, Chr11 of pre-*Mdβ* ancestor could be grouped into 5 ancestral chromosomes of pre-*Rα* ancestor. However, the parts of Chr4 of pre-*Mdβ* ancestor could be respectively mapped into Chr1 and Chr4 of AEK (red boxes), and the Chr12 and Chr13 of pre-*Mdβ* ancestor can be fully mapped to Chr1 and Chr4 of AEK respectively, which contains parts of Chr4 have not been mapped into AEK(green boxes), but there is not any collinearity relationship between Chr12 and Chr13, suggesting the Chr4 represent the fusion of Chr12 and Chr13 of pre-*Mdβ* ancestor and some genes were also lost during the fusion.

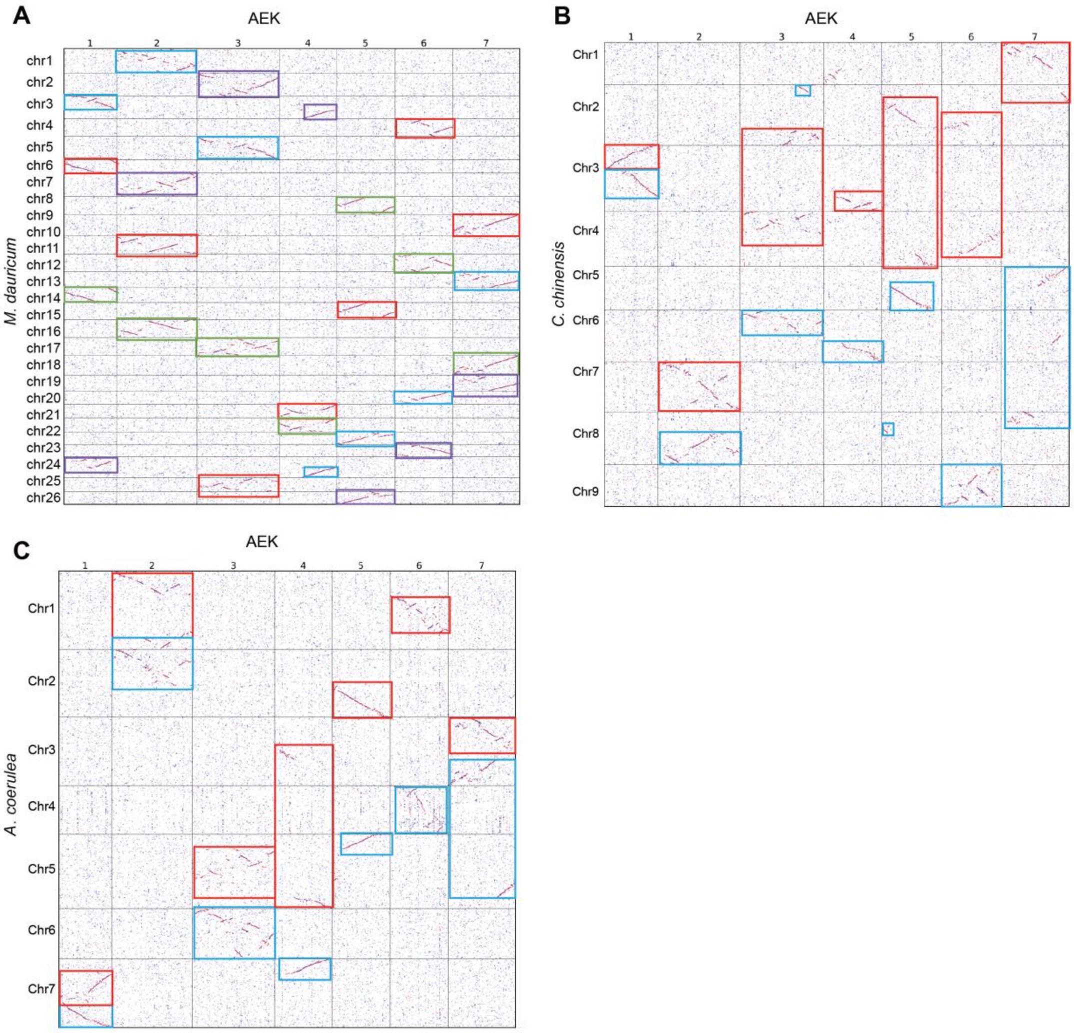

Fig. S10. The classification of the subgenomes of *M. dauricum* (A), *C. chinensis* (B), and *A. coerulea* (C) according to the collinearity relationship with AEK. The red and blue boxes in *M. dauricum*, *C. chinensis*, and *A. coerulea* represent subgenome A and B, respectively, and the green and purple boxes in *M. dauricum* represent the subgenome C and D, respectively.

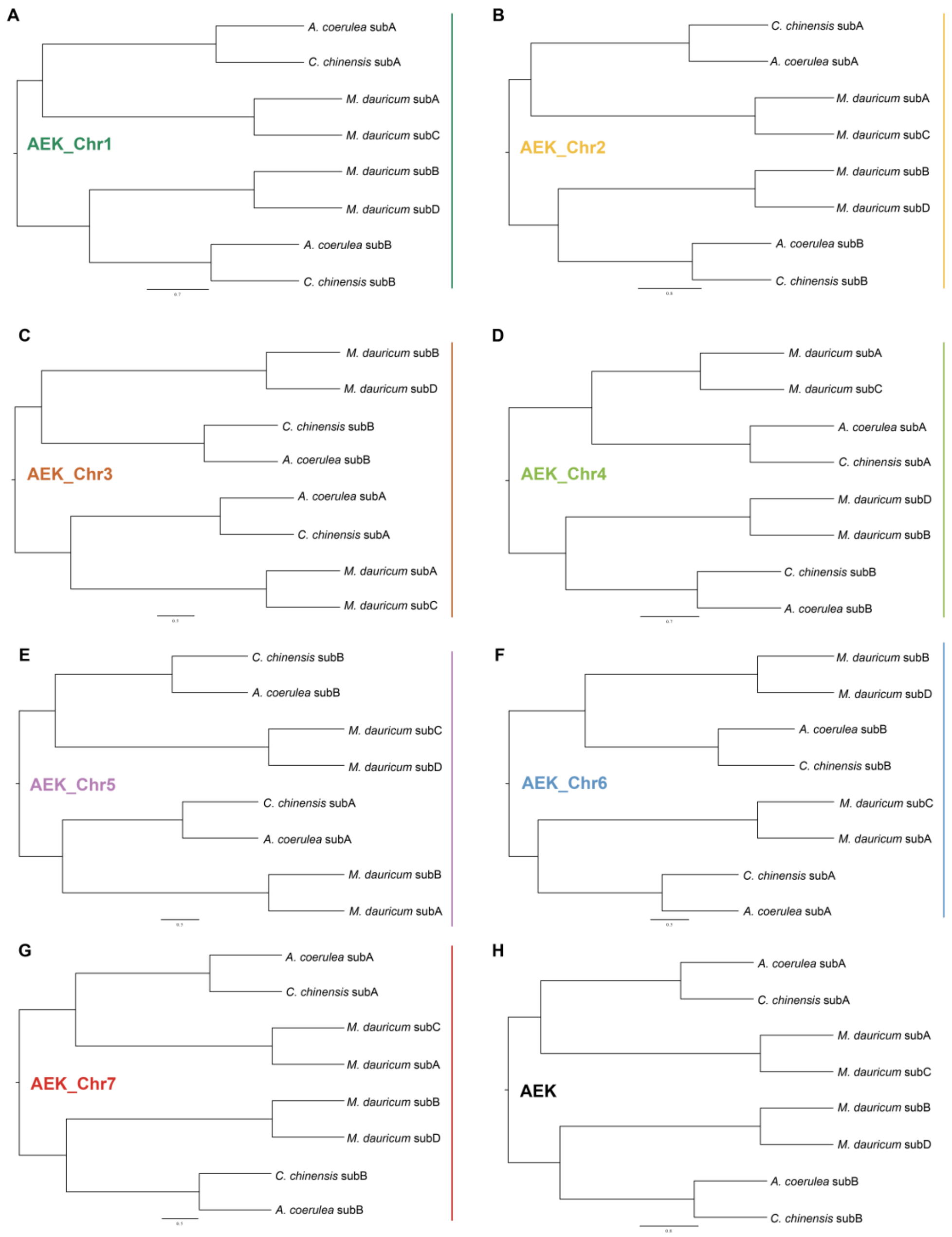

Fig. S11. Phylogenetic relationship of protochromosomes and subgenomes among *M. dauricum*, *C. chinensis* and *A. coerulea* using coalescent maximum-likelihood algorithm. (**A-G**) Phylogenetic trees were respectively constructed using the homologous genes from different chromosomes of subgenomes according to the collinearity mapping between candidate species and AEK chromosomes (Chr1-Chr7). (**H**) Phylogenetic tree using all the homologous genes from entire genomes according to the collinearity mapping between candidate species and AEK chromosomes (Chr1-Chr7). SubA and SubB represent the duplicated subgenomes after the shared *Rα* whole genome duplication event of *M. dauricum*, *C. chinensis* and *A. coerulea* genomes. SubC and SubD represent the duplicated subgenomes from SubA and SubB, respectively, after the *Mdβ* whole genome duplication event of *M. dauricum*. These results

showed that the subgenomes of *M. dauricum*, *C. chinensis* and *A. coerulea* clustered into two different phylogenetic clades (SubA and SubB), suggesting the Ranunculales-shared *Rα* whole genome duplication event is allopolyploidy.

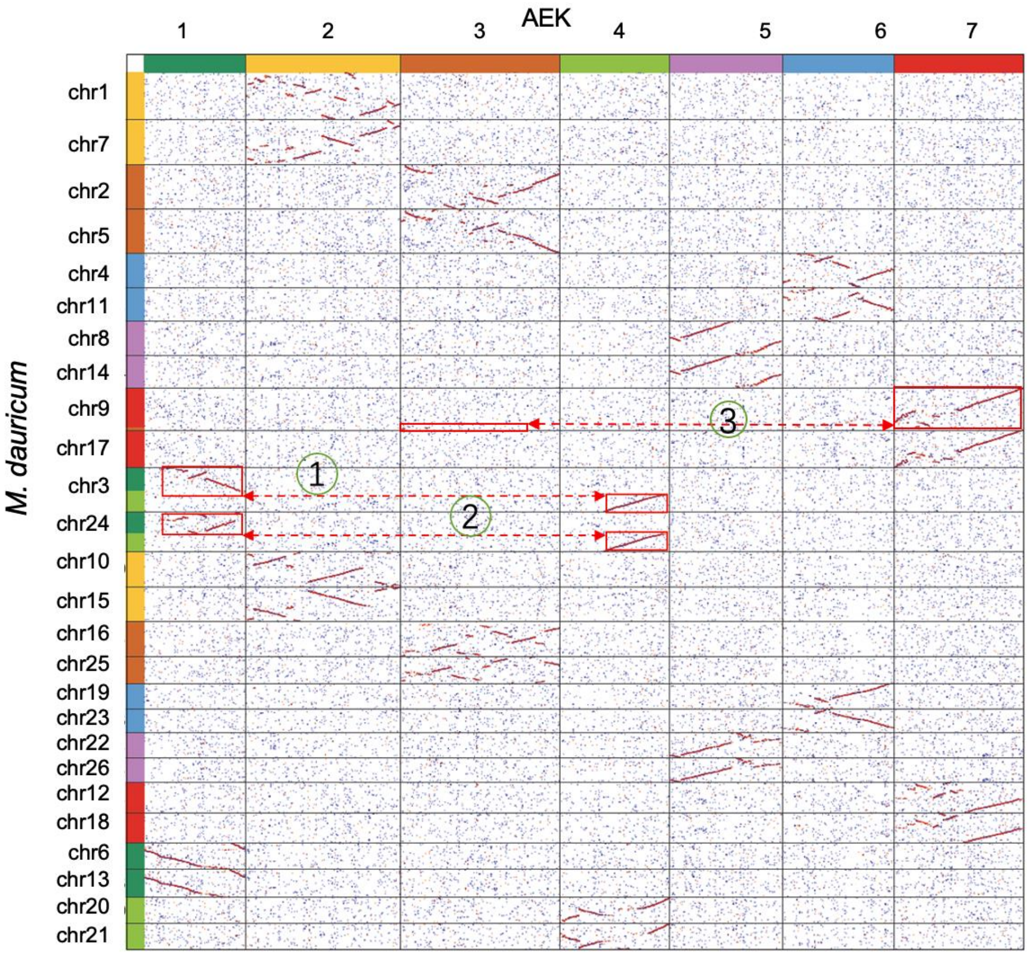

Fig. S12. Dotplot analyses of genome collinearity between *M. dauricum* and ancestral core-eudicot (AEK).

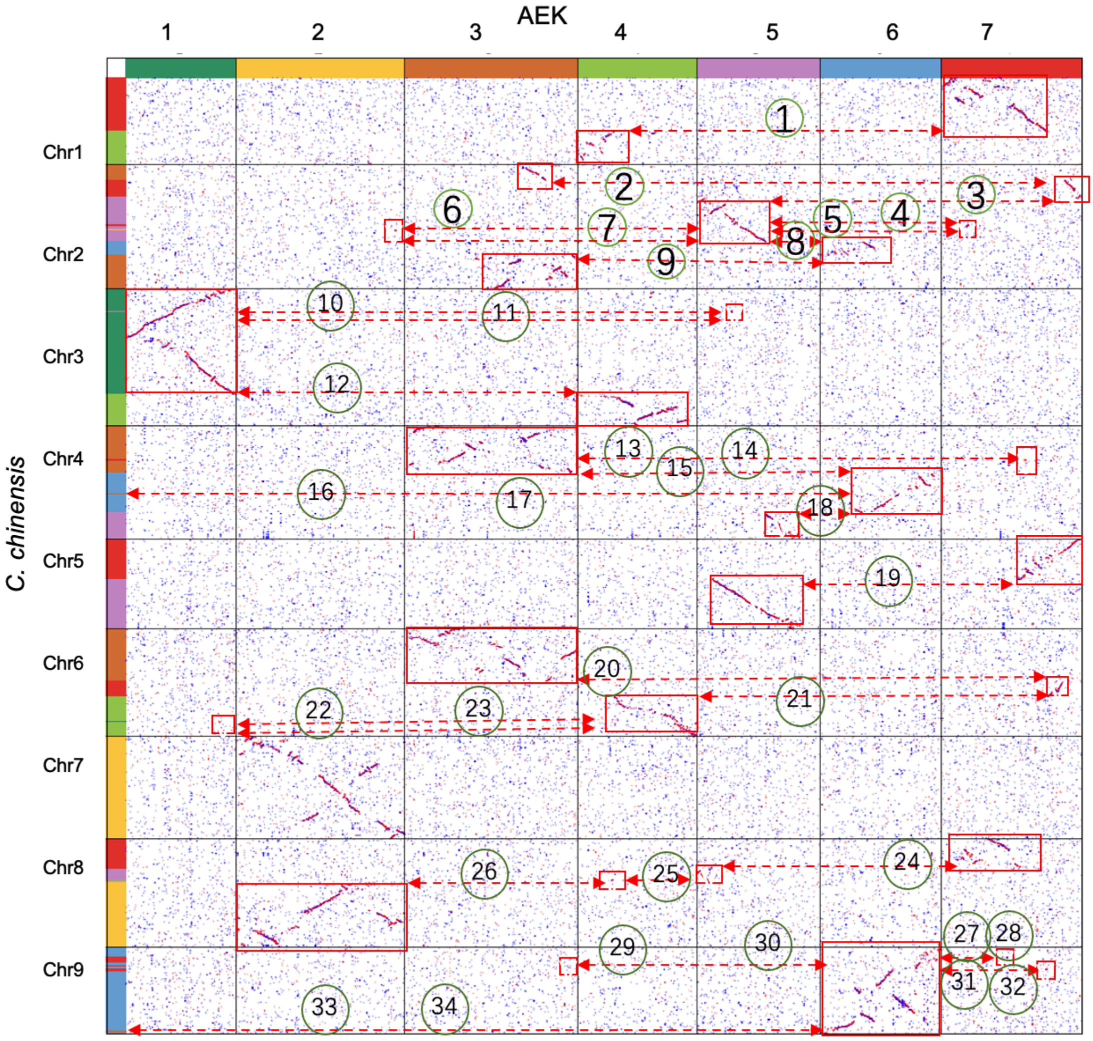

Fig. S13. Dotplot analyses of genome collinearity between *C. chinensis* and ancestral core-eudicot (AEK).

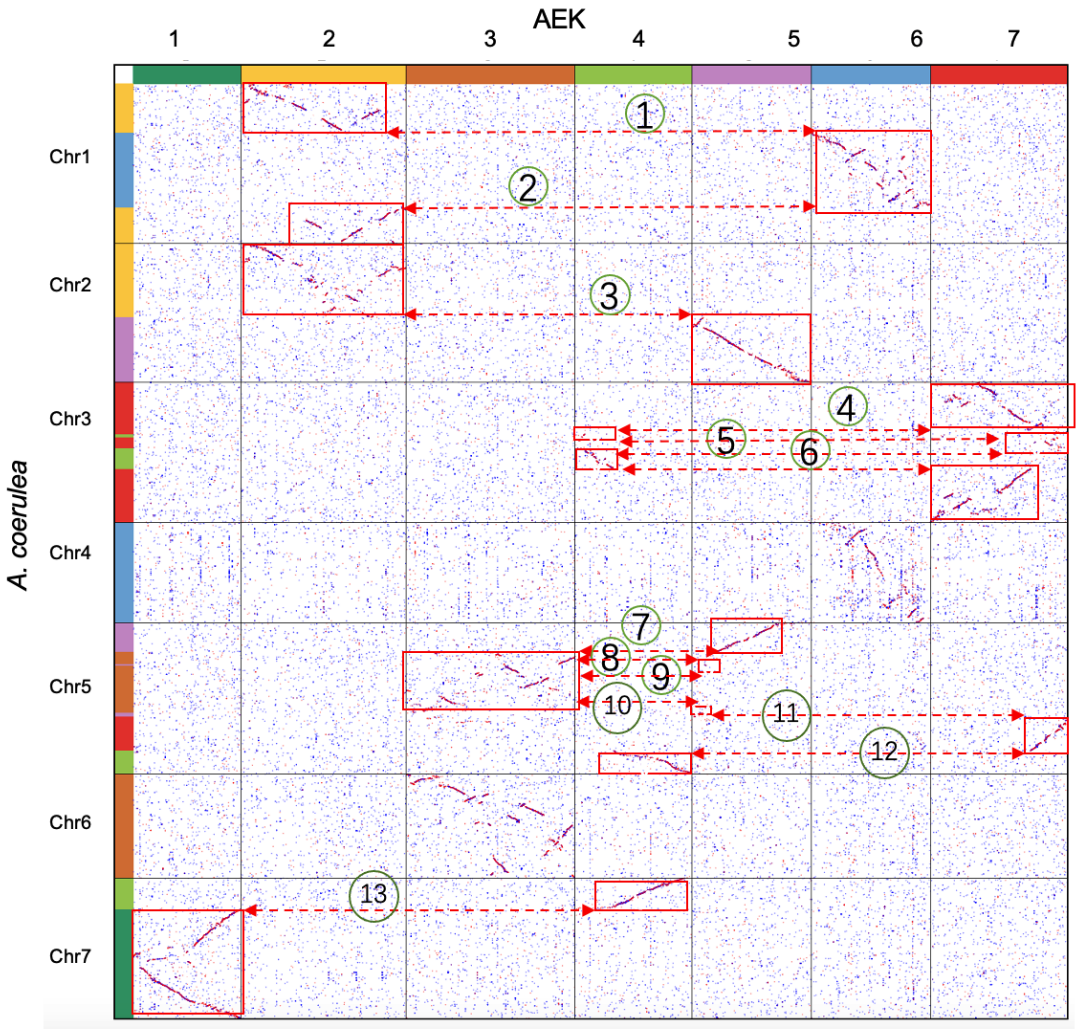

Fig. S14. Dotplot analyses of genome collinearity between *A. coerulea* and ancestral core-eudicot (AEK).

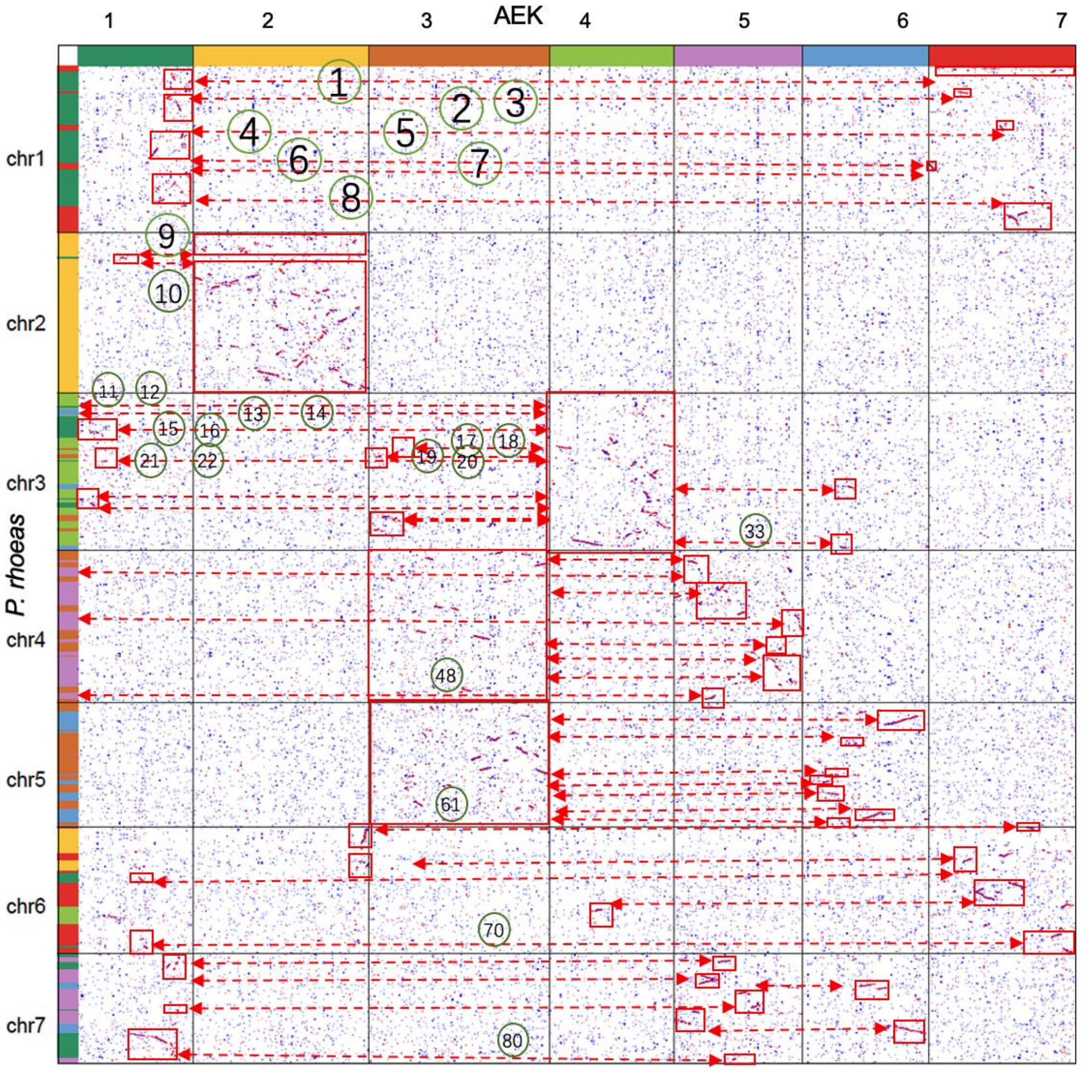

Fig. S15. Dotplot analyses of genome collinearity between *P. rhoeas* and ancestral core-eudicot (AEK).

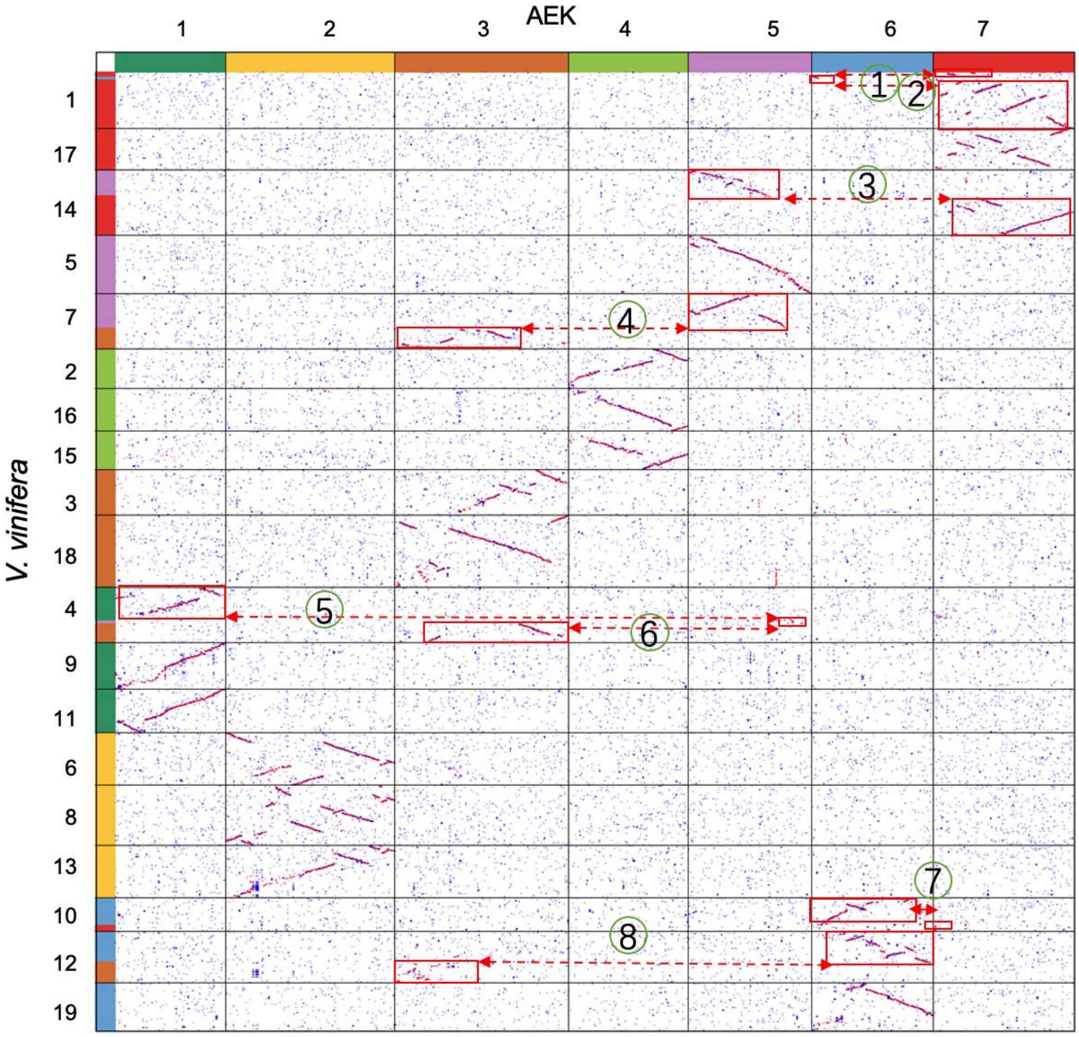

Fig. S16. Dotplot analyses of genome collinearity between grape and ancestral core-eudicot (AEK).

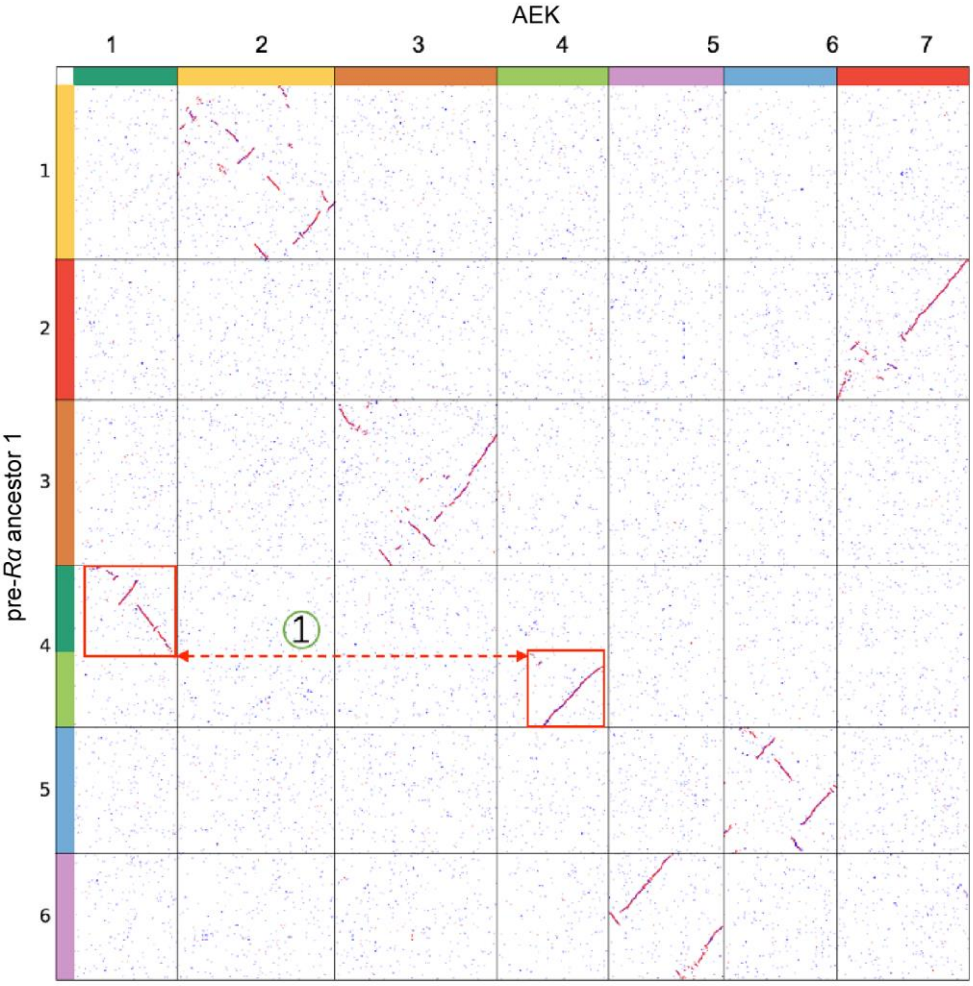

Fig. S17. Dotplot analyses of genome collinearity between pre-*Rα* ancestor 1 (putative pre-*Rα* karyotype) and ancestral core-eudicot (AEK). The number at the top represents the number of the AEK chromosome, with different colors to indicate different chromosomes. The numbers in the circles represent the number of fusions.

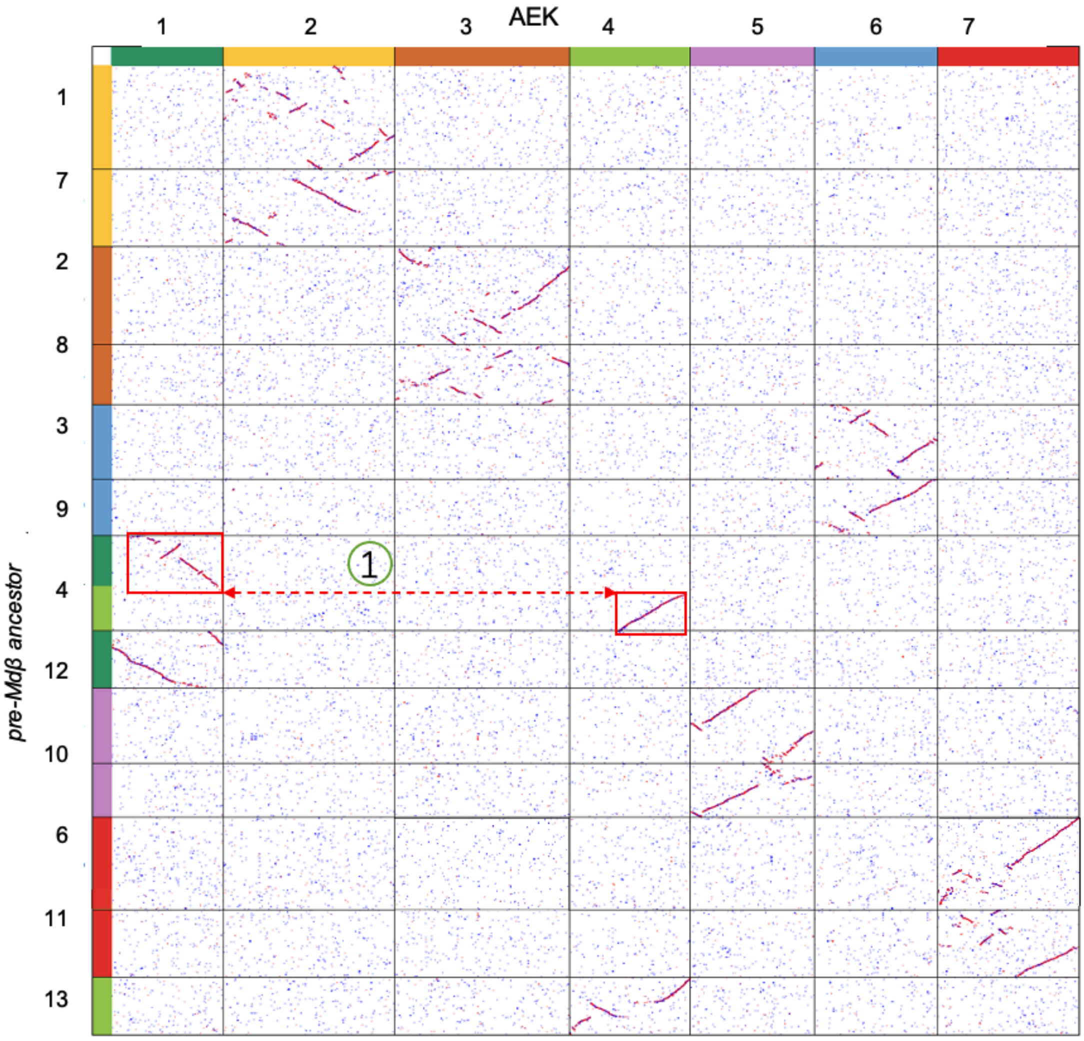

Fig. S18. Dotplot analyses of genome collinearity between pre-*Mdβ* ancestor (putative pre-*Mdβ*) and ancestral core-eudicot (AEK).

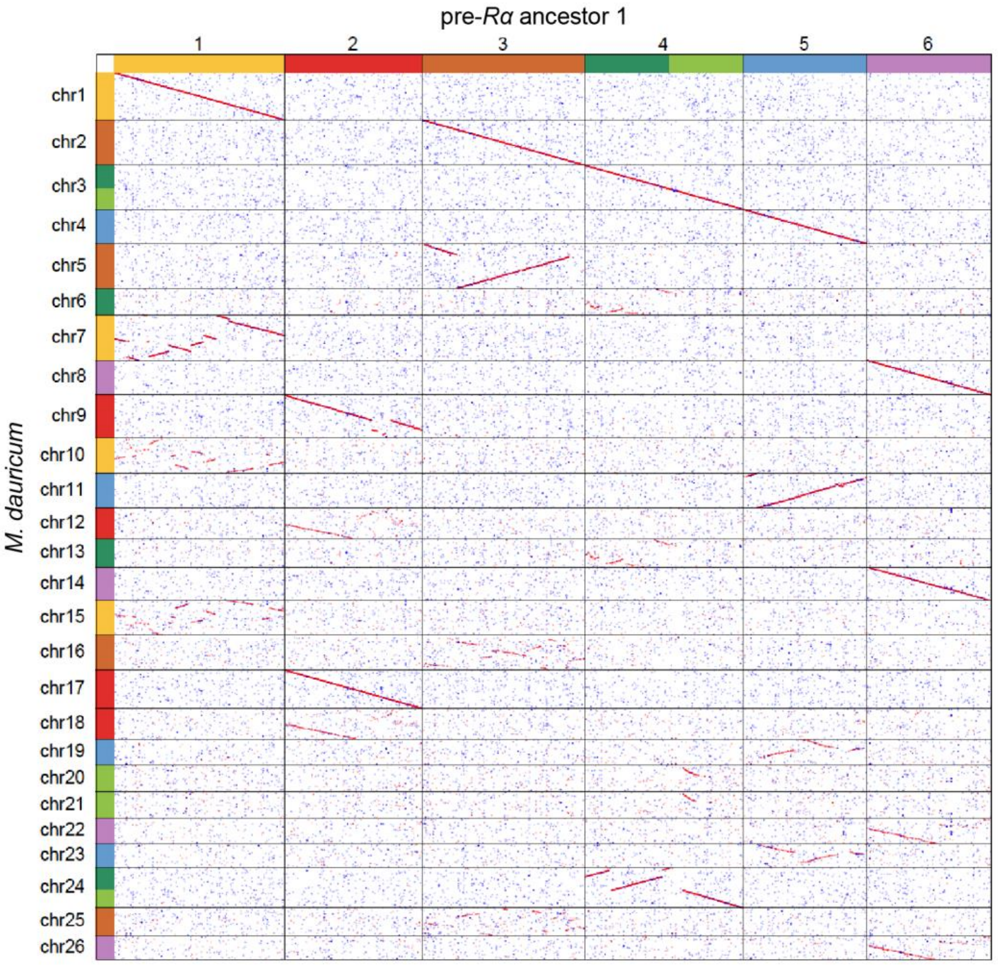

Fig. S19. Dotplot analyses of genome collinearity between *M. dauricum* and pre-*Rα* ancestor 1 (putative pre-*Rα* karyotype).

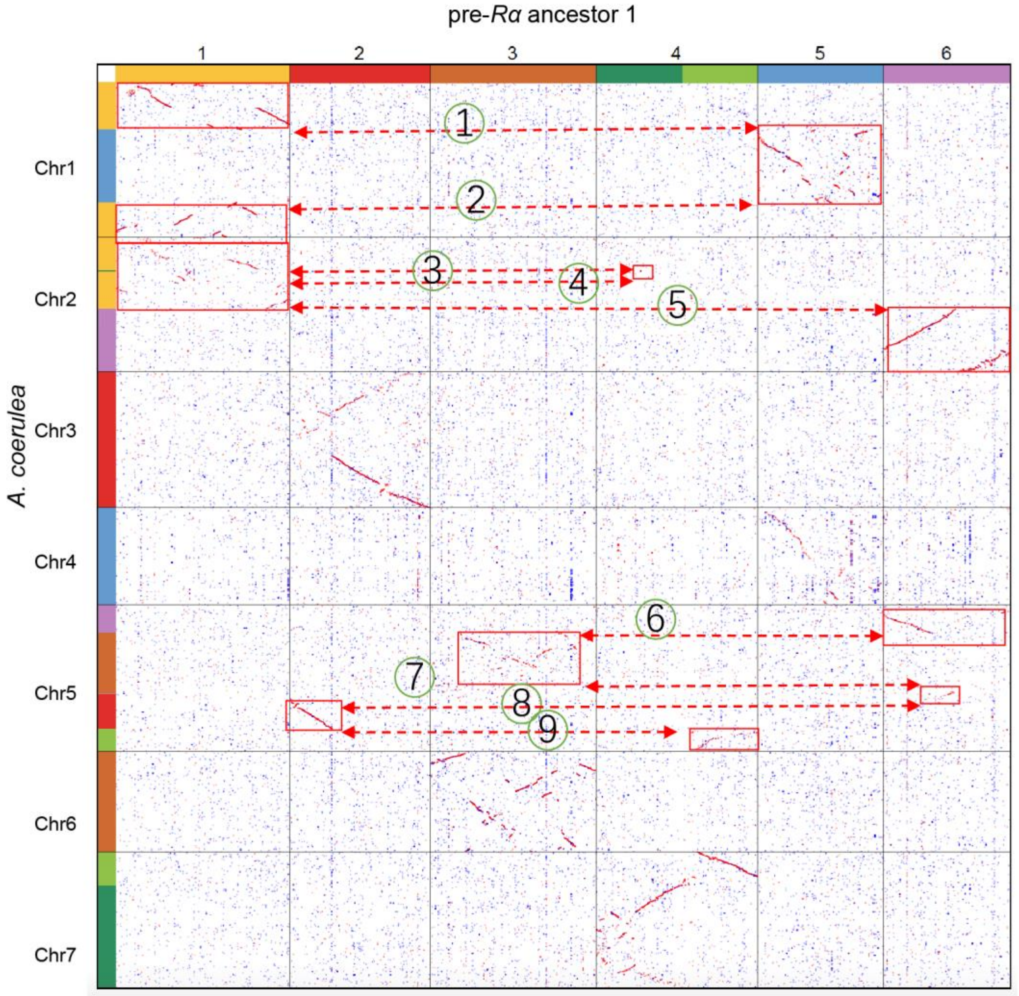

Fig. S20. Dotplot analyses of genome collinearity between *A. coerulea* and pre-*Rα* ancestor 1 (putative pre-*Rα* karyotype).

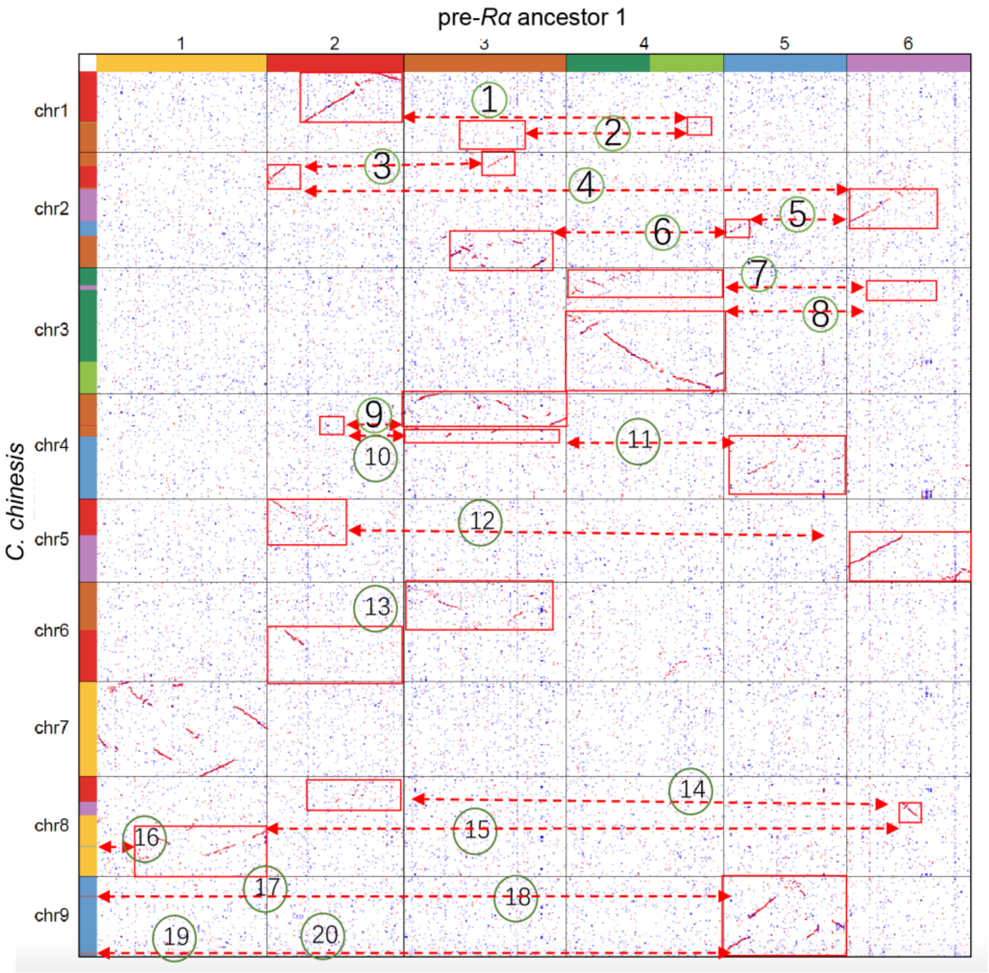

Fig. S21. Dotplot analyses of genome collinearity between *C. chinensis* and pre-*Rα* ancestor 1 (putative pre-*Rα* karyotype).

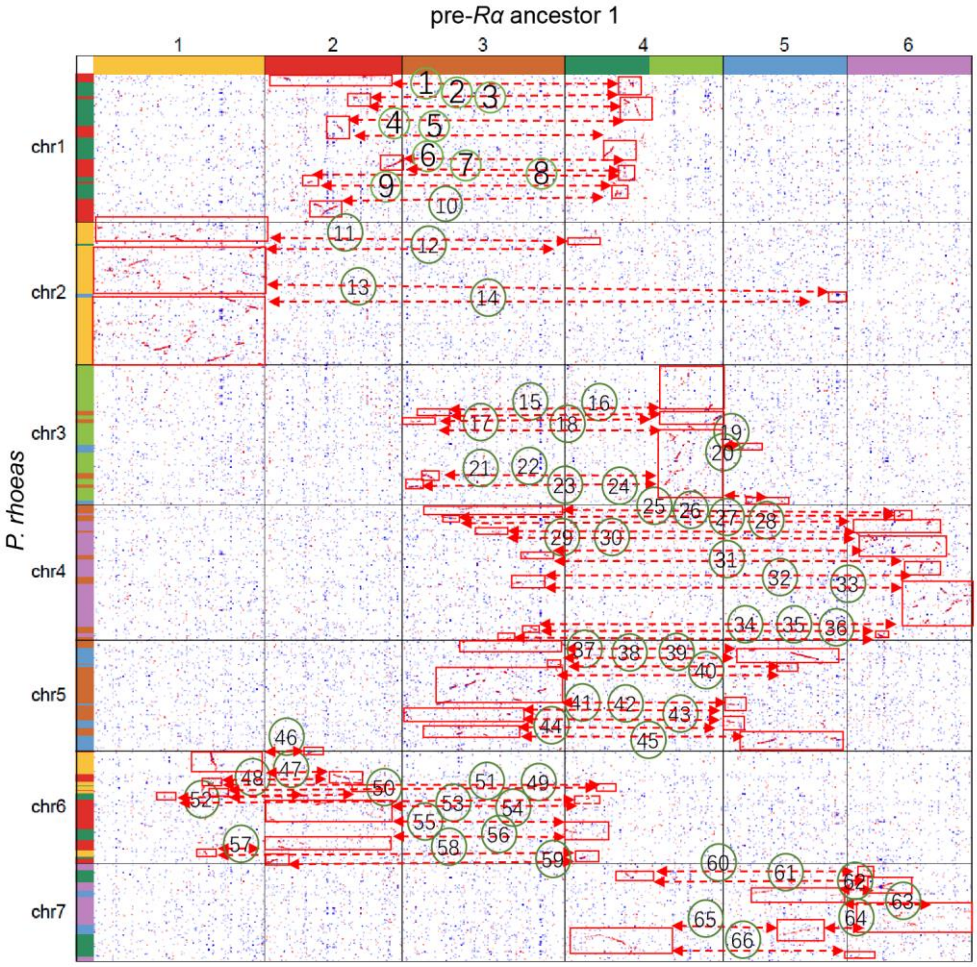

Fig. S22. Dotplot analyses of genome collinearity between *P. rhoeas* and pre-*Rα* ancestor 1 (putative pre-*Rα* karyotype).

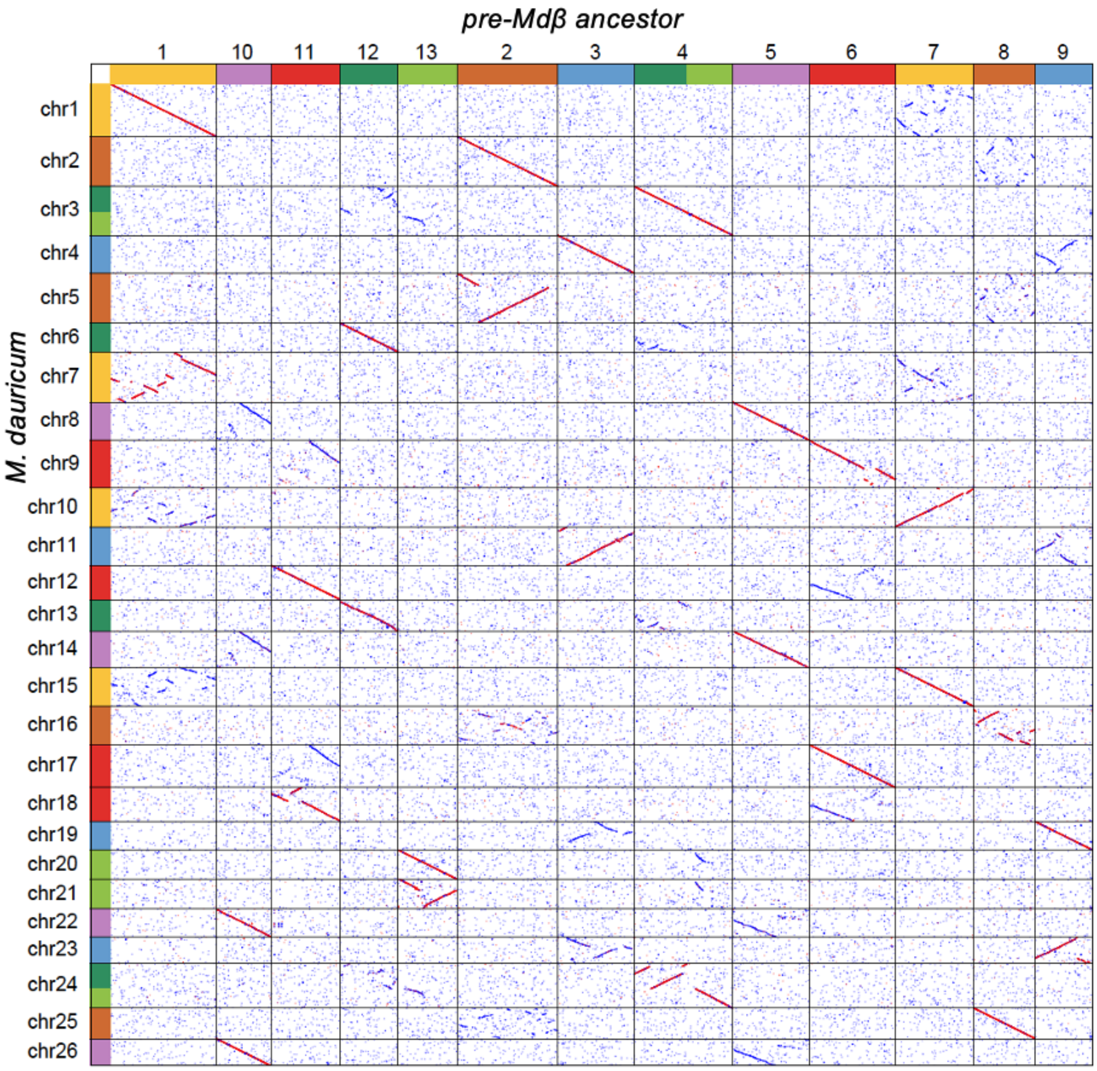

Fig. S23. Dotplot analyses of genome collinearity between *M. dauricum* and pre-*Mdβ* ancestor (putative pre-*Mdβ* karyotype).

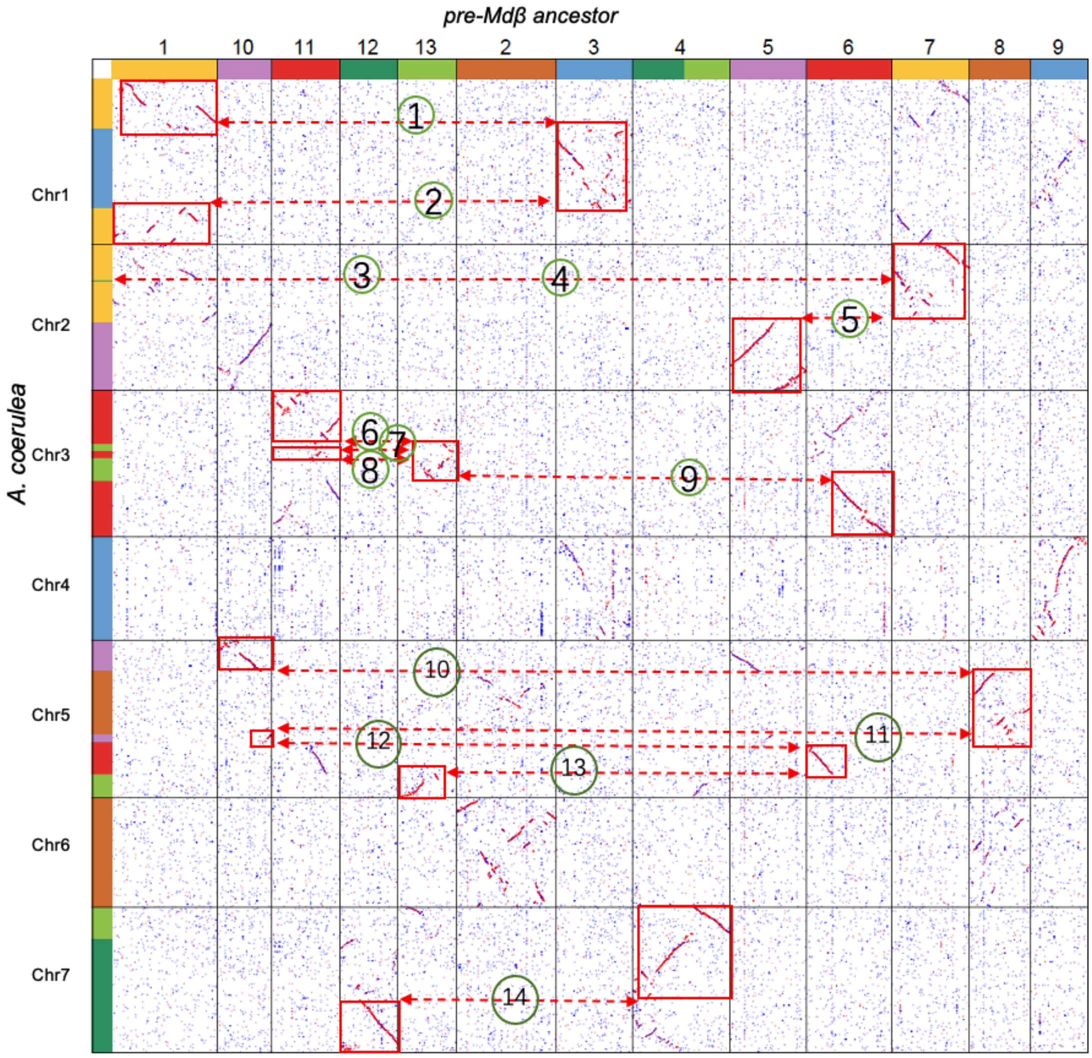

Fig. S24. Dotplot analyses of genome collinearity between *A. coerulea* and pre-*Mdβ* ancestor (putative pre-*Mdβ* karyotype).

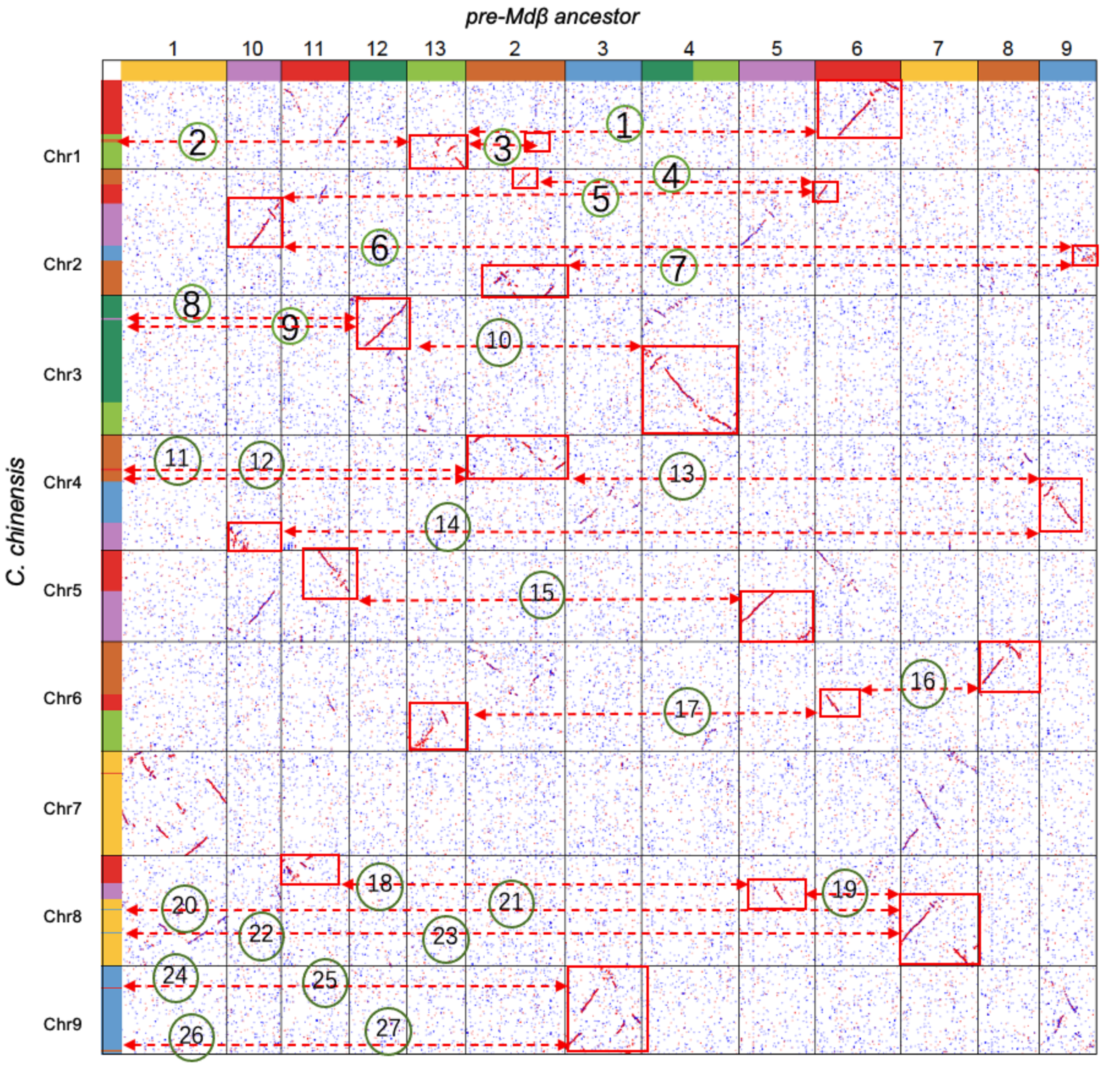

Fig. S25. Dotplot analyses of genome collinearity between *C. chinensis* and pre-*Mdβ* ancestor (putative pre-*Mdβ* karyotype).

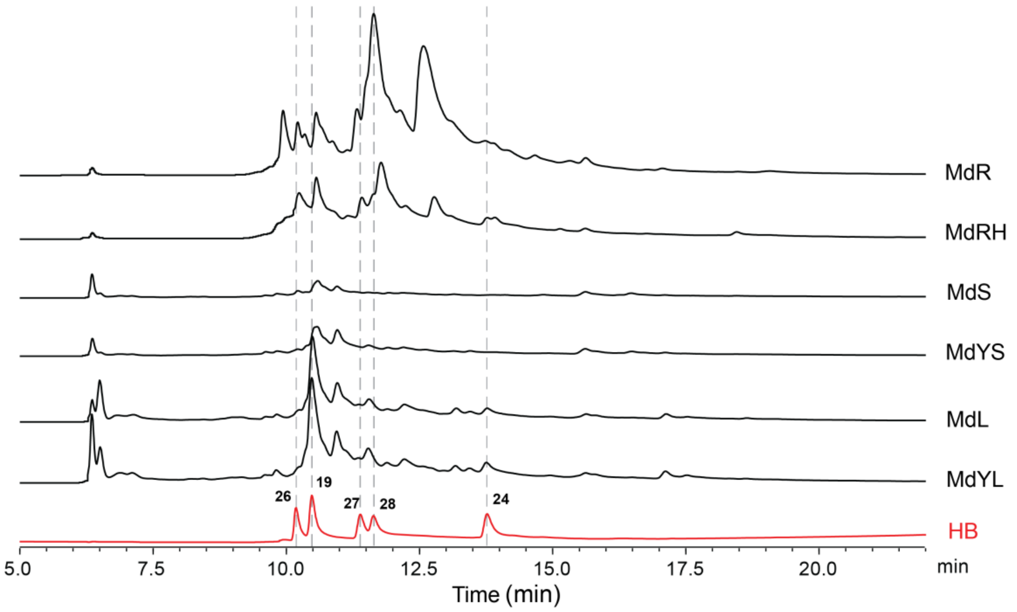

Fig. S26. HPLC analysis of total alkaloids extracted from various tissues of *M. dauricum*, including roots (MdR), root hairs (MdRH), lower stems (MdS), upper stems (MdYS), leaves (MdL), and young leaves (MdYL). HB standards signify a mixture of standards sinomenine (19), tetrandrine (24), guattegaumerine (26), daurisoline (27) and dauricine (28).

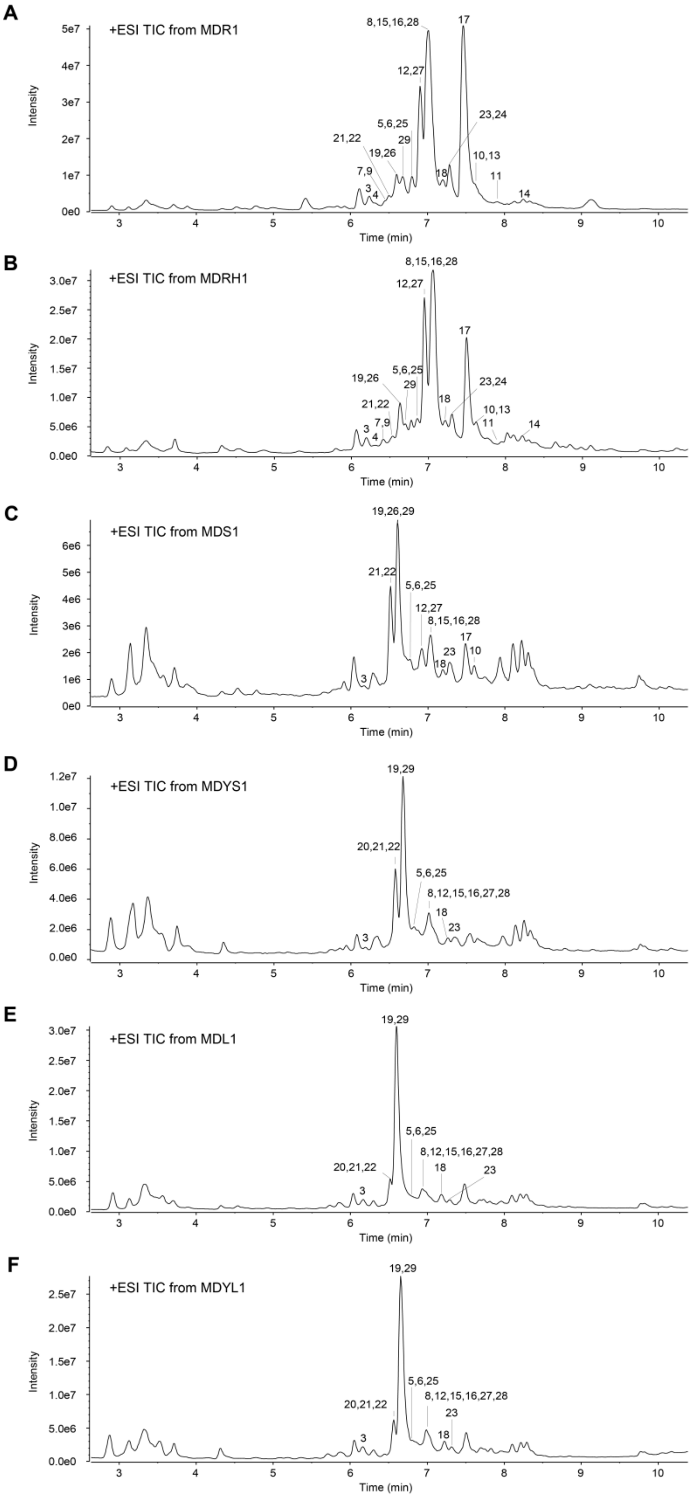

Fig. S27. LC-MS/MS total ion flow chart of total alkaloids extracted from different tissues of *M. dauricum*, including root (MdR1, A), root hair (MdRH1, B), stem (MdS1, C), young stem (MdYS1, D), leaf (MdL1, E) and young leaf (MdYL1, F). The chemical numbers represent the different BIA compounds, which is listed in Supplementary Table 8.

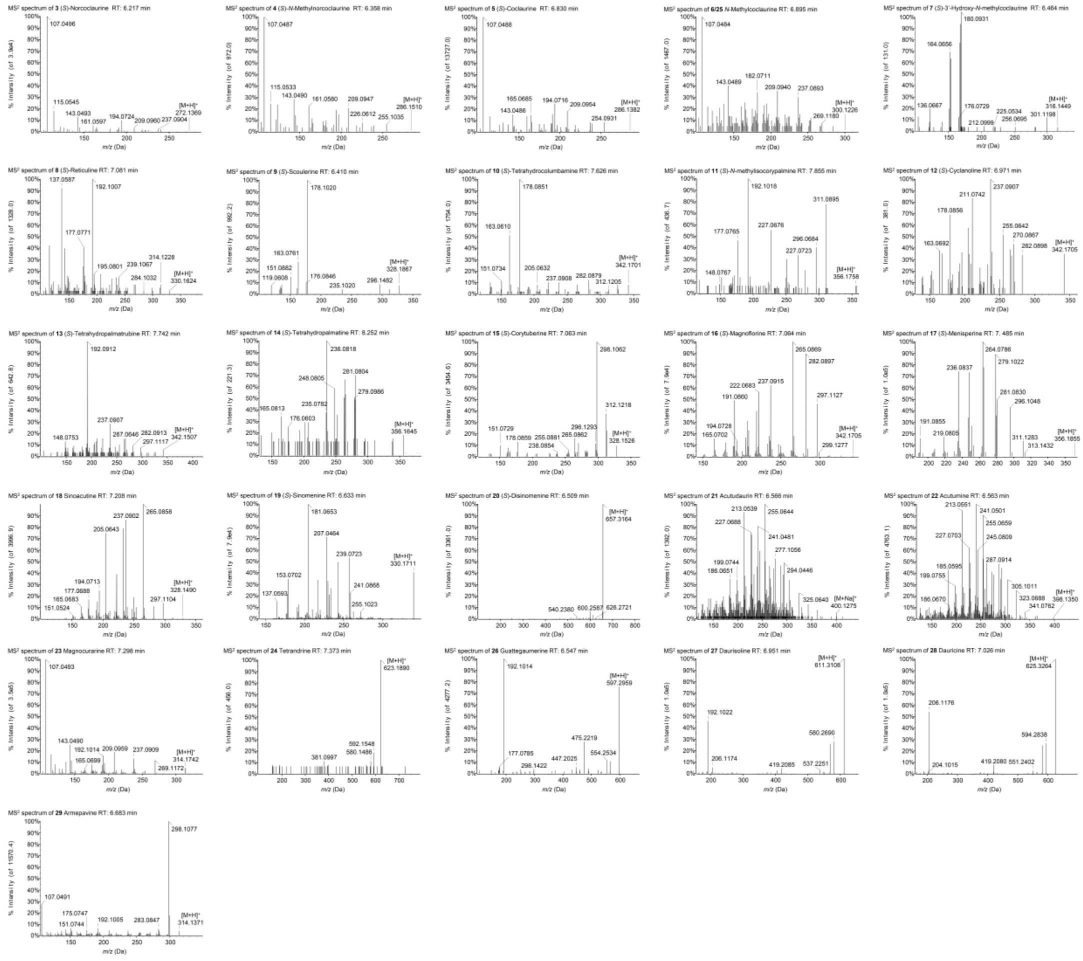

Fig. S28. MS^2 spectrum of BIAs identified from *M. dauricum*.

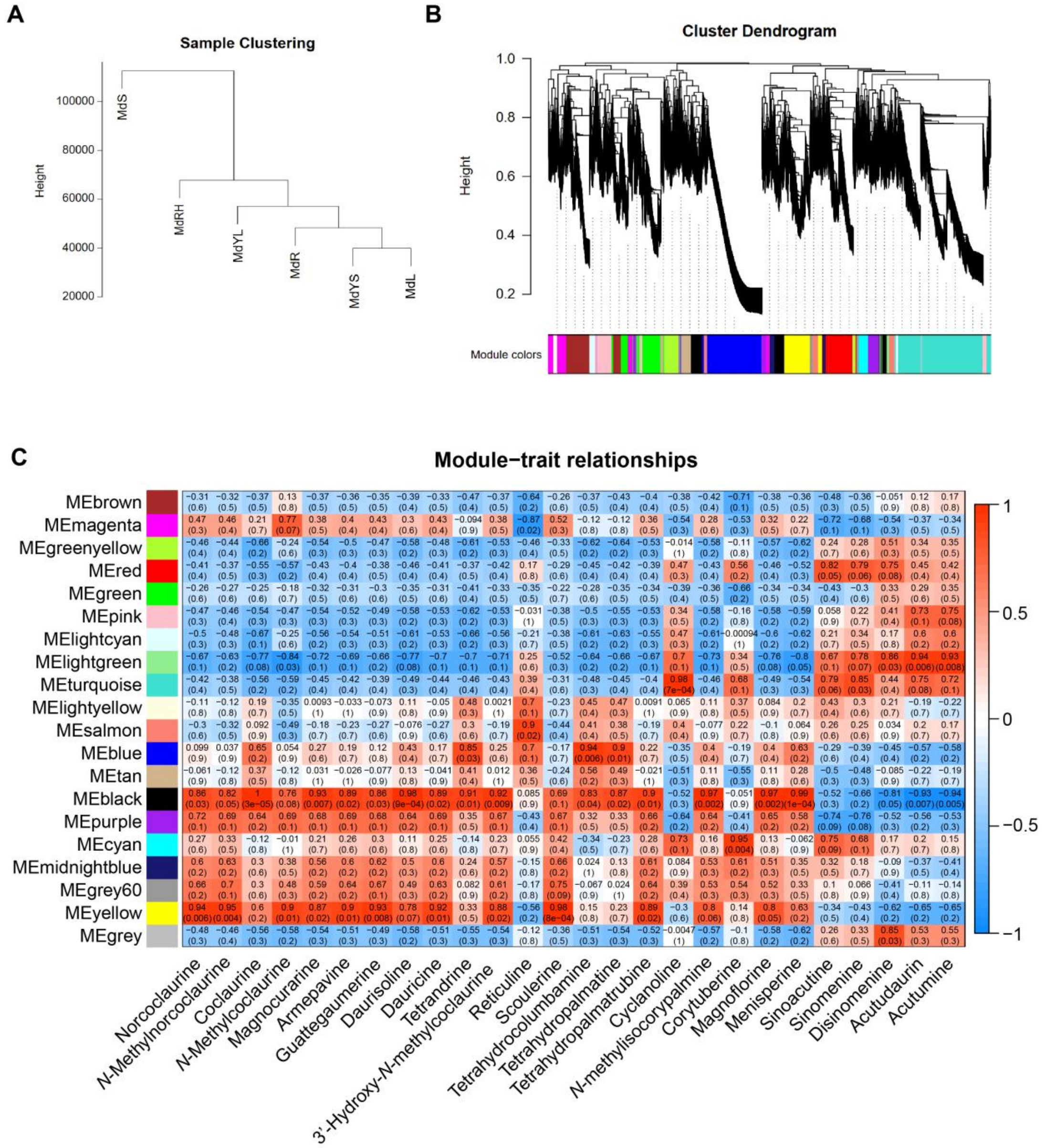

Fig. S29. Integrative analysis of metabolome and transcriptome revealed the correlation between BIAs and candidate genes in different tissues of *M. dauricum*. (**A**) Sample clustering for different tissues from WGCNA. (**B**) Clustering dendrogram of genes, with dissimilarity based on topological overlap, together with assigned merged module colors. The different colors under the dendrogram show co-expression modules identified using WGCNA. (**C**) Relating modules to the pathway-related genes identified in *M. dauricum*. Each cell contains the corresponding correlation and *p*-value. In the heatmap, blue color represents negative correlation, while red represents positive correlation.

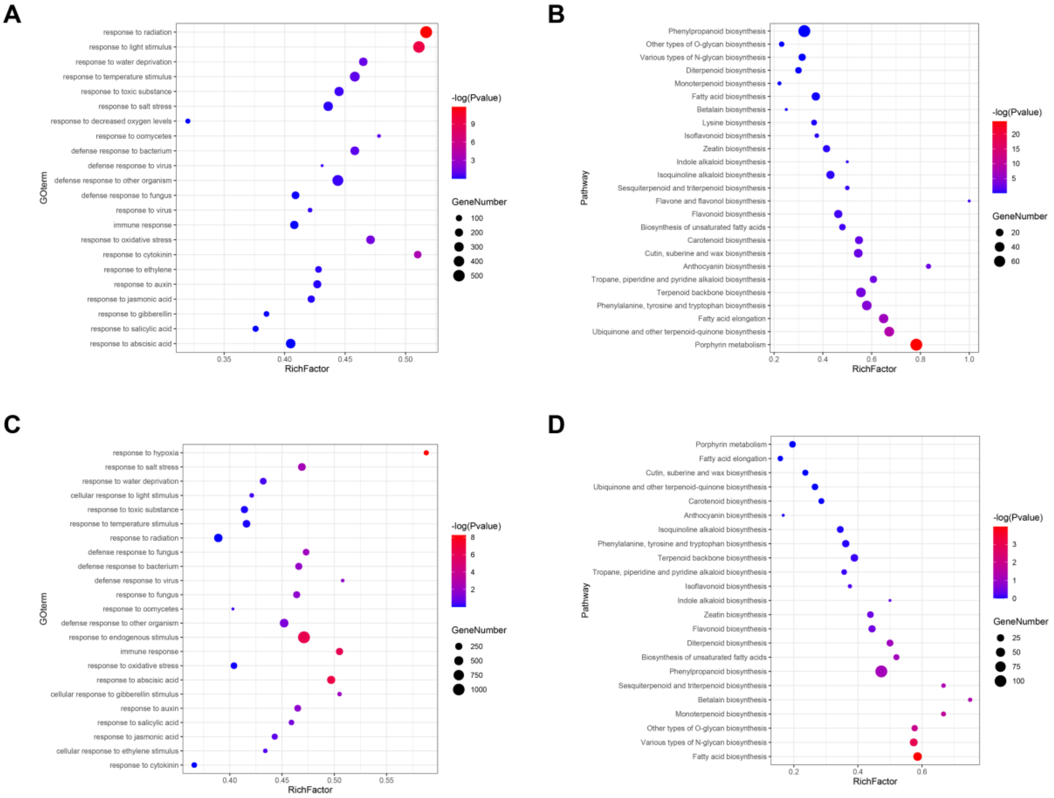

Fig. S30. Gene ontology (GO) and KEGG pathway enrichment analysis of different gene sets obtained by WGCNA. (**A-B**) GO and KEGG pathway enrichment analysis of gene that are positively associated with the biosynthesis of BIAs enriched in aerial tissues of *M. dauricum*. (**C-D**) GO and KEGG pathway enrichment analysis of gene obtained by WGCNA that are positively associated with the biosynthesis of BIAs enriched in roots of *M. dauricum*.

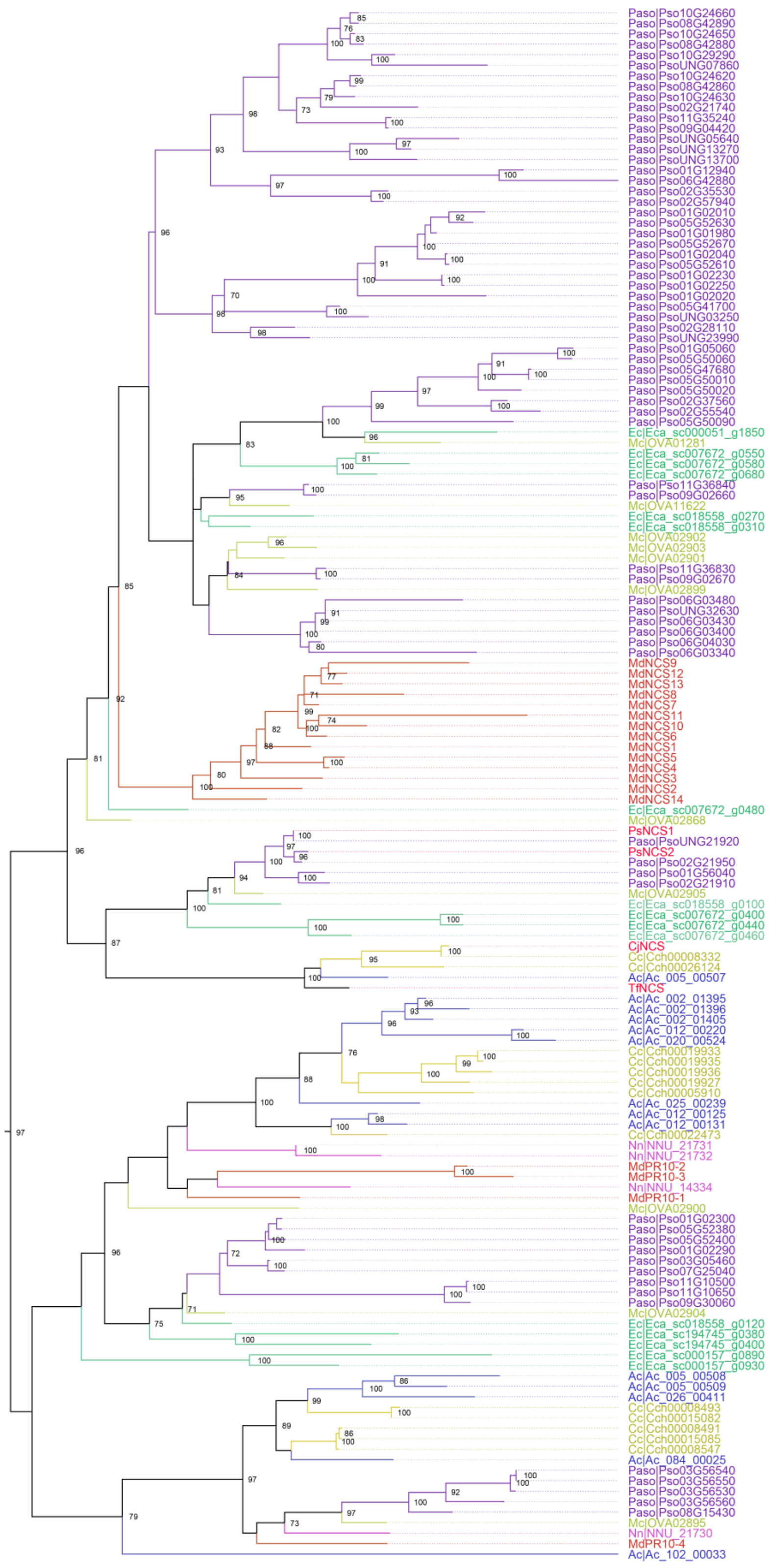

Fig. S31. The phylogenetic relationship of NCSs from *M. dauricum* and other tested species, including *A. coerulea* (Ac), *C. chinenesis* (Cc), *C. japonica* (Cj), *M. dauricum* (Md), *T. thalictroides* (Tt), *P. somniferum* (Ps/Paso), *M. cordata* (Mc), *E. californica* (Esca), *N.*

***nucifera* (Nenu).** The numbers on the branch represent nodes with a support value greater than 70%.

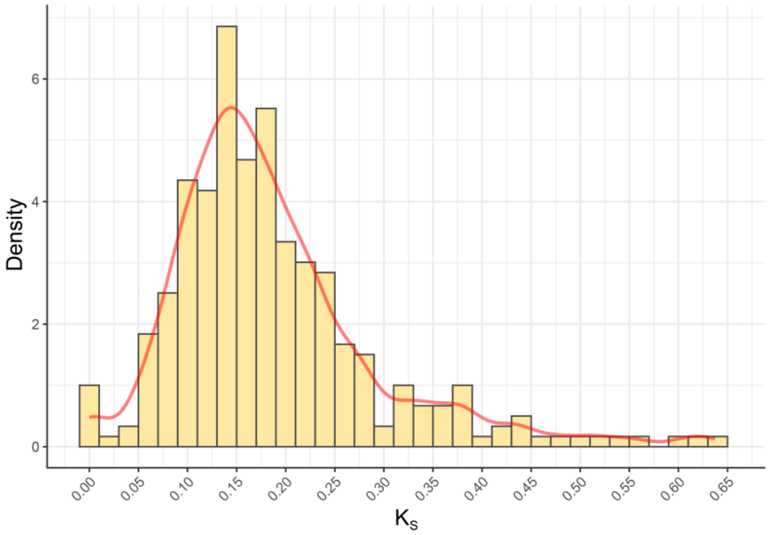

Fig. S32. K_S distribution plot of paralogs in synteny blocks of *MdNCSs*.

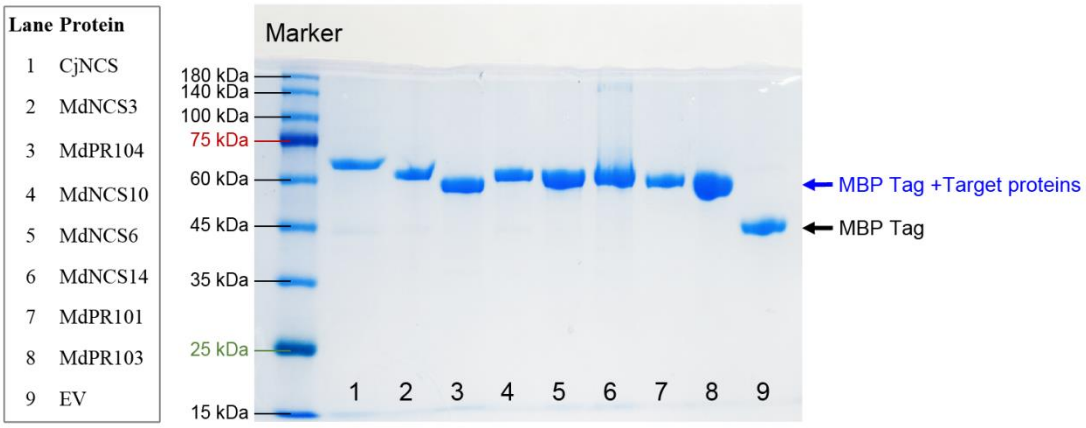

Fig. S33. SDS-PAGE analysis of heterologous expression of NCS proteins in *E. coli*. The purified proteins were loaded onto 5% stacking gel and 12% separating gel. A wide molecular weight standard protein (10-180 kDa) acts as marker.

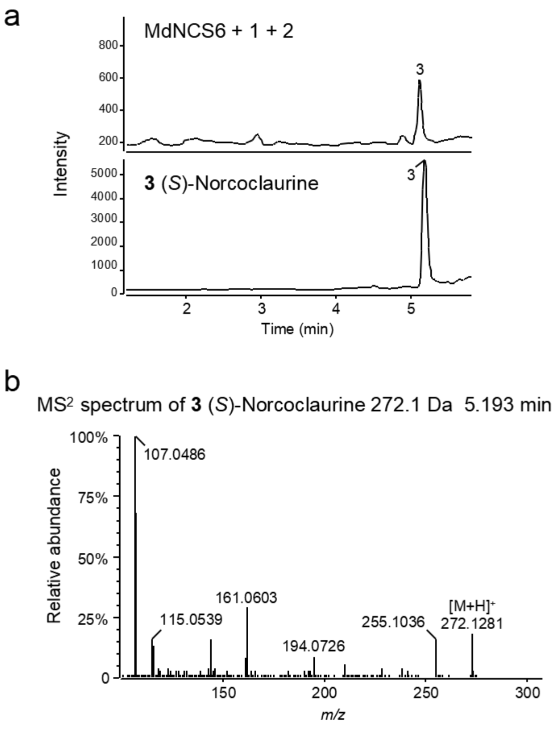

Fig. S34. LC-MS/MS analysis of *in vitro* catalytic assays of MdNCS6 using dopamine 1 and 4-HPAA 2 as substrate.

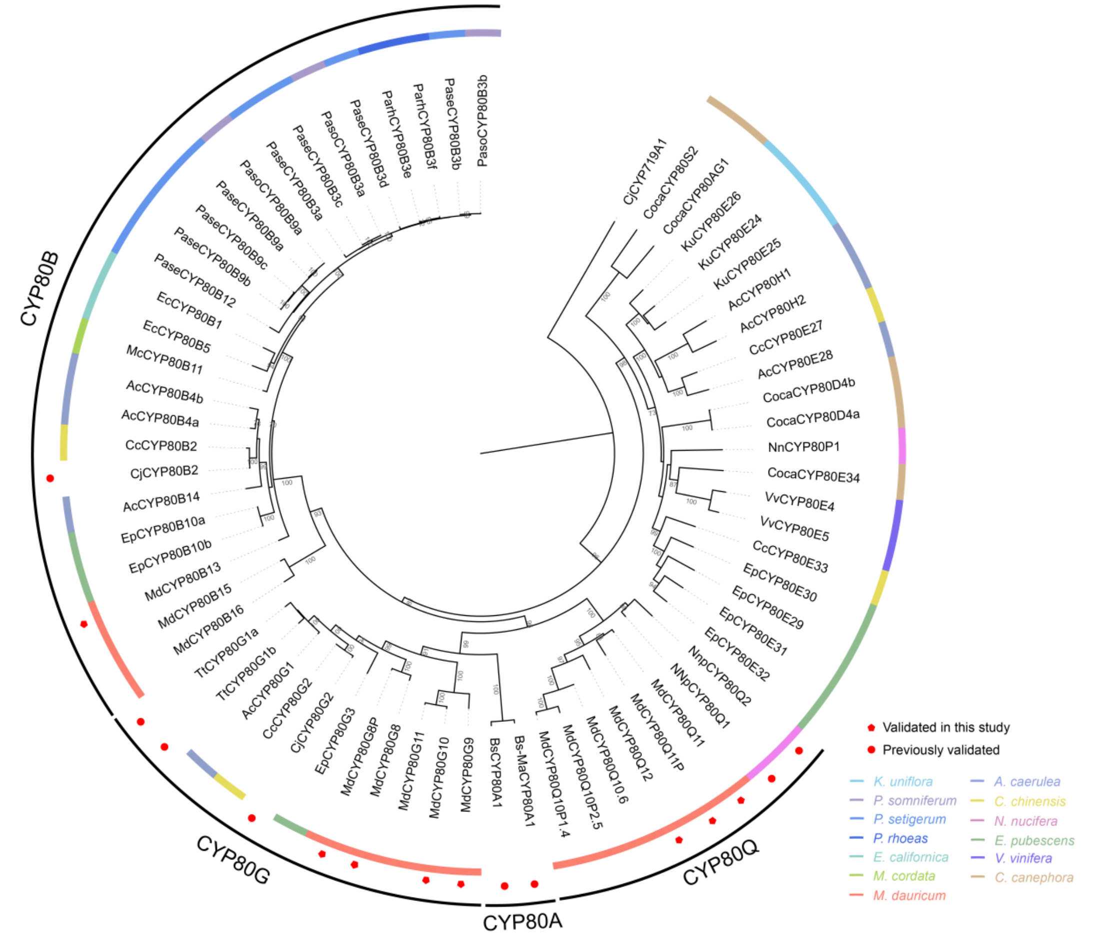

Fig. S35. The phylogenetic relationship of CYP80s from *M. dauricum* and other tested species, including *A. coerulea*, *C. chinenesis*, *C. japonica*, *M. dauricum*, *E. pubescens*, *T. thalictroides*, *K. uniflora*, *B. stolonifera* (Bs), *P. somniferum*, *P. setigerum*, *P. rhoeas*, *M. cordata*, *E. californica*, *N. nucifera*, *C. canephora*, *V. vinifera*. The numbers on the branch represent nodes with a support value greater than 70%.

Lane	Protein
1	MdCYP80Q12
2	MdCYP80B13
3	MdCYP80G8
4	MdCYP80G10
5	Negative Control (EV)

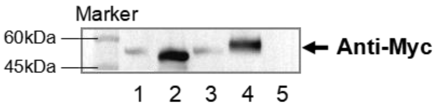

Fig. S36. The expression of CYP450 protein expression in yeast examined by Western blot analysis.

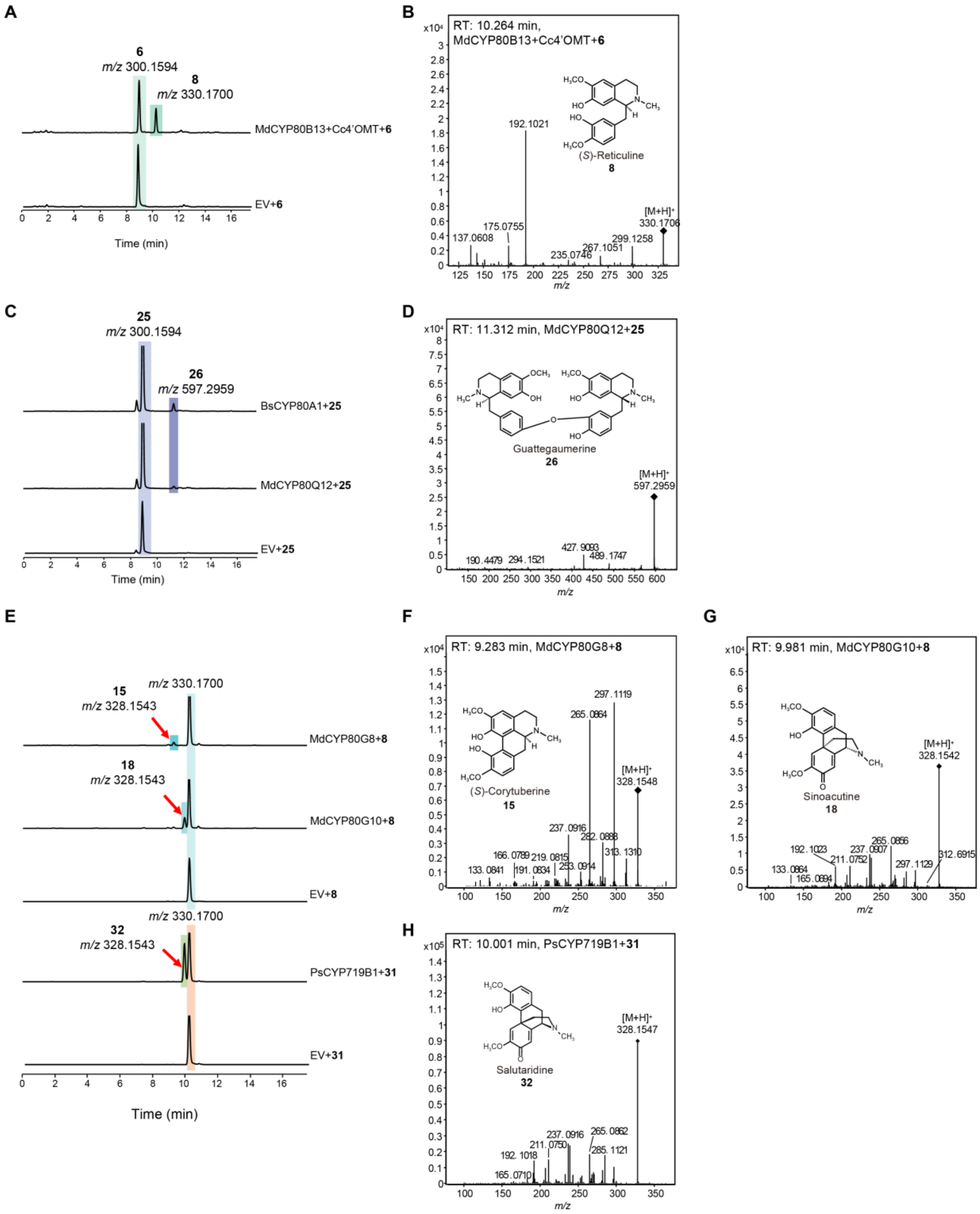

Fig. S37. Transient expression of candidate MdCYP80s and PsCYP719B1 in *Nicotiana benthamiana*. (A-B) LC-MS/MS analysis of MdCYP80B13 and Cc4'OMT co-injected into the leaves of *N. benthamiana* using (*S*)-*N*-methylcoclaurine **6** as the substrate. **(C-D)** LC-MS/MS analysis of BsCYP80A1 and MdCYP80Q12 injected into *N. benthamiana* using (*R*)-*N*-methylcoclaurine **25** as the substrate, respectively. **(E-H)** LC-MS/MS analysis of MdCYP80G8, MdCYP80G10 (using (*S*)-reticuline **8** as the substrate) and PsCYP719B1 (using (*R*)-reticuline **31** as the substrate) injected into *N. benthamiana*, respectively.

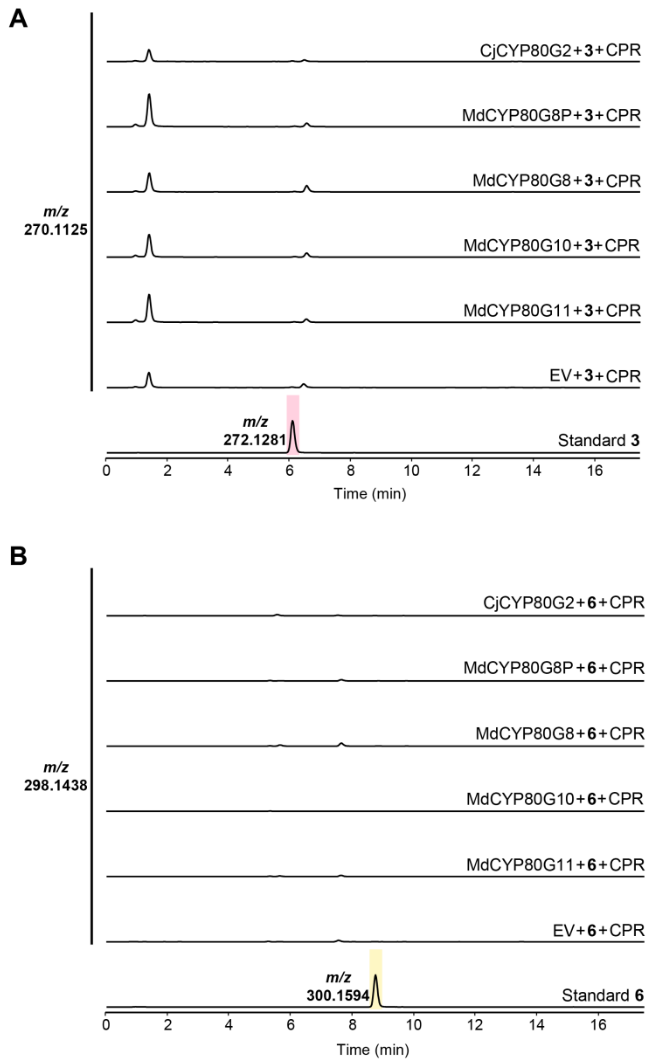

Fig. S38. LC-MS/MS analysis of *in vitro* catalytic assays of MdCYP80Gs using (*S*)-norcoclaurine 3 and (*S*)-*N*-methylcoclaurine 6 as substrate, respectively.

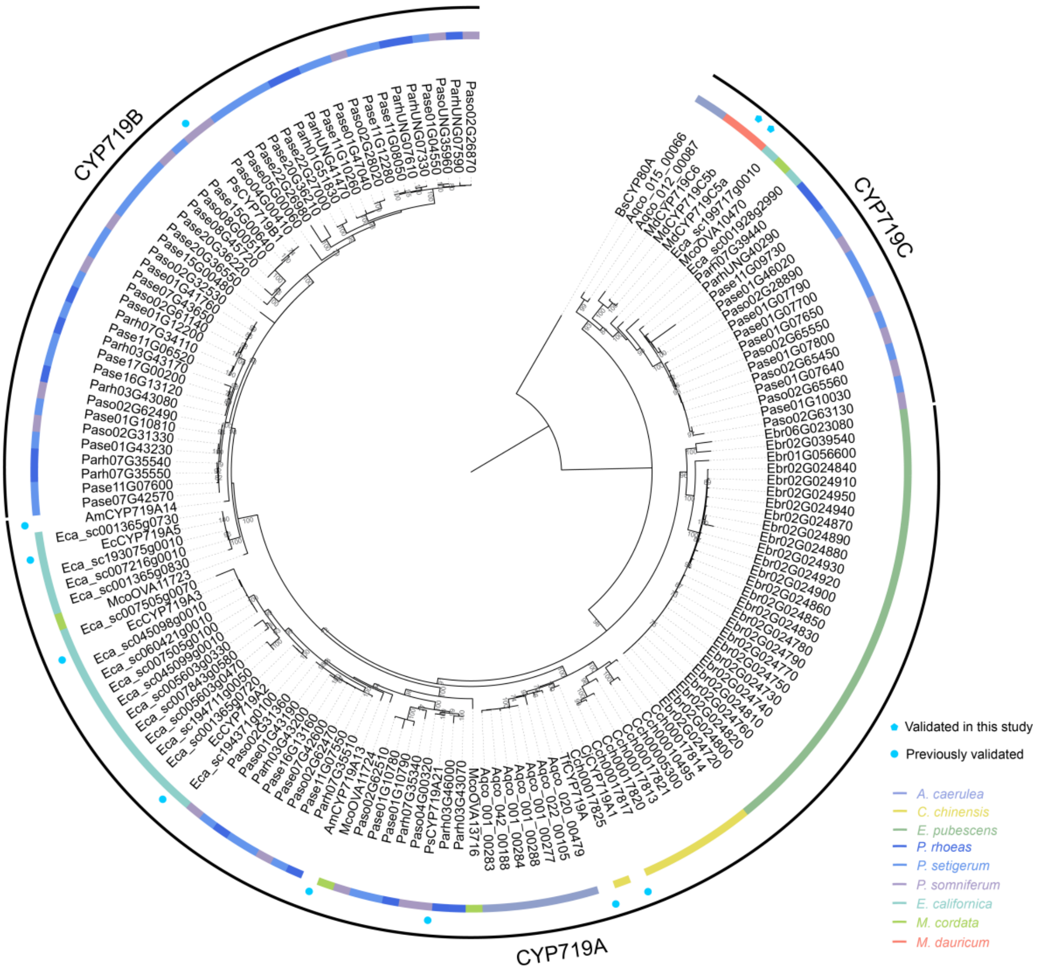

Fig. S39. The phylogenetic relationship of CYP719s from *M. dauricum* and other tested species, including *A. coerulea*, *C. chinenesis*, *C. japonica*, *M. dauricum*, *E. pubescens*, *T. thalictroides*, *K. uniflora*, *A. mexicana* (Am), *P. somniferum*, *P. setigerum*, *P. rhoeas*, *M. cordata*, *E. californica*, *N. nucifera*, *C. canephora*, *V. vinifera*. The numbers on the branch represent nodes with a support value greater than 70%.

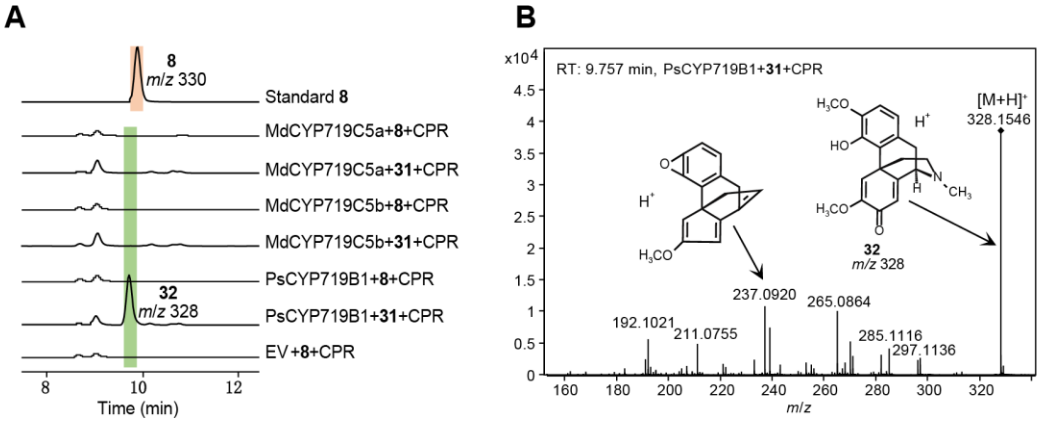

Fig. S40. Catalytic analysis of MdCYP719C5a, MdCYP719C5b, and PsCYP719B1 using (*S*)-reticuline 8 and (*R*)-reticuline 31 as substrate. (A) The LC-MS extracted ion chromatograms at m/z 330 and m/z 328 represent the (*S*)-reticuline **8** and the potential C-C linked products, respectively. **(B)** MS/MS fragmentation of salutaridine **32**, which is the catalytic product of PsCYP719B1 activity with the substrate (*R*)-reticuline **31**.

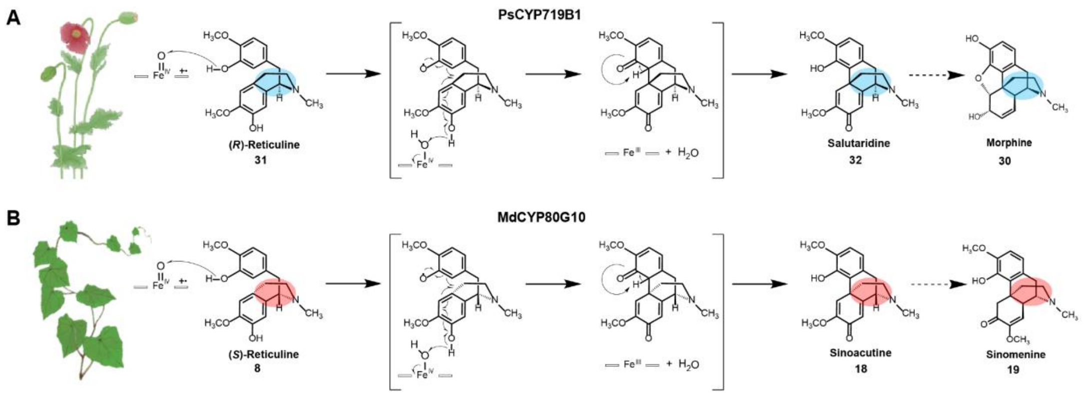

Fig. S41. Proposed catalytic mechanism of *Papaver*-specific PsCYP719B1 from (*R*)-reticuline 31 to salutaridine 32 (A) and *Menispermum*-specific MdCP80G10 from (*S*)-reticuline 8 to sinoacutine 18 (B).

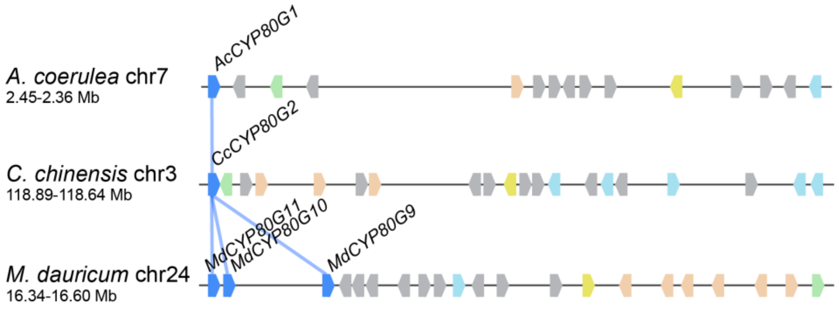

Fig. S42. Collinearity analysis of *CYP80Gs* in *M. dauricum*. The homologous genes are color-coded, with connected lines indicating collinearity between *CYP80s*.

Table S1. Statistics of the contig-level and chromosome-level *M. dauricum* genome assembly.

Item	The contig-level genome assembly	The contig-level genome assembly (purged)	The chromosome-level genome assembly
Total sequences	1156	253	29
Unmapped sequence	-	-	3
Total bases	887,047,541	675,676,968	675,699,368
Min sequence length (bp)	1,365	14,018	7,124,370
Max sequence length (bp)	14,476,436	14,476,436	39,468,064
Average sequence length (bp)	767,342.16	2,670,659.95	23,299,978.21
Median sequence length (bp)	53,804	1,595,894	24,547,578
N25 length (bp)	7,972,555	8,551,055	28,048,869
N50 length (bp)	4,850,569	5,934,026	25,849,843
N75 length (bp)	2,435,212	3,184,327	21,680,124
N90 length (bp)	737,176	1,779,198	18,586,060
N95 length (bp)	218,865	1,181,754	17,622,569
As	32.46%	32.44%	32.43%
Ts	32.47%	32.42%	32.43%
Gs	17.54%	17.56%	17.57%
Cs	17.56%	17.58%	17.56%
(A + T)s	64.93%	64.86%	64.86%
(G + C)s	35.07%	35.14%	35.13%

Table S2. Statistics of chromosome-scale pseudomolecules and unmapped scaffolds.

ID	Length	N_counts	LowCase_counts	GC_counts
chr1	39468064	800	0	13808366
chr2	29994229	600	0	10604597
chr3	32338906	1800	0	11419479
chr4	26453263	600	0	9297928
chr5	28629350	1223	0	10156548
chr6	21121502	1623	0	7399534
chr7	28666703	1300	0	10139223
chr8	28048869	700	0	9798644
chr9	27554617	823	0	9710426
chr10	26733387	500	0	9392152
chr11	26589019	1000	0	9343713
chr12	25890630	800	0	9045810
chr13	25849843	800	0	9014380
chr14	25660236	900	0	8985712
chr15	24547578	500	0	8627534
chr16	24647288	1446	0	8706145
chr17	24023304	1023	0	8500467
chr18	23427390	700	0	8204850
chr19	22283725	700	0	7784772
chr20	20795511	300	0	7269411
chr21	20718214	823	0	7235845
chr22	18888880	600	0	6617640
chr23	18586060	1023	0	6506865
chr24	21680124	500	0	7680905
chr25	17622569	800	0	6217543
chr26	17811658	700	0	6297767
utg1	12920384	0	0	4497199
utg2	7623695	0	0	2670883
utg3	7124370	0	0	2468373

Table S3. Assessing the completeness of genome assembly and annotation results for *M. dauricum* genome with Benchmarking Universal Single-Copy Orthologs (BUSCO) analysis.

BUSCO notation	Genome assembly		Annotation results	
	Number	Percent	Number	Percent
Complete BUSCOs (C)	1547	95.8%	1497	92.80%
Complete and single-copy BUSCOs (S)	1038	64.3%	868	53.80%
Complete and duplicated BUSCOs (D)	509	31.5%	629	39.00%
Fragmented BUSCOs (F)	15	0.9%	26	1.60%
Missing BUSCOs (M)	52	3.3%	91	5.60%
Total BUSCO groups searched	1614	100%	1614	100%

Table S4. Statistics of repetitive element content in the *M. dauricum* genome.

Item	Subfamily	Number	Length(bp)	Coverage
SINEs	/	0	0	0.00%
LINEs	/	88,956	49,992,153	7.40%
LTR elements	/	336,888	228,369,084	33.80%
	Copia	173,064	162,915,184	24.11%
	Gypsy	33,885	23,929,661	3.54%
DNA transposons	/	25,314	24,981,252	3.70%
Unclassified	/	387,609	119,059,359	17.62%
Total interspersed repeats	/	/	422,401,848	62.51%
Simple sequence repeats	/	126,050	7,572,227	1.12%
Low complexity		20,520	998,756	0.15%

Table S5. Plant genomes for phylogenetic and comparative genomics analyses.

Plant taxon	Reference
Vitis vinifera	http://www.genoscope.cns.fr/externe/GenomeBrowser/Vitis/
Coffea canephora	https://coffee-genome-hub.southgreen.fr/node/1/2
Nelumbo nucifera	http://nelumbo.biocloud.net/nelumbo/home
Aquilegia coerulea	https://www.ncbi.nlm.nih.gov/datasets/genome/GCA_002738505.1/
Coptis chinensis	https://www.ncbi.nlm.nih.gov/datasets/genome/GCA_015680905.1/
Epimedium pubescens	https://download.cncb.ac.cn/gwh/Plants/Epimedium_pubescens_IMPLAD_EP_1.0_GWHBECS00000000/
Menispermum dauricum	The present study
Kingdonia uniflora	https://www.ncbi.nlm.nih.gov/datasets/genome/GCA_014058105.1/
Akebia trifoliata	https://download.cncb.ac.cn/gwh/Plants/Akebia_trifoliata_Shusen1_GWHBISH00000000/
Eschscholzia californica	https://eschscholzia.kazusa.or.jp/cgi-bin/list.cgi
Papaver rhoeas	https://github.com/xjtu-omics/Papaver-Genomics
Macleaya cordata	https://www.ncbi.nlm.nih.gov/datasets/genome/GCA_002174775.1/
Amborella trichopoda	https://phytozome-next.jgi.doe.gov/info/Atrichopoda_v1_0

Table S6. Assessing the assembly continunity for *M. dauricum* and published genomes of Ranunculales using LTR assembly index (LAI). Intact LTR-RT and Total LTR-RT respectively represent the proportion of intact LTR-RT and total LTR-RT in genomes.

Species	Genome size	Intact LTR-RT	Total LTR-RT	raw_LAI	LAI
P. rhoeas	2,542,266,437	0.1575	0.6973	22.59	23.4
P. somniferum	2,712,531,559	0.0396	0.6454	6.14	5.08
P. setigerum	4,590,117,772	0.0221	0.6474	3.41	4.38
E. californica	489,064,912	0.0195	0.2936	6.65	8.7
M. cordata	377,833,576	0.0123	0.2782	4.42	3.74
A. coerulea	306,516,179	0.0587	0.3071	19.1	14.38
C. chinensis	935,659,790	0.0754	0.5503	13.71	14.77
M. dauricum	675,699,368	0.0519	0.52	9.97	11.49
K. uniflora	1,004,558,319	0.018	0.5726	3.14	8.77

Table S7. Chromosomal fusions compared to AEK among *M. dauricum, A. coerulea, C. chinensis, P. rhoeas* and *V. vinifera*.

Species	chr1	chr2	chr3	chr4	chr5	chr6	chr7
M. dauricum	2	0	1	2	0	0	1
A. coerulea	1	3	5	6	6	2	6
C. chinensis	5	3	7	5	12	14	17
P. rhoeas	31	6	37	25	25	22	18
V. vinifera	1	0	3	0	4	4	4

Table S8. Information of BIAs in *M. dauricum* by LC-MS/MS.

Index	Mass (Da)	Molecular Weight (Da)	Formula	Ionization model	Retention Time (min)	Compounds	Class I	Class 2	CAS
3*	272.034	271.31	$C_{16}H_{17}NO_3$	$[M+H]^+$	6.2 min	Norcoclaurine	Alkaloids	Benzylisoquinolines	22672-77-1
4	286.0517	285.34	$C_{17}H_{19}NO_3$	$[M+H]^+$	6.3 min	*N*-Methylnorcoclaurine	Alkaloids	Benzylisoquinolines	\
5*	286.0519	285.34	$C_{17}H_{19}NO_3$	$[M+H]^+$	6.8 min	Coclaurine	Alkaloids	Benzylisoquinolines	486-39-5
6*+ 25*	300.0700	299.36	$C_{18}H_{21}NO_3$	$[M+H]^+$	6.9 min	(*S*)-*N*-Methylcoclaurine & (*R*)-*N*-Methylcoclaurine	Alkaloids	Benzylisoquinolines	3423-07-2 5096-70-8
7*	316.0763	315.36	$C_{18}H_{21}NO_4$	$[M+H]^+$	6.5 min	3'-Hydroxy-*N*-methylcoclaurine	Alkaloids	Benzylisoquinolines	1936-17-0
8*	330.0894	329.39	$C_{19}H_{23}NO_4$	$[M+H]^+$	7.1 min	Reticuline	Alkaloids	Benzylisoquinolines	485-19-8
9*	328.0734	327.37	$C_{19}H_{21}NO_4$	$[M+H]^+$	6.4 min	Scoulerine	Alkaloids	Protoberberines	6451-73-6
10*	342.0898	341.4	$C_{20}H_{23}NO_4$	$[M+H]^+$	7.6 min	Tetrahydrocolumbamine	Alkaloids	Protoberberines	483-34-1
11	356.0711	356.4	$C_{21}H_{26}NO_4^+$	$[M]^+$	7.8 min	*N*-methylisocorypalmine	Alkaloids	Protoberberines	\
12	342.093	342.4	$C_{20}H_{23}NO_4^+$	$[M]^+$	7.0 min	Cyclanoline	Alkaloids	Protoberberines	18556-27-9
13	342.0917	341.4	$C_{20}H_{23}NO_4$	$[M+H]^+$	7.7 min	Tetrahydropalmatrubine	Alkaloids	Protoberberines	18090-68-1
14*	356.0682	355.43	$C_{21}H_{25}NO_4$	$[M+H]^+$	8.2 min	Tetrahydropalmatine	Alkaloids	Protoberberines	483-14-7
15	328.1079	327.37	$C_{19}H_{21}NO_4$	$[M+H]^+$	7.1 min	Corytuberine	Alkaloids	Protoberberines	517-56-6
16*	342.0918	342.41	$C_{20}H_{24}NO_4^+$	$[M]^+$	7.1 min	Magnoflorine	Alkaloids	Aporphines	2141-09-5
17	356.1111	356.43	$C_{21}H_{26}NO_4^+$	$[M]^+$	7.5 min	Menisperine	Alkaloids	Aporphines	25342-82-9
18*	328.0916	327.37	$C_{19}H_{21}NO_4$	$[M+H]^+$	7.2 min	Sinoacutine	Alkaloids	Sinomenines	4090-18-0
19*	330.1096	329.39	$C_{19}H_{23}NO_4$	$[M+H]^+$	6.6 min	Sinomenine	Alkaloids	Sinomenines	115-53-7
20	657.3164	656.76	$C_{38}H_{44}N_2O_8$	$[M+H]^+$	6.5 min	Disinomenine	Alkaloids	Sinomenines	596-58-7
21	400.0898	377.4	$C_{20}H_{27}NO_6$	$[M+Na]^+$	6.5 min	Acutudaurin	Alkaloids	Acutumines	\

Index	Mass (Da)	Molecular Weight (Da)	Formula	Ionization model	Retention Time (min)	Compounds	Class I	Class 2	CAS
22	398.0916	397.85	$C_{19}H_{24}NO_6Cl$	$[M+H]^+$	6.5 min	Acutumine	Alkaloids	Acutumines	17088-50-5
23*	314.0894	314.4	$C_{19}H_{24}NO_3^+$	$[M]^+$	7.3 min	Magnocurarine	Alkaloids	Benzylisoquinolines	6801-40-7
24*	623.3273	622.75	$C_{38}H_{42}N_2O_6$	$[M+H]^+$	7.4 min	Tetrandrine	Alkaloids	Bisbenzylisoquinolines	518-34-3
26*	597.3036	596.71	$C_{36}H_{40}N_2O_6$	$[M+H]^+$	6.5 min	Guattegaumerine(Dauriciline)	Alkaloids	Bisbenzylisoquinolines	21446-35-5
27*	611.3225	610.74	$C_{37}H_{42}N_2O_6$	$[M+H]^+$	6.9 min	Daurisoline	Alkaloids	Bisbenzylisoquinolines	70553-76-3
28*	625.3445	624.76	$C_{38}H_{44}N_2O_6$	$[M+H]^+$	7.1 min	Dauricine	Alkaloids	Bisbenzylisoquinolines	524-17-4
29*	314.0858	313.39	$C_{19}H_{23}NO_3$	$[M+H]^+$	6.7 min	Armepavine	Alkaloids	Benzylisoquinolines	5884-67-3

*Compounds confirmed to be in conformity with the standards.

Table S9. Statistics table of NCS gene number annotated from *M. dauricum*, *P. somniferum*, *E. californica*, *M. cordata*, *A. caerulea*, *C. chinensis*, and *N. nucifera*.

Species	PR10 gene number	NCS gene number
M. dauricum	18	14
P. somniferum	68	54
E. californica	16	11
M. cordata	11	8
A. coerulea	28	1
C. chinensis	13	2
N. nucifera	4	0
V. vinifera	1	0
C. canephora	4	0
A. trichopoda	2	0

Table S10. Gene seqeunces of CYP80 family members that underwent a large expansion in *M. dauricum*.

Gene ID	Sequence
MdCYP80B13	ATGGAGATAGTCTCTACTGTAGCTATTGGTTTCATCTTCTTCAT CATCTTCGTCTTCTTCTTCAGCAACCAAAGATCAACAAAGGGT CTCCCTCCAGGTCCTAAACCATGGCCAATTGTGGGCAATCTCC TCCAACTAGGAGACCAACCTCATGCTCAATTCGCCCAACTCTC CCAAACATATGGCCCTCTCTTCTCCCTCAAACTTGGGTCTCAA ACAGTAGTTGTGGCCTCCTCACCATCTGCAGCCACTCAAGTCC TCAAGACCCACGATCGTGTGCTCTCCGGTCGCTACGTCTTCCA GAGCTTTCGCATCGACAGGCATGTCAACAACTCCATTGTCTGG TCTGAGTGCAATGACAATTGGAAGCTGTTGAGAAAAGTTTGTA GGACTGAAGTGTTCTCACCAAAGATGATAGAGTCCCAAGCTC ACTTAAGAGAGGCTAAGGCAATTGAGATGGTGGAGTTTTTGA AAGGGAGAGAAGGGCAGGTTCTGAAGATTGCTGAAGTGGTGT TTGGAACACTTGTGAACATCTTTGGCAACTTGATATTCTCTCA AGATGTTTTTGACTTGGCTGATCCTACTAGTGGGAGTGCTGAG ATGAAGGAGCATATATGGAGGATGTTGGAGCTGGGGAACTCT ACTAATCCTGCTGATTACTTCCCAATTATGGGCAAGCTTGATT TGTTCGGACAGCGGAGAGCCGTCGCCGAGTGTCTTCAACAGA TTTACGATGTTTGGGGGGCTATGTTGAAGGAGCGGCGCGCTAC GAAAGGAACTGAGACTAATAACGACTTCGTTAATGTGTTGCTC GAAGCCGGTCTCGACGATCAAAGGATCAATTCCTTGCTCATGG AATTATTTGCAGCAGGAACAGAGACAAGTGCATCGACAATCG AGTGGGCGATAGCGGAGCTAACGAAAAACCCACAAATAATGG CCAAAATACACAGTGAATTGGAGAGTGTAGTAGGCAAAGAGA GGGTGAAAGAGTCACACATTCCTCACCTCCCTTACCTCCAAGC CTTTGTAAAGGAGACACTAAGACTACACCCAGCCACACCTAT GCTTCTTCCTCGCCGAGCTCTTGAAACATGTAATGTGATGGGC TACACCATCCCGAAAGACTGCCAAATAATGGTGAACGCCTGG GCGATTGCGCGAGACCCAAAAGTGTGGAAGGATCCACTGAAG TTCTCACCAGAGAGGTTCT, respectivelyTGAATTCAAGCTTGGATTACAAAGGCAATGACTTTG AGTTCATACCATTTGGTGGAGGAAGGAGGATTTGCCCTGGCCT TCCATTAGCTACTCAGTTCATAAGCCTCATTGTGGGGACTCTT GTGCAGAATATGGACTGGAGTTTGCCTAATGGAATGGATCCTA GTGAGTTGGGGATGGAGGAGAAGTTTGGGCTGACACTGCAGA AGGAGCCACCTCTGCTTATAGTGCCTAAATCAAGAGATTTTTT GAATGAAACAAGAGGCTAA
MdCYP80B15	ATGATAGAAACGCTACTGCTCTTGTTCCTAACTCTCCTTCTTAG CCTAGTCTTTTTCTTGAAGAACTCATCATCCAAGAACCTCCCA CCAGGCCCACCTCCCTTCCCCATCATAGGCAACCTTCACCAAC TAGGCTCCAAACCTCACTCCACTCTTGCTCAATTGGCTCAAAC

Gene ID	Sequence
	CTATGGCCCTCTCTTCTCTCTAAGGTTTGGCTTCCAATTGGTAG<br>TCGTGGCTTCGTCCTCGGCGGCCGCATCGGAGGTCCTCAAGGC<br>CCATGACAACGTCCTCTCTGGCCGGCACATAACGTATAGCGCC<br>CGCTTGAAGAATTATGTCGAGCACTCGATGGTCTGGGCTCCGG<br>AGTGCACCGAGGCCTGGAAGAATCTCAGGCGAATTTGCCGGC<br>TCGAGCTGGTTTCGCCGAGAGCGATGGAGGCTCGCGCCGGTG<br>TGAGGGAGGTTAAGGTGGAGGAGATGGTGGGGTTTTTGAGAG<br>GGAAGGAAGGGGAGGTGGTGAAGGTGAGTGAAGTGGTGTTTG<br>GGACTATTTTTAATGCGCTTGGTGGCTTGATATTCTCTAAGGA<br>TGTGTTTGACATGAGAGGGGAAGGTGGTGGGGTTGTTAGGGA<br>CTTGAAGGTTCATTTGTGTAAGATGATGGAGTTGGGGAGTGCA<br>ATTAGTTTGGCAGATTGTTTTCCTAAACTTGGGTGGATGACTG<br>GGGTGAGAAGGGCTTCTGAAGAGTGTAGGAGGGATGTGTATG<br>GGAGTTGGGAGGAGATAATAAGGCAGAGAAGAGAAAGTAGT<br>ACTACTGGTGCAGGTCTTGGTGATGGATGTGATGATTATGATG<br>ATGATAGTAAGAAAGATTTTCTTAGTTCTTTGTTCAAAGCTGG<br>ATTCAGCAATGATCAGATCAATGCCTTGCTTCTGGACATTTTTT<br>GTGCTGCTTCGGATACAACTACTTCAACAATTGAGTGGGCTAT<br>GGCTGAAATAATGAAGAACCCACATGTCCTACCCAAGGTTCG<br>ATCTGAACTCCAATTAATATTGGAGACATCAAACAAGAATAT<br>AGCTGAGGCTGACCTCTCTCATCTCCCATATCTCCAAGCAACT<br>GTGAAGGAGACACTGAGACTGCACCCTCCAACACCTCTTCTGC<br>TGCCACGACGTGCATTACAGACATGTAAGGTCATGAACTACA<br>CTGTTCCAAAAGACTCCCAGATATTAGTGAATGCATGGGCTAT<br>TGGGAGAGAGACAAATGTGTGGGAAAATCCACTCAAATTCTG<br>GCCTGAAAGGTTTTTGGAGGATTATAAGTTGGGAAATAGTAGT<br>TTTGATTTTGTGCCATTTGGTGGTGGAAGGAGAATCTGTCCTG<br>GATTGCCCTTGGCTGCTCATCTTGTTCCTCTGATTTTAGGTTCT<br>TTGGTTCACAACTTTGATTGGTCCCTCCCAAGCGGCATGAACC<br>TTGATGACTTGACCACAGAAGAAAAATTTGGGTTGACCCTCCA<br>AAAGGACCCTCCTCTCCTCCTTGTTCCCAAGGCCAGACACTGA
MdCYP80B16	ATGCTAACAGTAGGATTGTTCTCAACTCTTCTTCTTCTCCTCCT<br>CTTTCTCTTCTTCTTCAGAGACTCATCATCCAAGAACCTCCCAC<br>CAGGTCCACCTCCCTTCCCCATCATAGGCAACCTTCACCAATT<br>AGGCTCCAAACCCCACTCCACTCTTGCACAATTGGCTCAAACC<br>TATGGTCCTCTCTTGTCTCTAAGGTTTGGCTCCCAATTGGTAGT<br>TATCGCGTCGTCCCCGGCAGCGGCATCGGAGGTCCTCAAGGTC<br>CACGACAACGTCCTCTCGGGCCGGCACATAATATACAACGCC<br>CGCTCTAAGAATTATGTCGAGCACTCGATGGTTTGGGCTCCAG<br>TTTGCAACGAGGCGTGGAAGAATCTTCGGCGGATCTGCCGGCT<br>CGAACTGTTTTCACCGAAGGCGATGGAGGCTCGCGCGGGTGT<br>GAGGGAGCTCAAGGTGGAGGAGATGGTGGGCGTTCTAAGAGG<br>GAAGGTAGGGGAGGTGGTGAAGGTGAGTGAGTTGGTGTTTGG

Gene ID	Sequence
	GACCATTTTCAATGTGCTTGGGGGCTTGATATTCTCTAAGGAT GTGTTTGACATGAGAAGGGAAGATGGTGTGGTTGTTGGGGAC TTGAAGGGTCATCTGTGTAAGATGTTGGAGTTGGGGAGTGCA GTTAGTTTGGCAGATTGTTTTCCTAAACTAGGGTGGATGACTG GGGGGAGAAAGGTATCTGAAGAGTGTAGGAGAAATGTGTTTG GGAGTTGGGAGGAGATAATAAGGGAGAGAAGAGAATTAAGT TCTAATTATGGTGTAGGACTTGGTGATGGATGTGATGATGATG ATGATGATGATGATGGTGCCAAGAAAGATTTTCTCAGTACTTT GATCAAAGCAGGATTCAGCAATGATCAGATCAATACCTTGCTT CTGGACATTTTTGGTGCTGCTGCAGATACAACTACTTCAACAA TCGAGTGGGCTATGGTGGAAATAATGAAGAACCCGCATGTAC TACACAAGCTTCAATCTGAACTACAATTAATATTAATGGAGAC ATCATCAAAGAAGAACAATATTATAACTGAGGCCGACCTCTC GCATCTCCCATATCTTCAAGCAACTGTGAAGGAGACACTGAG ACTGCACCCTCCAACACCTCTTCTGCTGCCTCGACGTGCATTA CAGACATGTAAGGTCATGAACTACACTGTCCCAAAAAACTCC CAAATATTAGTGAATGCATGGGCTATTGGGAGAGACACAAAT GTGTGGGAAAATGCACTCAAATTCTGGCCTGAAAGGTTTTTGG AGGACTATAAGTTGGGAAATAATAGTTTTAATTTTGTGCCATT TGGTGGTGGAAGGAGAATTTGTCCTGGGATGCCCTTAGCTGCT GAACTTACTCCCCTGATTTTGGGTTCTCTGTTTCATAACTTTGA TTGGTCTCTCCCAATGGGCATGACTCCTTATGACTTGACCACA GAAGAAAAATTTGGGTTGACTCTCCAAAAGGACCCTCCTCTCC TCCTTGTTCCTAAGGAGAGGCACTGA
MdCYP80Q10P1.4	ATGGCTCTACTAGCTCTGTTCCTTTTCTTTGCTCTTCCCATTCTC TTGCTTTACTTCCTCAAACCCTCTTCTCCCAAAAACCTTCCTCC AGGCCCATTTTCATGGCCTTTGCTTGGAACCTTGGGGCTGAAA TCAAACGAAAAACAAGCCCACGTCGTGCTAACCAATTTGGCT CAAACCTATGGCCCGCTCATGCTGCTAAAGCTCGGAGTTAAGC CTGTGATAGTGGCATCAACCCACGTTGCAGCCATGGAGATCCT CAAGACACAAGACCACGTTCTGTCCGGTCGTTGCCCCCCGCAT AGCGTGCAAGTCGAGGACTACATCGAGGCCTCAGTAGTTTGG GCGGACCGTAATGAACACTGGAAGATGGTGAGGAAGATATGC AGGACGGAACTGTTCTCTACTAAGATGTTGGAATCGCAGGCA AGTATTAGAGAGGAAAGGGTGAGTGAGTTAATTGCGTTTCTTA GGAGGAGAGAAGGAGAAGTTGTTAAGATTTCAGATGTGATCT TCGGTTGCATGATCAACGTATTGGGCTCGGTCATTTTCAACCA AAGCGTGTATGATTTCGAAGGCAAAAAGGATACGGGCATGAA GGGTATGATTCGGGAGCTTATGATATTGGCAGCAACCCCAAAT CTGCCAGATTTTTACCCAATTTTTGATCGGTTTGACATTCAAGG CCTGAGAAGCACTACTTCGGCGTGTTGGAAGAAAATGAGCGA GTCATGGGCAGGTATAGTTAAAGAAAGAAGGGCCAGCAGAGA TCATTCAAGAAATGATTTCTTGGACGTGTTGATCCAAGCTAAC

Gene ID	Sequence
	TTCACCGACCCACAAATCGACGCCTTATTCTTGGAAATATTTG<br>CGCCTGGTAGAAGAGTTGTACGAAATTTCAGGTTTGTAGAAAT<br>GTGGCTTCAGCATGAGGGCTTGAATGACGTTGTGTTGCAGGGC<br>TAG
MdCYP80Q10P2.5	ATGGCTCTACTAGCTCTGTTCCTTATCTTTGCTCTTCCCATTCT<br>CTTGCTTTACTTCCTCAAACCCTCTTCTCCCAAAAACCTTCCTC<br>CAGGCCCATTTTCATGGCCTTTGCTTGGAACCTTGGGGCTGAA<br>ATCAAACGAAAAGCAAGCCCACGTCGTGCTAACCAATTTGGC<br>TCAAACCTATGGCCCGCTCATGCTGCTAAAGCTCGGAGTTAAG<br>CCTGTGATTGTGGCATCAACCCACGTTGCAGCCATGGAGATCC<br>TCAAGACACAAGACCACGTTCTGTCAGGTCGTTGCCCCCCGCA<br>TAGCGTGCAAGTCGAGGACTACATCGAGGCCTCAGTAGTTTG<br>GGCTGACCGTAATGAACACTGGAAGATGGTGAGGAAGATATG<br>CAGGACGGAACTGTTCTCTACTAAGATGTTGGAATCGCAGGC<br>AAGTATTAGAGAGGAAAGGGTGAGTGAGTTAATTGCGTTTCTT<br>AGGAGGAGAGAAGGAGAAGTTGTTAAGATTTCAGATGTGATC<br>TTCGGCTGCATGATCAACGTATTGGGCTCGGTTATTTTCAACC<br>AAAGCGTGTATGATTTCGAAGGCAAAAAGGATACGGGCATGA<br>AGGGTATGATTCGGGAGCTTATGATATTGGCAGCAACCCCAA<br>ATCTGCCAGATTTTTACCCAATTTTTGATCGGTTTGACATTCAA<br>GGCCTGAGAAGCACTACTTCGGCGTGTTGGAAGAAAATGAGT<br>GAGTCATGGGCAGGTATAGTGAAAGAAAGAAGGGCCAGCAG<br>AGATCATTCAAGAAATGATTTCTTGGACGTGTTGATCCAAGCA<br>AACTTCACCGACCCACAAATCGACGCCTTATTCTTGGAAATAT<br>TTGCGCCTGGTTCGGATAGTACTACGACCGCAATGGAGTGGGC<br>AATGACGGAGCTTATGAGGAATCCCGAGAAGCGCTTAAATAC<br>CAGATGTGGAGCTGGGGACCCTTATATGGCTTTAAAGTTAAAT<br>ATGACAAAAGCTTATGATCGAGTTGAGTGGGGATTCCTTAGGT<br>CAGTTATGGGCTCTATGGGTTTTGGCATAAAGGTGGTAGGCTT<br>AATTATGGAATGCATTGAATCTACACAATATTCGGTTATTATA<br>AATGGTAATCCATATGGTTTTTGGAAGCCCAACTGTGGTCTTC<br>GGCAAGGAGATCCGCTTTCGCCTTATCTTTTTATACTATGTGCT<br>GAAACTCTTTCAAAAATGCTTGATGGGGCCAATTCTCAAAATA<br>TTTTCCAAGGGGTGCGAGTAGCTCGTAGTGCACCTCAAGTTAC<br>TCACTTGTTCTTTGCGGATGATAGTTTGGTTTTCTGTCGGGCCA<br>AAGAAGATGAGTGCTAG
MdCYP80Q10.6	ATGGCTCTACTAGCTCTGTTCCTTATCTTTGCTCTTCCCATTCT<br>CTTGCTTTACTTCCTCAAACCCTCTTCTCCCAAAAACCTTCCTC<br>CAGGCCCATTTTCATGGCCTTTGCTTGGAACCTTGGGGCTGAA<br>ATCAAACGAAAAACAAGCCCACGCCGTGCTAACAAATTTGGC<br>TCAAACCTATGGCCCGCTCATGCTGCTAAAGCTCGGAGTTAAG<br>CCTGTGATTGTGGCATCAACCCACGATGCAGCCATGGAGATCC

Gene ID	Sequence
	TCAAAACACAAGACCACGTTCTGTCCGGTCGTTGCCCCCCGCA TAGCGTGCAAGTCGAGGACTACATCGAGGCCTCAGTAGTTTG GGCTGACCGTAATGAACACTGGAAGATGGTGAGGAAGATATG CAGGACAGAACTGTTCTCTACTAAGATGTTGGAATCGCAGGC AAGTATTAGAGAGGAAAGGGTGAGTGAGTTAATTGCGTTTCTT AGGAGGAGAGAAGGAGAAGTTGTTAAGATTTCAGATGTGATC TTCGGCTGCATGATCAACGTATTGGGCTCGGTTATTTTCAACC AAAGCGTGTATGATTTCGAAGGCAAAAAGGATACGGGCATGA AGGGTATGATTCGGGAGCTTATGATATTGGCAGCAACCCCGA ATCTGCCAGATTTTTACCCAATTTTTGATCGGTTTGACATTCAA GGCCTGAGAAGCACTACTTCCGCGTGTTGGAAGAAAATGAGC GAGTCATGGGCAGGTATAGTTAAAGAAAGAAGGGCCAGCAGA GATCATTCAAGAAATGATTTCTTGGACGTGTTGATCCAAGCTA ACTTCACCGACCCACAAATCGACGCCTTATTCTTGGAAATATT TGCGCCTGGTTCGGATAGTACTACGACAGCAATGGAGTGGGC AATGACGGAGCTTATGAGGAATCCCGAGAAGCGCTTAAATGT CCAAAACGAACTCAAAACGGTGATCGGAAACAGGCAAGTAGT GAAGGAATCAGATCTGCCAAACCTCCCATACCTCCATGCCTGC GTGAAGGAGAGCTTGAGACTGCACCCACCAGTGACGTTCCTG CTGCCTCATAAAGCAACCGAGACGTGCCAAGTGATGAACTAC ACGATTCCCAAGGGGACCCAGTTGATGGTCAACGCTTACGCG ATTGGTAGAGACCCAAAGACATGGGACGATCCGCTGTGTTTC AAGCCAGAACGGTTTTTGAACTCAGAAGTGGATTATCAAGGG AATGATTTTAGTTTAATACCGTTTGGATCTGGTAGGAGAATGT GCATAGGAATGCCTCTGGCTTCGAGGGTCGTGAGATTGATAAT AGCGTCTCTGATTCACAACTTTGATTGGAGCCTGCCTGGTGGG ATAAAACCGAGCGAGTTGGACATGCAGGAGATGTTCGAATTG GTGCTTCAGAAACACGTTCCTCTCTCTATCATACCCAATGCCA GAGCATAA
MdCYP80Q12	ATGGCTCTACTAGCTCTGTTCCTTCTCTTTGCTCTCCCCATTCT CTTGCTTTACTTCCTCAAAACCTCTTCTAATCCCAAAAACCTTC CACCAGGTCCCTTTTCATGGCCTCTCATTGGAACCTTAGTAAT GAAATTAAACGAGAAGCGACCTCACGTCGTGCTAACCAATTT GGCTCGAACCTATGGCCCGCTCATGCTGCTCACGTTTGGAATT GAACCCATTGCAGTCTGTTCCACCCCCGAAGCGGCAATGGAG ATGTTCAAGACACAAGACCGCGTTATGTCCGGTCGTTATGTGC CTCATAGCGTGCAAGTTAAGGGCTATATTGAGCACTCCATGGT GTGGGCCGATTGCAACGAGTACTGGAAGATGGTGAGGAAGAT ATACAGGACAGAGTTGTTCTCTACTAAGATGTTGGAAGCGCA AGTGAGTGTTAGAGAGGAGAAGGTGAAGGAGTTAATGGCGTT TATTAAAAGGAAGGAAGGAGAAGTTGTGAAGATTACAGATGT GGTCTATGGCTGCATTCTCAACATATTGGGCTCAGTTATTTTCA ACAAAAATGTGTATGATTTCGACGGCAAAACAGATAATGATG

Gene ID	Sequence
	CGGGCATGAAGGGTATGGTTCGACAGTTGATGACATTGGCAG GAATCCCAAATTTTCCTGATTTTTACCCAATTTTCGGTCGATTC GACCCCCAAGGTCTGAAAAAAAAAACTACAGAATGTGTGAAG AGGATGAATGAGTCATGGGCAGGTATAGTTAAAGAAAGAAGG GCCAGCAAAGATCACTCCGGAAATGATTTCTTGAATGTGTTGA TCCAAGCTAACTTCACCGACCCACAAATTGACTCCTTGCTCTT GGAAATATTTGGACCTGGTTCCGATTCCAGCACAAGTACAATT GAGTGGGCAATGGCGGACCTTATGCGGAATCCTGAGAAGCTC TTGAAAGTCCAAGATGAACTAGAAACTGTTATTGGGAGGAAC AGAGAAGTGAAGGAATCAGATCTACCCAATCTCCCATACCTC CATGCCTGCGTGAAGGAGACCTTGAGGCTGCACCCACCAGTG ACGTTTCTTCTGCCTCATCGATCAACCGAGACGTGCCAACTGA TGAACTACACGGTTCCGAAGGGAACCCAGACGACGGTCAACA CTTACGCCATTGGTAGAGACCCAAAGGCATGGGAGGACCCAC TGAGTTTCAAGCCAGAACGGTTTTTGAACTCCCAAGTGGACTA TCAAGGGAATGATTTCCATTACATACCGTTTGGAGCAGGGAG GAGAATTTGCCCAGCAGTGTCTCTGGCTTCAAGAGTGAGTCGA TTGATAATCGCGTCTTTCATTCACGACTTCGATTGGAGTTTGCC CAATGGGATGCAACCGAGTGAGCTGGACATGGAGGCCAAGTT TGGATTGGTGCTATGGAAAGATGATCCTCTCTGCATCATACCC CAAAACAAGAGCCCTATATAG
MdCYP80Q11P	ATGGCGCCACTAGCTCTCTTTCTCCTCTTCTTAGTCCCCATATT CCTATTGCTCCTCTTCAAACAATCTCCCAAAAACCTCCCTCCA GGCCCCTTCGCGTGGCCTCTGATCGGCACCTTACTGCCCAAGC TCAAGAAGCAGCCGCACGTCGAGCTCACCAAGTTGGCTCGGA CCTACGGCCCGCTCATGCTGCTCAAGTTCGGCGTTGAGCCCGT CGTGGTCGCGTCGAGCAATCAGGCCGCCATGGAGATCCTGAA GACGCAAGACCGCGTCCTCTCCGGCCGCTTTGCGCCGCACAGC GTTCAGATCAAGGGCTACGTCGAGCACTCCATGGTGTGGGCTG ACTGCACGGAGTACTGGAAGATGGTGAGGAAGATATGGAGGA CTGAGATCTTCTCCACGAAGATGTTGGACATGCAGGCGAGGA TTAGAGAGGAGAAGGTGAGTGAGTTGACGGCGTTTTTGAGGA GGAAGGAAGGAGAGGTTGTGAGGTTTGCAGATGTGATATTTG GGTGTATTCTGAACATTTTGGGATCGGTTGTTTTCAACCAAAA TGTGTATGATTTTGAGGATAAGTCGGATAATGATTTGGGCATG AAGGGGATGATTCGGCAGCTTATGATCTTGGCTGCTATTCCCA ACTTAGCTGATCTTTACCCAATTCTGGGACGCTCGGATTTTCA AGGTTTGAGAAAGGCTTCTACGGCGTGTGTGAAGAGGATGAA CGAGTCGTGGGCGGCTATAGTGAAAGAAAGAAGAGCCAGCGG AGATCATTCCAGAAATGATTTTTTGGATATGTTGATTCAAGCC GACTTCACCGATCCACAAATCGACGCAATGCTCTTGGAAATAT TTGGGCCTGGTTCGGATACTAGCACATCTACAATAGAGTGGGC GATGGCGGAGCTTATGAGGAGCCCTGAGAAGCTATTGAAAAT

Gene ID	Sequence
	CCAAGACGAGCTCAAAACGGTGATTGGGAACAGAGAAGTGAA<br>TGAATCAGATCTGTCCAACCTCCCATACCTCCACGCCTGCATG<br>AAGGAGACCTTGAGACTACACCCACCAGTGACTTTCCTTCTGC<br>CTCATCGAGCAACCGAGACGTGCCAAGTGATGAACTACACGG<br>TTCCGAAGGGGTCCCAGTTGATGGTCAACACTTACGCGATTGG<br>GAGAGATCCAAAGACATGGGACGACCCAAACTGTTTCATGCC<br>AGAACGGTTTTTGAACTCAGAAGTTGACTACCAAGGCAATGA<br>TTTTCATTACATACCGTTTGGAGCTGGGAGGAGAATTTGCCCA<br>GGATTGTCTCTGGCGTCTAGAGTGGTGAGATTGATTTTGGCTT<br>CTTTGATTATCAATTTTGACTGGAGCCTGCCCAATGGGATGCA<br>CCCGAGTGAGCTAGACATGCAGGACAAATTCGGATTGGCTCTT<br>CTCAAGGACATTCCTCTCCTGCTGGTGCCCAAAGTAAGAAGTA<br>CATAA
MdCYP80Q11	ATGGCGCCACTAGTAGCTCTGTTTCTTCTCTTCTTAGTTCCCAT<br>ATTTATTTTCTTGCGTCTCTTCAAACCATCTCCTAAAAACCTCC<br>CTCCTGGCCCCTTCGCATGGCCTCTGATAGGAACCTTACTGCC<br>CAAACTGAAGAAACAGCCGCACGTCGAGCTAACCAAGCTGAG<br>TCGGACCTACGGCCCGCTCATGCTGCTCAAGTTTGGCGTGGAG<br>CCTGTGGTAGTCGCGTCGAGCCAAGAGGCCGCTATGGAGGTC<br>CTCAAGACCCAAGACCGCGTTCTTTCCGGCCGCTTTGCGCCTC<br>ACAGCGTTCAGATCAAGGGCTACGTCGAGCACTCCATGGTGT<br>GGGCCGACTGCACGGAGTACTGGAAGATGGTGAGGAAGATAT<br>GGAGGACTGAGCTCTTCTCTACTAAGATGTTGGACGCGCAGGC<br>GAGGATTAGAGAGGAGAAGGTGAGTGAGTTGATGGTGTTTTT<br>GAGGAGGAAGGAAGGAGAGGTTGTGCGATTTGCAGATGTGAT<br>CTTTGGTTGCATTTTGAACATTTTGGGATCGGTTATTTTCGACC<br>AAGATGTGTATGATTATAAGGATAAGACGGATAATGATAAGG<br>GCATGAAGGGCATGATTCGACAGCTTATGATCTTGGCTGCTAT<br>TCCAAACTTAGCTGATCTTTACCCGATTCTCGGGCGTTCGGAT<br>TTTCAAGGTTTGAGAAAAGCTTCTGCTGCTTGTGTGAAGAGGA<br>TGAACGAGTCTTGGGCGGCTATAGTGAAGGAAAGAAGAGCCA<br>GCGGAGATCACTCCAGAAATGATTTCTTGGACGTCTTGATTCA<br>AGCCGACTTCACCGACCCACAAATCGACGCCTTGCTCTTGGAA<br>GTATTTGGGCCTGGATCGGATACTAGCACATCTACGATAGAGT<br>GGGCGATGGCGGAGCTCATGAGGAGTCCTAAGAAGCTCTTGA<br>AAGTTCAAGACGAGCTGAAGACGGTGATTGGGACGAACAGAG<br>AAGTGAAGGAATCAGATCTGTCGAACCTCCCATATCTCCATGC<br>TTGCGTGAAGGAGACGTTGAGGCTGCACCCACCAGTGACGTTT<br>TTGCTGCCTCATCGAGCCACTGAGACGTGCCAAGTGATGAACT<br>ACACGGTCCCGAAGGGAACCCAGTTGATGGTCAACACTTACG<br>CTATAGGGAGAGACCCAAAGACATGGGAGGGTCCACTGTGTT<br>TCGAGCCAGAACGGTTTTTGAACTCAGAAGTGGACTATCAAG<br>GGAATGATTTCCATTACATACCGTTTGGAGCTGGGAGGAGGAT

Gene ID	Sequence
	TTGCCCAGGACTGTCTCTGGCTACTAGAGTGGTGAGATTGATA<br>TTGGCTTCTTTGATTGTCAACTTTGATTGGAGTTTGCCCAATGG<br>GATGCACCCGAGCGAGCTGGACATGCAGGACAAGTTCGGATT<br>GGCGCTTCTCAAGGACATTCCTCTCTTGGTGGTGCCCAAACTC<br>AGAACTACTTGA
MdCYP80G8P	ATGGATCTTGCAGTACTATTATCCCTCTTCGTTCCCGCTATCCT<br>CATCTACCTTCTCTTGAAACCCAAAAACCTCCCACCAGGCCCA<br>CGCTCATGGCCTCTTCTAGGCAACTTGCTAACCGTCCTACGCA<br>GCCACGTACCCCTTCACATCACCCTCACAGACCTGGCTCGAAC<br>CCACGGCCCGCTCATGCTCGTCACGCTCGGGACCCAGCCCACC<br>GTCCTGGCCTCAACCAGCGAGGCCGCCATGGAGATCCTCAAG<br>ACCCACGACCGAGCCCTCTCGGGCCGCCACATACGCATGAGC<br>TTCCGCTTAAAAGAGATGAACAAGCACTCGCTCGTCTGGTCCA<br>ACTGCACAGACACCTGGAAGCTCTTGAGAAAAATTGCTAGAA<br>CCGAGATCTTCTCGCCCAAGATGCTGCAGATTCAGGAGCACGT<br>GAGGGAGCAGAAGGTGTTGGAGCTTGTGGAGTTCTTGAGAGG<br>GAGAGAGTACTTAGGGAAGGCGGTGAAGATCAGCCAGTTCGT<br>GTTTGGGACGCTGTTGAATATAATTGGGAACGTCGTGTTCTCG<br>AAAGACGTTTTTGGGTTTAGTTGTGATGAGGGAGGGGATGAG<br>ATTGGGATGCAGAGTTTGATACGGGAGCTGCTGATGATTGGG<br>GCGAGCCCTAACATTGCTGAGTTCTATCCGATTCTGGAGGGGT<br>TGGATTTGCAAGGGTTGAAGCGGCGGTGCAAGGACCGAGTTG<br>ACCGAGTTAACAAGCTGTGGGAAGGGACGGTCAAAGAGAGG<br>AGACTGAAAAGGAGTGGTGAGTCAAAAGATATGTTGGACGTT<br>CTTCTTGATAATGGGTTTGATGACGTTCAGATCAACGTCTTGTT<br>CTTGGAAACATTTGGCCCTGGTTCAGAGACAAGCAGTGCAAC<br>CATAGAGTGGGTGATGGCAGAGCTGATCAAGAACCCAGACAA<br>ACTAGCCAAGGTCCGAACAGAACTAGAACAAGTAGTTGGGCT<br>GCACTCACAAGTGAAAGAAAGTCACCTCCCTCATCTCCACTAC<br>CTCCAAGCCTGTGTTAAAGAGACAATGAGGCTGCACCCAGCC<br>GCACCGTTCCTTCTCCCACACCGAGCTGTCGAGACGTGCCGAG<br>TCATGGGCTACACCATCCCTAAAGACTGCCAAGTCCTGGTGAA<br>CGCTTACGCCATTGGAAGGGACCCAAACATATGGAAAGACCC<br>ATCTAGGTTCAAACCTGAGCGCTTCTTGGAGTCCAGTGTGGAC<br>TACAATGGGAACCATTTCGAGTTCATACCATTTGGGTCGGGGA<br>GGAGAATTTGCATTGGAATGCCTCTAGCGACTCGGACTGTCCC<br>TTTGATCGTGTCCTCTTTAGTGCACAACTTTGATTGGAGTTTAC<br>CAGATGGAAAACGCCCTGAAGAGTTGGTGATGAATGAGATGC<br>TAAGCTTGTCACTAGCTATTGATCCAAGTCTGTCTATTATCCCT<br>AAAGTGAGAGGATAG
MdCYP80G8	ATGGATCTAACTCTAGCGCTGTTATCCCTCTTCATTCCCGCTAT<br>CCTCCTCTACCTTCTCTTCAAACCCACAAACCTCCCACCAGGC

Gene ID	Sequence
	CCTCGCTCATGGCCTCTTATCGGCAACTTGCTAACCGTCCTAC GCAGCCACGCACCCCTTCACATCACCCTCACAGACCTGGCTCG AGCCCACGGCCCGCTGATGCTCGTCACCCTCGGGACCCAGGCC ACCGTCCTGGCCTCAACCAGAGAGGCAGCCATGGAGGTCCTC AAGACCCACGACCGAGCCCTCTCGGGCCGCCACATACGCATG AGCTTCCGCTTAAAAGAGATGAACAAACACTCTCTCGTCTGGT GCAACTGCACAGACACCTGGAAGCTCTTGAGAAAATTAGCTA GGACTGAGATCTTCTCTCCCAAGATGCTGCAGATTCAGGAGCA CGTGAGGGAGCAGAAGGTGTTGGAGCTAGTGGAGTTCTTGCG AGGGAGAGAAGGGAAGGTTGTGAAGATCAGCCAATTCGTGTT TGGGACGCTGTTGAACATAATTGGGAATGTCGTGTTCTCGAAA GACGTTTTTGGGTTTAGTGATGAGGGAGGTGATGAGATTGGG ATGCAGAGTTTGATTCGGGAGTTGCTGATGATAGGAGCGAGT CCGAACTTGGCTGAGTTTTATCCGATTCTGGAGGGGTTGGATT TGCAAGGGTTGAAGCGGCGGTGTAAGGACCGAGTTGAGCGAG TTAACAAGCTGTGGGAGGGGACGGTCAAGGACAGGAGGAAG ACTAGGAGCGGGGAGTCAAAGGACATGTTGGACGTTCTTCTT GATAATGGGTTTGACGACGTTCGGATCAACGTCCTGTTCTTGG AAACATTTGGCCCTGGTTCAGAGACAAGCAGTGCAACCATAG AGTGGGTGATAGCTGAGCTGATCAAGAATCCGGACAAACTAG CCAAGGTCCGAAAAGAATTAGAACAAGTAGTTGGGCTGCATT TACATGTGAAGGAAAGCCACCTCCCTCATCTCCACTACCTCCA AGCCTGTGTCAAAGAGACAATGAGGCTGCACCCAGCCGCACC GTTCCTTCTCCCACACCGAGCCGTCGAGACCTGCCGAGTCATG GGTTACACCATCCCTAAAGACTACCAAGTCCTTGTGAACGCTT ACGCCATTGGAAGGGACCCCAACACATGGAAAGACCCATCGA CTTTCAAACCTGAGCGCTTCTTGGAGTCCAGTGTGGACTACAA TGGGAACCATTTCGAGTTCATACCGTTTGGGTCTGGGAGGAGA ATTTGCATCGGAATGCCTCTAGCAACTCGAACTGTTCCTTTGA TTGTGGCTTCTTTAGTGCACAGCTTTGATTGGACCCTACCGGA TGGAAAGCGCAATGAAGAATTGGTGATGAATGAGATGCTAAG CTTGTCACTAGCTATTGATCCAAGTCTCTCTATCATCCCTAAAG TGAGAGGATAA
MdCYP80G11	ATGTATGATCTTGGCGGAGGCAAAGGAGATATGGTGGGACTA GAGTTGTTGATTCGCGAGCTGCTCGTGATTGGTGCCACCCCTG ACCTTGCTGACTACTATAGTTTTCTTAAATGGCTAGACTCACA AGGGTTGAAAAAGCAAGCCTTGGAGCGACTCGTCAAAGTTGA TAAACTCTGGGAGTCCACAGTGAAGGATAGAAGACAGCAGAA GAAGAGTGTTAGTAGTACCGGTGATCAAGAATGCACGGACAT GTTGGACGTGCTTATTGCCAACAACTTCACCGACCTTCAAATC GACGCTATATTCTTGGAAACTTTTGGGCCTGGTTCTGAGAGCA GCAGCGCGACGGTGGAGTGGGTGATGTCTGAGCTAATCAAGC ATCCAGACGCACTGGCGAAAGTCCGCCAGGAATTGGACGAGG

Gene ID	Sequence
	AGTACTTGAAGCAATCCAAGGTGACTGGGCCTCGACTCACTTA<br>TCTCAACGCGGTGATCAAAGAGACAATGAGACTGCACCCAGC<br>GATCCCGTTCATGCTTCCTCACCGAGCCGTGGAGACATCCCAA<br>GTGCTGGGCTATACCATCCCAAAAGGCTTCCAAGTCCAGTTGA<br>ACGCTTACGCGATCGGGAGAGACCCAAAAGCCTGGAAAGACC<br>CAAATACGTTTCGGCCAGAGCGGTTCTTGGAGTCCGATATCGA<br>TTACCAGGGGAACCACTTCGAGCTTATACCGTTCGGTGCTGGA<br>AGAAGGATTTGTCCTGGGCTGCCTCTGGCCCTGAAGAACACGC<br>CATTAATAGTGTCCTCTCTGATTCATGGGTTTGATTGGAGCCTC<br>CCTGATGGCCTTACGCACGAGCAGCTTCATATGAATGAGACG<br>GTCTCGATTGCACTCACCAAGGATCCGAGTCTCTGCCTGATCC<br>CCAAAGTGAGGGTCGATCCACGTCTTAATTAG
MdCYP80G10	ATGGATACAGTATTTGAAGTGCTTTGCTGGTTCATTTTCTTCCT<br>GAGCACTATTGTTGTCTCGTATAATCTTTTCCAACCGAGAGTA<br>GTCAAGAACCTCCCACCGGGCCCTCGTCCGTGGCCTCTTCTAG<br>GCAACCTGGTGACCATCTTCAGCAGCAAGACCCCACCGCATAT<br>CACCTTCGCCAACCTAGCTCGTGCTCATGGCGGGCTAATGCTG<br>CTCTGGTTCGGCCACAAGCCCGTGGTGTTCGTCTCGGACAAGG<br>AGGCCGCCATGGAGGTTCTCAAAACTCACGACCGAGCCCTCTC<br>AGGCCGCCATGTTCCAGCGAGCTTCCGCATCCAGGAGAAGAT<br>CCAACACTCTCTTGTCTGGTCCGACTGCACCGATTACTGGAAG<br>CTCGTGCGAAGGACGCTCCGAACCGAGGTCTTTTCTCCGAAGA<br>TGTTGCGAATTCAGGAGCAAGTGAGAGAGAAGAAGGTGGGAG<br>AGCTGATGGAGTTCATGAGGAGCAAAGAAGGGCAGGTGGTGA<br>CCATCAGGCCTGTAGTGTTTGGGACTATTTTGAATATACTAGG<br>GAATGCCATTTTCTCTCGCGATATGTATGATCTTGGCGGAGGC<br>AAAGGAGATATGGTGGGACTAGAGTTGTTGATTCGCGAGCTG<br>CTCGTGATTGGTGCCACCCCTGACCTTGCTGACTACTATAGTTT<br>TCTTAAATGGCTAGACCCACAAGGGTTGAAAAAGCAAGCTTT<br>GGAGCGACTCGTCAAAGTTGATAAACTCTGGGAGTCCACGGT<br>GAAGGATAGAAGACAGCAGAAGAAGAGTGGTAGTAGTACCG<br>GTGATCAAGAATGCAAGGACATGTTGGACGTGCTTATTGCCA<br>ACAACTTCACCGACCTTCAAATCGACGCTATATTCTTGGAAAC<br>TTTTGGGCCTGGTTCTGAGAGCAGCAGCGCGACGGTGGAGTG<br>GGTGATGTCTGAGCTAATCAAGCATCCGGATGCAATGGCGAA<br>AGTCCGCCAGGAATTGGACGAGGAGTACTTGAAGCAATCCAA<br>GGTGACTGGGCCTCGACTCACTTATCTCAACGCGGTGATCAAG<br>GAGACAATGAGACTGCACCCAGCGATCCCGTTCATGCTTCCTC<br>ACCGAGCCGTGGAGACATCCCAAGTGCTGGGCTATACCATCC<br>CAAAAGGCTTCCAAGTCCAGTTGAACGCTTACGCGATCGGGA<br>GAGACCCAAAAGCCTGGAAAGACCCAAATACGTTTCGGCCAG<br>AGCGGTTCTTGGAGTCCGACATCGATTACCTGGGGAACCACTT<br>CGAGCTTATACCGTTCGGTGCTGGAAGAAGGATTTGTCCTGGG

Gene ID	Sequence
	CTGCCTCTGGCCGTGAAGAACACGCCATTGATTGTTTCGTCTC<br>TGGTTCGTGGGTTTGATTGGAGCCTCCCTGATGGCCTTACGCA<br>CGAGCAGCTTCATATGAATGAGACGGTCTCGATTGCACTCATC<br>AAGGATCCGAGTCTCCGCCTCATCCCCAAAGTCAGGGTCGATC<br>CGCGTCTTAATTAG
MdCYP80G9	ATGGACGCAGTGCTTGCAATCCTCTACCTCACCATCTTCTTCCT<br>CACCATCCTCCTCGTTAAGCTTTTCCAACACAAATCACACAAG<br>TCGAACCAACTCCCACCAGGGCCTCGTCCATGGCCTATCTTAG<br>GCAACTTGGTGAGCATCTTGGGGAGCAAGACCCCACCCCACA<br>TCACCTTCGCGAACCTCGCTCGAGCCCACGGCGGCCTCATGCT<br>CCTCTGGCTCGGCCAGAAACCCTTGGTGGTGGTCTCAGACAAG<br>GAAGCCGCCGTGGAAGTCCTCAAGACCCACGACCGAGTCCTC<br>TCCGGCCGTTCCATCACCGTGAGCTTCCGCTTCCGTGAGAAAA<br>CCGAGTACTCTCTCGTTTGGGCCGACTGCAACGATTACTGGAA<br>GCTCCTTCGCAGGATTCTCCGAACCGAGATCTTTTCGCCCAAG<br>ATGTTGAAGATTCAGGAGCGAGTGAGAGAGCAGAAGGTAACG<br>GAGCTGATGGAGTTGATGAGGAGTAGCGAAGGGAAAGAGGT<br>GACGATCAGGCCGTTGGTGTTTGGGACTATATTGAATATATTA<br>GGGAACGCAGTGTTCTCAAAGGACATGTTTGAGCTTGGTGGG<br>AGAGGAGACAAAGTTGGGTTGGAGCAGCTGATTCGCGAGCTG<br>CTCACCATCGGTTCGACCCCTAATATAGCAGCTTTCTTTCCCAT<br>TTTCGAGAAGCTCGATCCACAAAGGCTTAAAAAGCGAACTTG<br>GGAGCGACTTGTTAAAATAGACAAACTTTGGGAGTCCATAGT<br>GAAGGAACGGAGACAGCAGGAGAGGAGCGAAGAATGCAAGG<br>ACATGTTGGACGTCCTTCTCGCCAACGACTTCACTGACCCGCA<br>GATCGACAATATATTCTTGGAAACTTTTGGTCCCGGCTCTGAG<br>AGCAGCAGCGCAACAGTGGAGTGGGTGATGTCGGAGCTCATA<br>AAAAACCCACAAACATTTGCGAAACTCCGCCAAGAACTGGAC<br>AACGAGTTCGGCCAATCCAAAGTGATTACTGGATCTCGCCTCA<br>CAAACCTTCCATTCCTGAGCGCAGTGATCAAGGAGACCATGC<br>GATTGCACCCAGCAATTCCTTTCATGCTCCCTCACAGAGCAGT<br>AGAGACATGCGAGGTGATGGGCTACACAATCCCCAAGGACAT<br>GGAGATCCAGTTGAACGCTTACGCGATCGGGAGAGACCCAAA<br>AGCCTGGAAAGACCCAAATACGTTTCGACCAGAGCGGTTCTT<br>GGAGTCCGATATCGATTACCAGGGGAACCACTTTGAGCTTATA<br>CCGTTCGGTGCTGGAAGAAGGGTTTGTCCTGGGCTGCCTCTGG<br>CCGTGAAGAACACACCGTTGATTGTGTCCTCTCTGGTTCATGG<br>GTTTGATTGGAGCCTCCCTGATGGGCTTACGCACGAGCAGCTT<br>CAGATGAACGAGGTACTCTCTGTTGCGCTGACTAAGGACCCCA<br>GTCTCTGTCTCATCCCCAAAGTCAGGGTCTGGGCTTCCTAA

Table S11. The list of information of chemical standards.

Index	Compounds	M. Wt (g/mol)	Molecular formula	Manufacturer information
**1**	Dopamine	153.18	$C_8H_{11}NO_2$	Shanghai Macklin Biochemical Co., Ltd.
**2**	4-HPAA	136.15	$C_8H_8O_2$	Toronto Research Chemicals Inc.(Toronto, Canda)
**3**	(*S*)-Norcoclaurine	271.31	$C_{16}H_{17}NO_3$	Toronto Research Chemicals Inc.(Toronto, Canda)
**5**	(*S*)-Coclaurine	285.34	$C_{17}H_{19}NO_3$	Sichuan Victory Biological Technology Co., Ltd.(Sichuan, China)
**6**	(*S*)-*N*-Methylcoclaurine	299.36	$C_{18}H_{21}NO_3$	Toronto Research Chemicals Inc.(Toronto, Canda)
**8**	(*S*)-Reticuline	329.39	$C_{19}H_{23}NO_4$	BioBioPha Co., Ltd. (Yunnan, China)
**9**	(*S*)-Scoulerine	327.37	$C_{19}H_{21}NO_4$	BioBioPha Co., Ltd. (Yunnan, China)
**10**	(*S*)-Tetrahydrocolumbamine	341.40	$C_{20}H_{23}NO_4$	Shanghai Yuanye BioTechnology Co., Ltd. (Shanghai, China)
**14**	(*S*)-Tetrahydropalmatine	355.43	$C_{21}H_{25}NO_4$	Chengdu Biopurify Phytochemicals Ltd.
**16**	Magnoflorine	342.41	$C_{20}H_{24}NO_4$	Chengdu Biopurify Phytochemicals Ltd.
**18**	Sinoacutine	327.37	$C_{19}H_{21}NO_4$	Chengdu Push Bio-technology Co., Ltd. (Chengdu, China)
**19**	Sinomenine	329.39	$C_{19}H_{23}NO_4$	Chengdu Push Bio-technology Co., Ltd. (Chengdu, China)
**23**	(*S*)-Magnocurarine	314.40	$C_{19}H_{24}NO_3$	Shanghai Yuanye BioTechnology Co., Ltd. (Shanghai, China)
**24**	Tetrandrine	622.75	$C_{38}H_{42}N_2O_6$	Chengdu Push Bio-technology Co., Ltd. (Chengdu, China)
**25**	(*R*)-*N*-Methylcoclaurine	299.36	$C_{18}H_{21}NO_3$	BioBioPha Co., Ltd. (Yunnan, China)
**26**	Dauricine	624.76	$C_{38}H_{44}N_2O_6$	Chengdu Biopurify Phytochemicals Ltd.
**27**	Daurisoline	610.75	$C_{37}H_{42}N_2O_6$	Chengdu Biopurify Phytochemicals Ltd.
**28**	Dauriciline	596.71	$C_{36}H_{40}N_2O_6$	Chengdu Biopurify Phytochemicals Ltd.
	Umbelliferone	162.14	$C_9H_6O_3$	Chengdu Push Bio-technology Co., Ltd. (Chengdu, China)
